# Rotavirus vaccine efficacy up to 2 years of age and against diverse circulating rotavirus strains in Niger: Extended follow-up of a randomized controlled trial

**DOI:** 10.1371/journal.pmed.1003655

**Published:** 2021-07-02

**Authors:** Sheila Isanaka, Céline Langendorf, Monica Malone McNeal, Nicole Meyer, Brian Plikaytis, Souna Garba, Nathan Sayinzoga-Makombe, Issaka Soumana, Ousmane Guindo, Rockyiath Makarimi, Marie Francoise Scherrer, Eric Adehossi, Iza Ciglenecki, Rebecca F. Grais

**Affiliations:** 1 Department of Research, Epicentre, Paris, France; 2 Department of Nutrition, Harvard T.H. Chan School of Public Health, Boston, Massachusetts, United States of America; 3 Department of Global Health and Population, Harvard T.H. Chan School of Public Health, Boston, Massachusetts, United States of America; 4 Department of Pediatrics, University of Cincinnati, Cincinnati, Ohio, United States of America; 5 Division of Infectious Diseases, Cincinnati Children’s Hospital Medical Center, Cincinnati, Ohio, United States of America; 6 BioStat Consulting, Jasper, Georgia, United States of America; 7 Epicentre, Niamey, Niger; 8 National Hospital, Niamey, Niger; 9 Operational Center Geneva, Médecins Sans Frontières, Geneva, Switzerland; Makerere University Medical School, UGANDA

## Abstract

**Background:**

Rotavirus vaccination is recommended in all countries to reduce the burden of diarrhea-related morbidity and mortality in children. In resource-limited settings, rotavirus vaccination in the national immunization program has important cost implications, and evidence for protection beyond the first year of life and against the evolving variety of rotavirus strains is important. We assessed the extended and strain-specific vaccine efficacy of a heat-stable, affordable oral rotavirus vaccine (Rotasiil, Serum Institute of India, Pune, India) against severe rotavirus gastroenteritis (SRVGE) among healthy infants in Niger.

**Methods and findings:**

From August 2014 to November 2015, infants were randomized in a 1:1 ratio to receive 3 doses of Rotasiil or placebo at approximately 6, 10, and 14 weeks of age. Episodes of gastroenteritis were assessed through active and passive surveillance and graded using the Vesikari score. The primary endpoint was vaccine efficacy of 3 doses of vaccine versus placebo against a first episode of laboratory-confirmed SRVGE (Vesikari score ≥ 11) from 28 days after dose 3, as previously reported. At the time of the primary analysis, median age was 9.8 months. In the present paper, analyses of extended efficacy were undertaken for 3 periods (28 days after dose 3 to 1 year of age, 1 to 2 years of age, and the combined period 28 days after dose 3 to 2 years of age) and by individual rotavirus G type. Among the 3,508 infants included in the per-protocol efficacy analysis (mean age at first dose 6.5 weeks; 49% male), the vaccine provided significant protection against SRVGE through the first year of life (3.96 and 9.98 cases per 100 person-years for vaccine and placebo, respectively; vaccine efficacy 60.3%, 95% CI 43.6% to 72.1%) and over the entire efficacy follow-up period up to 2 years of age (2.13 and 4.69 cases per 100 person-years for vaccine and placebo, respectively; vaccine efficacy 54.7%, 95% CI 38.1% to 66.8%), but the difference was not statistically significant in the second year of life. Up to 2 years of age, rotavirus vaccination prevented 2.56 episodes of SRVGE per 100 child-years. Estimates of efficacy against SRVGE by individual rotavirus genotype were consistent with the overall protective efficacy. Study limitations include limited generalizability to settings with administration of oral polio virus due to low concomitant administration, limited power to assess vaccine efficacy in the second year of life owing to a low number of events among older children, potential bias due to censoring of placebo children at the time of study vaccine receipt, and suboptimal adapted severity scoring based on the Vesikari score, which was designed for use in settings with high parental literacy.

**Conclusions:**

Rotasiil provided protection against SRVGE in infants through an extended follow-up period of approximately 2 years. Protection was significant in the first year of life, when the disease burden and risk of death are highest, and against a changing pattern of rotavirus strains during the 2-year efficacy period. Rotavirus vaccines that are safe, effective, and protective against multiple strains represent the best hope for preventing the severe consequences of rotavirus infection, especially in resource-limited settings, where access to care may be limited. Studies such as this provide valuable information for the planning of national immunization programs and future vaccine development.

**Trial registration:**

ClinicalTrials.gov NCT02145000.

## Abstract 2

Sheila Isanaka and co-workers report extended follow-up of a rotavirus vaccine trial in Niger.

## Author summary

**Why was this study done?:**

- The World Health Organization (WHO) recommends rotavirus vaccine use in all countries, but several questions remain for countries that have not yet implemented rotavirus vaccination or are weighing different vaccine options. In resource-limited settings, where rotavirus vaccine delivery has important cost implications, evidence for protection beyond the first year of life and against the evolving variety of rotavirus strains is important.
- We previously reported on the efficacy and safety of Rotasiil (Serum Institute of India, Pune, India), a heat-stable, affordable oral rotavirus vaccine that was WHO prequalified in 2018, against severe rotavirus gastroenteritis (SRVGE) among healthy infants in Niger.

**What did the researchers do and find?:**

- In the present analysis, we assessed the extended and strain-specific vaccine efficacy of Rotasiil in children up to 24 months of age.
- Rotasiil provided protection against SRVGE in infants through an extended follow-up period and against a changing pattern of rotavirus strains during the 2-year efficacy period.

**What do these findings mean?:**

- Vaccines that are safe, effective, and protective against multiple strains represent the best hope for preventing the severe consequences of rotavirus infection, and can be expected to greatly reduce the burden of rotavirus disease.

## Introduction

Rotavirus is the leading cause of childhood diarrhea and a major cause of diarrhea-related hospitalizations and mortality in children less than 5 years of age [1,2]. To reduce this substantial burden, prevention through vaccination is essential, and the World Health Organization (WHO) recommends rotavirus vaccine use in all countries [3]. Currently, 106 countries have introduced rotavirus vaccines into their national childhood immunization programs [4], and swift reductions in all-cause diarrhea and rotavirus hospitalizations have already been seen [5–7].

While inclusion of any rotavirus vaccine in national immunization programs is essential and evidence of impact has encouraged countries with high diarrheal burden to introduce rotavirus vaccination at scale, several questions remain for countries that have not yet done so or are weighing different vaccine options [8,9]. The choice of which vaccine to adopt necessitates weighing several factors, including presentation, schedule of administration, storage requirements, cost, and efficacy. In resource-limited settings, where rotavirus vaccine delivery in the national immunization program has important cost implications [10], evidence for protection beyond the first year of life and against the evolving variety of rotavirus strains is important.

We assessed the efficacy and safety of a heat-stable, affordable oral rotavirus vaccine—which was later WHO prequalified in 2018—against severe rotavirus gastroenteritis (SRVGE) among healthy infants in Niger [11]. The present study reports on extended and strain-specific vaccine efficacy up to 24 months of age.

## Methods

We conducted a double-blind, placebo-controlled randomized phase III event-driven trial in Madarounfa, Niger, to assess the efficacy and safety of Rotasiil (Serum Institute of India, Pune, India) against SRVGE in healthy infants (ClinicalTrials.gov identifier: NCT02145000). Primary efficacy and safety results were previously reported after accumulation of the target number of primary endpoint cases [11,12]. In brief, among the 3,508 infants included in the per-protocol efficacy analysis, there were 31 and 88 cases of SRVGE in the vaccine and placebo group, respectively (2.14 and 6.44 cases per 100 person-years; vaccine efficacy 66.7%, 95% CI 49.9% to 77.9%). There was no difference in the risk of adverse events (68.7% of the infants in the vaccine group and 67.2% of the infants in the placebo group) or serious adverse events (8.3% of the infants in the vaccine group and 9.1% of the infants in the placebo group), including death (*N* = 27 in the vaccine group and *N* = 22 in the placebo group). No child had confirmed intussusception. At the time of primary analysis, the data and safety monitoring board (DSMB) concluded that the primary hypothesis was satisfied and advised administration of study vaccine to all children randomized to placebo still under follow-up (including 92 children in the per-protocol population and 101 children in the intent-to-treat population of this analysis; mean age at the time of first dose of study vaccine = 23 months).

### Study design and participants

Infants were randomized in a 1:1 ratio to receive 3 doses of Rotasiil or placebo at approximately 6, 10, and 14 weeks of age. Infants were eligible for enrollment if parents resided in the study area and intended to remain in the study area for 2 years. Infants were excluded if they had a known history of a serious medical condition, congenital abdominal disorder, intussusception, or abdominal surgery; received any other rotavirus vaccine, corticosteroid treatment, blood transfusion, or blood product; or had any other condition that the site principal investigator judged would interfere with protocol adherence or the parent’s ability to give informed consent.

The trial was conducted in accordance with Good Clinical Practice guidelines.

The study protocol (S1 Protocol) was approved by the ethics committee of the World Health Organization (Geneva, Switzerland), the Western Institutional Review Board (Olympia, WA, US), Comité Consultatif National d’Ethique (Niamey, Niger), the Comité de Protection des Personnes (Ile-de-France, France), and Hôpitaux Universitaires de Genève (Geneva, Switzerland). Written informed consent was obtained from each child’s parent or legal guardian. A CONSORT checklist is available (S1 CONSORT Checklist).

### Study vaccine

Rotasiil (bovine rotavirus pentavalent vaccine [BRV-PV]) is a live attenuated bovine–human (UK) reassortant rotavirus vaccine containing rotavirus serotypes G1, G2, G3, G4, and G9 (>5.6 log10 fluorescent focus units/serotype/dose) and is delivered in lyophilized form with 2.5 mL of citrate bicarbonate buffer added for reconstitution before oral administration. Placebo, also manufactured by the Serum Institute of India, contained the same constituents as the active vaccine but without the viral antigens. Vaccine and placebo were identical in appearance and packaging.

Study vaccine and placebo were administered at a health center by study physicians. The initial dose was given at 6–8 weeks of age, with each subsequent dose at a 4-week interval (−1 to +4 weeks). Vaccination was delayed only if the child was unable to swallow, had a history of vomiting within the last 24 hours, or required immediate hospitalization. No specific instructions about breastfeeding were given around the time of administration. Children were referred for administration of routine Expanded Programme on Immunization vaccinations (oral poliovirus vaccine [OPV], pentavalent vaccine diphtheria–tetanus–whole cell pertussis, *Haemophilus influenzae* type b, hepatitis B, pneumococcal conjugate vaccine, measles, and yellow fever) and vitamin A supplementation by the local health authority free of charge.

### Randomization and blinding

Unique allocation numbers were prepared using a computer-generated random number list with permuted blocks of random sizes (DiagnoSearch LifeSciences, Mumbai, India). Vaccine and placebo packages were labeled with allocation numbers and provided to sites in identical presentations. Study physicians assigned allocation numbers to participants in sequential order as they were enrolled. Investigators, children, parents/guardians, sponsor representatives, laboratory personnel, the DSMB, and the study statistician were blinded to treatment assignment until DSMB review of the primary analysis.

### Assessment of efficacy

We defined gastroenteritis as the passage of 3 or more looser-than-normal stools in a 24-hour period with or without vomiting. Severity was defined using the 20-point Vesikari clinical scoring system [13], with a score of 11 or more classified as severe and a score of 15 or more classified as very severe. Gastroenteritis episodes were classified as 2 separate episodes if there was an interval of 5 or more consecutive diarrhea-free days between episodes.

Cases of gastroenteritis were captured through facility- and home-based surveillance. Caregivers were informed about the signs and symptoms of gastroenteritis and asked to seek care at a local facility (including 1 hospital, 5 health centers, and 12 health posts) if any episode of gastroenteritis was suspected. Episodes not reported to a health facility or a community health agent were captured during scheduled weekly home visits. Daily home visits were conducted until resolution was confirmed with ≥5 consecutive diarrhea-free days. If any gastroenteritis episode was found to require medical attention, study staff referred the child to a study health center for management free of charge.

Stool samples were collected for all episodes of gastroenteritis up to 7 days after the last day of symptoms. Specimens were transported in freezer packs at 2–8°C on the same day and frozen at −80°C until testing within 5 days. Rotavirus antigen in stool was detected by enzyme immunoassay (Premier Rotaclone, Meridian Bioscience) at the Epicentre Maradi laboratory. All rotavirus-positive stool samples were shipped to the Laboratory for Specialized Clinical Studies, Cincinnati Children’s Hospital Medical Center in Cincinnati, Ohio, US, for testing by nested reverse transcription PCR assay and sequencing to identify G and P types.

### Statistical analysis

The primary endpoint was vaccine efficacy of 3 doses of vaccine versus placebo against a first episode of laboratory-confirmed SRVGE from 28 days after dose 3, as previously reported [11]. Assuming a 2% attack rate of SRVGE, a 50% true vaccine efficacy, and 20% participant non-assessibility, a total of 7,700 children were needed to detect a vaccine efficacy with a lower 95% confidence interval bound greater than 0% with at least 90% power. Under these assumptions, 117 cases of SRVGE (78 unvaccinated and 39 vaccinated) were required to fulfill the primary outcome. As an event-driven trial, the data was cut off for the primary efficacy analysis on 26 November 2015 when 117 cases of SRVGE occurring after 28 days after dose 3 were identified. At the time of the primary analysis, median age was 9.8 months. In the present paper, analyses of extended efficacy were undertaken for 3 periods among the same children: 28 days after dose 3 to 1 year of age, 1 to 2 years of age, and the combined period 28 days after dose 3 to 2 years of age. Secondary endpoints were efficacy against rotavirus gastroenteritis of any severity, very severe rotavirus gastroenteritis, and gastroenteritis of any cause. Efficacy by individual rotavirus G type, as well as vaccine type (G1, G2, G3, G4, and G9) and non-vaccine type (G8 and G12), was evaluated throughout the entire follow-up period against SRVGE, very severe rotavirus gastroenteritis, and rotavirus gastroenteritis of any severity. When more than 1 G type was isolated for an episode, the child was counted in every G type category for analysis of efficacy by G type.

The per-protocol population was considered the primary analysis population for vaccine efficacy and included children who received 3 doses of vaccine/placebo without major protocol violation and excluded participants with a laboratory-confirmed rotavirus episode from the time of first dose to 28 days after dose 3. Follow-up in the per-protocol population began 28 days after dose 3. For participants with more than 1 episode of SRVGE, only the first episode was counted towards the extended efficacy endpoint. Secondary analyses were done for the intention-to-treat population, which included all participants who were vaccinated with at least 1 dose of vaccine/placebo with follow-up beginning from the time of the first dose. Analyses of efficacy up to 2 years of age were undertaken among the same population contributing to the previously published primary analysis (August 2014 to November 2015; *N* = 3,508 per protocol; *N* = 4,091 intention to treat) triggered at accrual of the target number of primary endpoint cases. Description of the distribution of circulating rotavirus strains was made using the entire trial population (*N* = 6,567 children) from August 2015 to February 2018 to provide the broadest perspective with which to describe potential genotypic shifts over time.

Vaccine efficacy was calculated as (1 − IR_1_/IR_0_) × 100, where IR_1_ is the person-time incidence rate in the vaccinated group and IR_0_ is the person-time incidence rate in the placebo group. The incidence rate was calculated as the number of children reporting at least 1 event divided by the total follow-up time (calculated as the time to occurrence of the event, the date of dropout, or administrative censoring at the end of the follow-up period or at the time of first dose of study vaccine among placebo children following the primary analysis), with corresponding 95% confidence intervals derived from the exact confidence interval using the Poisson distribution. The number of events prevented (per 100 child-year) was calculated as 100 times the difference in the incidence rate of the placebo and vaccine groups; the associated confidence interval was derived using the method of Zou and Donner [14]. All *P* values were 2-sided with *P* < 0.05 considered statistically significant. Data analysis was conducted using SAS software (version 9.4, SAS Institute, Cary, North Carolina, US).

## Results

**Fig 1:**
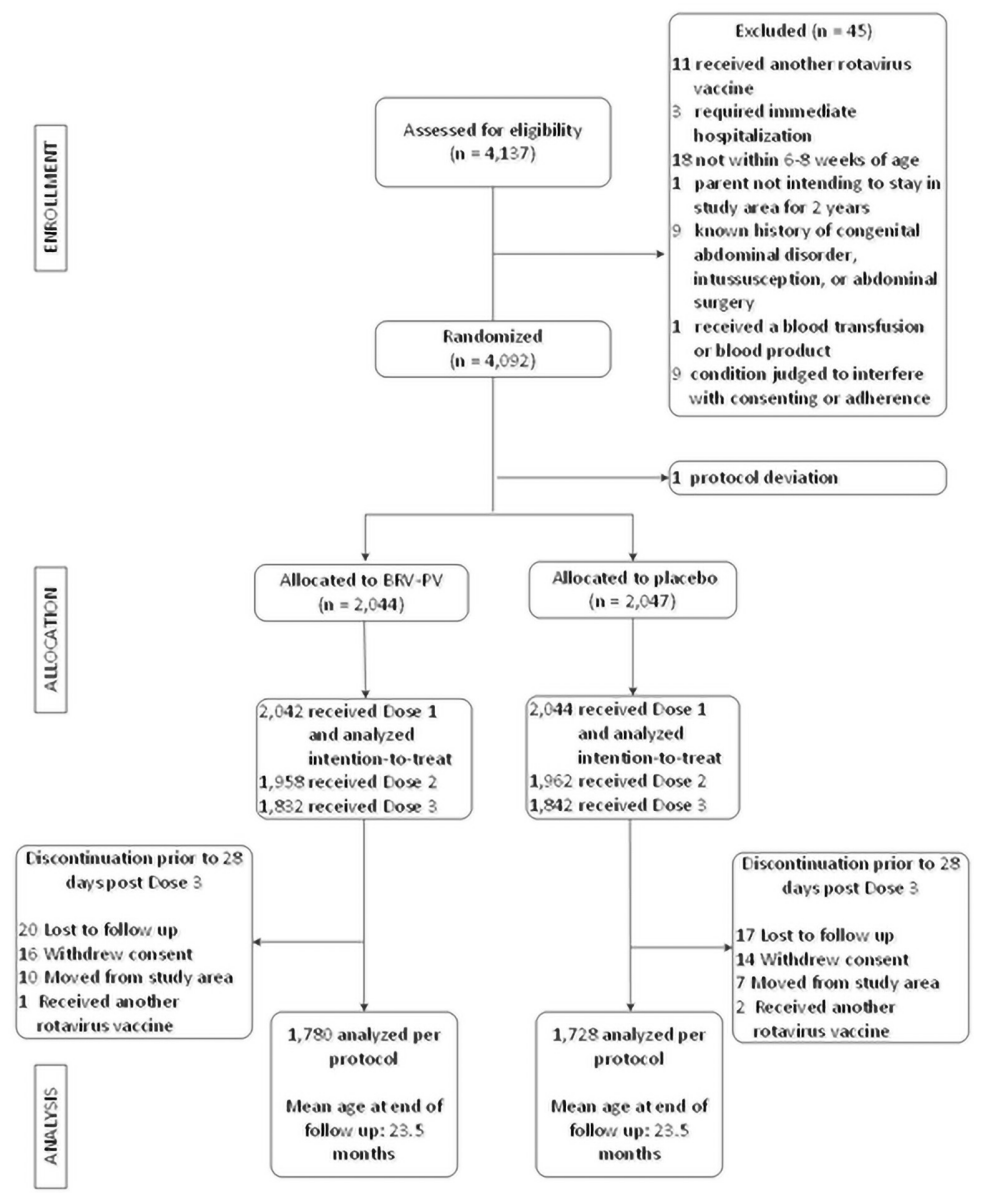
Flowchart of trial participants. BRV-PV, bovine rotavirus pentavalent vaccine.

From August 2014 to November 2015, a total of 4,137 infants were screened, and 4,091 (99%) were randomized and received at least 1 dose of vaccine or placebo (Fig 1). In total, 1,780 infants in the vaccine group and 1,728 infants in the placebo group received all 3 doses of study vaccine per protocol and were included in the per-protocol analysis of extended efficacy. Demographic characteristics were similar between groups (Table 1; Table A in S1 Table). Mean age at vaccination for the 3 study vaccine doses was 6.5, 10.5, and 14.5 weeks, and the mean age at the end of extended follow-up was 23.5 months, which did not differ by group.

**Table 1:** Characteristics of per-protocol participants.

Characteristic	Rotasiil	Placebo
*N*	1,780	1,728
Age in weeks, mean (SD)		
At dose 1	6.46 (0.65)	6.44 (0.63)
At dose 2	10.49 (0.70)	10.47 (0.69)
At dose 3	14.56 (0.79)	14.51 (0.77)
At end of extended efficacy follow-up	102.67 (10.14)	102.57 (11.21)
Male, *n* (%)	892 (50.11)	836 (48.38)
Weight (kg), mean (SD)	4.52 (0.72)	4.48 (0.66)
Length (cm), mean (SD)	54.34 (2.51)	54.35 (2.40)
OPV co-administered, *n* (%)		
At dose 1	724 (40.67)	685 (39.64)
At dose 2	799 (44.89)	773 (44.73)
At dose 3	809 (45.45)	765 (44.27)

OPV, oral poliovirus vaccine.

**Fig 2:**
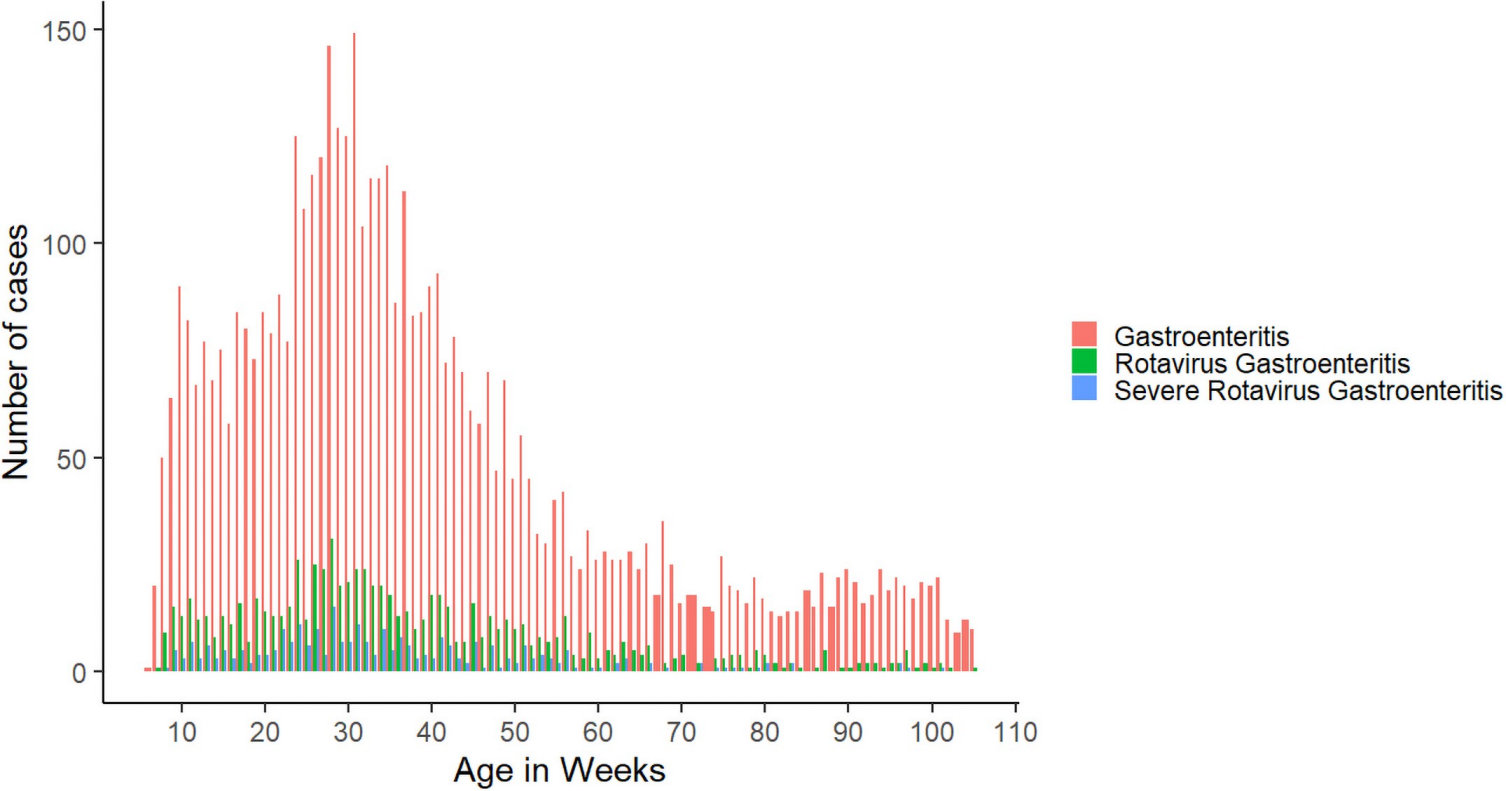
Distribution of gastroenteritis events by age.

The incidence of SRVGE peaked at 5 months of age, with 83.4% of first cases occurring before 12 months (Fig 2). Table 2 shows the incidence of rotavirus gastroenteritis by group in the first year of life, the second year of life, and the complete follow-up period up to 2 years of age in the per-protocol population. There were 44 children in the vaccine group and 105 children in the placebo group with SRVGE in the interval from 28 days after dose 3 to 1 year of age (3.96 cases versus 9.98 cases per 100 person-years, respectively), 15 children in the vaccine group and 17 children in the placebo group with SRVGE in the second year of life, and 59 children in the vaccine group and 122 children in the placebo group with SRVGE during the entire follow-up period up to 2 years of age, resulting in a per-protocol vaccine efficacy against SRVGE of 60.3% (95% CI 43.6% to 72.1%) in the first year of life, 17.8% (95% CI −64.6% to 58.9%) in the second year of life, and 54.7% (95% CI 38.1% to 66.8%) during the entire follow-up period up to 2 years of age. Up to 2 years of age, rotavirus vaccination prevented 2.56 episodes of SRVGE per 100 child-years. The vaccine did not provide significant protection against rotavirus gastroenteritis of any severity or gastroenteritis of any cause in any follow-up period. Similar efficacy was seen in the intention-to-treat analyses, with vaccine efficacy of 64.4% (95% CI 51.4% to 73.9%) in the first year of life, 25.7% (95% CI −46.2% to 62.2%) in the second year of life, and 60.4% (95% CI 47.6% to 70.1%) during the entire follow-up period up to 2 years of age (Table B in in S1 Table).

**Table 2:** Vaccine efficacy against gastroenteritis in the per-protocol population.

Outcome	Rotasiil (*N* = 1,780)			Placebo (*N* = 1,728)			Incidence rate difference, per 100 py (95% CI)	Vaccine efficacy (95% CI)
	Number with ≥1 episode	Person-years	Incidence rate per 100 py	Number with ≥1 episode	Person-years	Incidence rate per 100 py		
**Rotavirus gastroenteritis**								
<1 year								
All	176	1,065.22	16.52	252	1,002.41	25.14	−8.62 (−12.57 to −4.67)	34.3 (20.3 to 45.8)
Severe	44	1,111.59	3.96	105	1,052.20	9.98	−6.02 (−8.26 to −3.78)	60.3 (43.6 to 72.1)
Very severe	10	1,122.67	0.89	31	1,080.37	2.87	−1.98 (−3.13 to −0.83)	69.0 (36.7 to 84.8)
1 year to <2 years								
All	57	1,503.49	3.79	54	1,381.96	3.91	−0.12 (−1.55 to 1.32)	3.0 (−40.8 to 33.1)
Severe	15	1,661.22	0.90	17	1,547.81	1.10	−0.20 (−0.89 to 0.50)	17.8 (−64.6 to 58.9)
Very severe	2	1,705.13	0.12	1	1,631.34	0.06	0.06 (−0.15 to 0.26)	−91.3 (−2,010.2 to 82.7)
Total follow-up								
All	233	2,568.71	9.07	306	2,384.37	12.83	−3.76 (−5.61 to −1.91)	29.3 (16.2 to 40.4)
Severe	59	2,772.80	2.13	122	2,600.01	4.69	−2.56 (−3.56 to −1.57)	54.7 (38.1 to 66.8)
Very severe	12	2,827.79	0.42	32	2,711.71	1.18	−0.76 (−1.23 to −0.28)	64.0 (30.2 to 81.5)
**Gastroenteritis from any cause**								
<1 year								
All	834	789.38	105.65	835	760.16	109.84	−4.19 (−14.5 to 6.15)	3.8 (−5.9 to 12.6)
Severe	266	1,030.34	25.82	283	984.26	28.75	−2.94 (−7.50 to 1.63)	10.2 (−6.2 to 24.1)
Very severe	28	1,115.99	2.51	74	1,063.74	6.96	−4.45 (−6.29 to −2.61)	63.9 (44.3 to 76.7)
1 year to <2 years								
All	124	832.13	14.90	135	775.11	17.42	−2.52 (−6.45 to 1.42)	14.4 (−9.2 to 33.0)
Severe	45	1,427.15	3.15	50	1,355.07	3.69	−0.54 (−1.91 to 0.84)	14.5 (−27.8 to 42.9)
Very severe	11	1,682.38	0.65	6	1,589.55	0.38	0.28 (−0.21 to 0.77)	−73.2 (−368.4 to 35.9)
Total follow-up								
All	958	1,621.51	59.08	970	1,535.27	63.18	−4.10 (−9.56 to 1.36)	6.5 (−2.2 to 14.5)
Severe	311	2,457.49	12.66	333	2,339.34	14.23	−1.58 (−3.66 to 0.50)	11.1 (−3.8 to 23.8)
Very severe	39	2,798.37	1.39	80	2,653.29	3.02	−1.62 (−2.41 to −0.83)	53.8 (32.2 to 68.5)

py, person-years.

**Fig 3:**
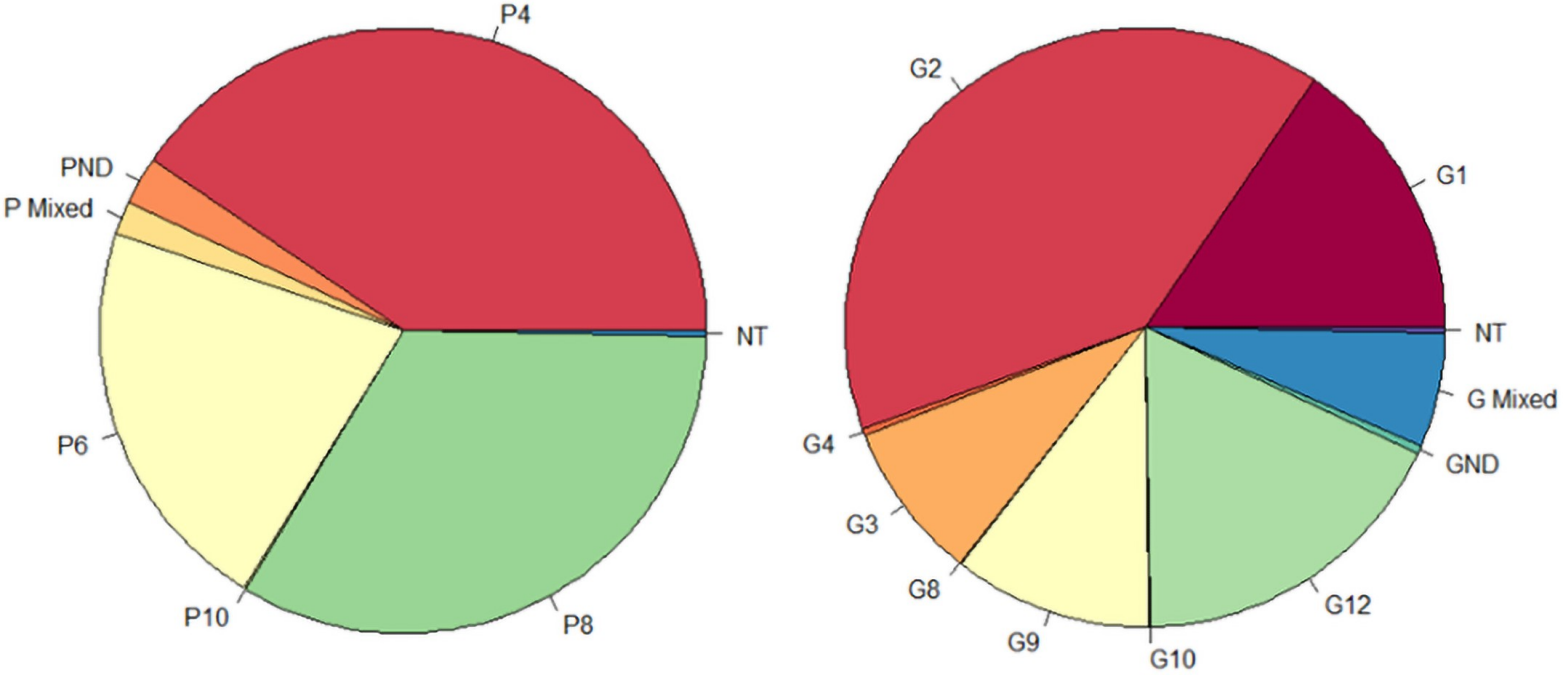
Prevalence of vaccine genotypes among rotavirus isolates in Madarounfa, Niger (August 2015 to February 2018). Rotasiil vaccine included G1, G2, G3, G4, and G9 types and P[5] type. GND, G type not determined; NT, non-typable; PND, P type not determined.

**Fig 4:**
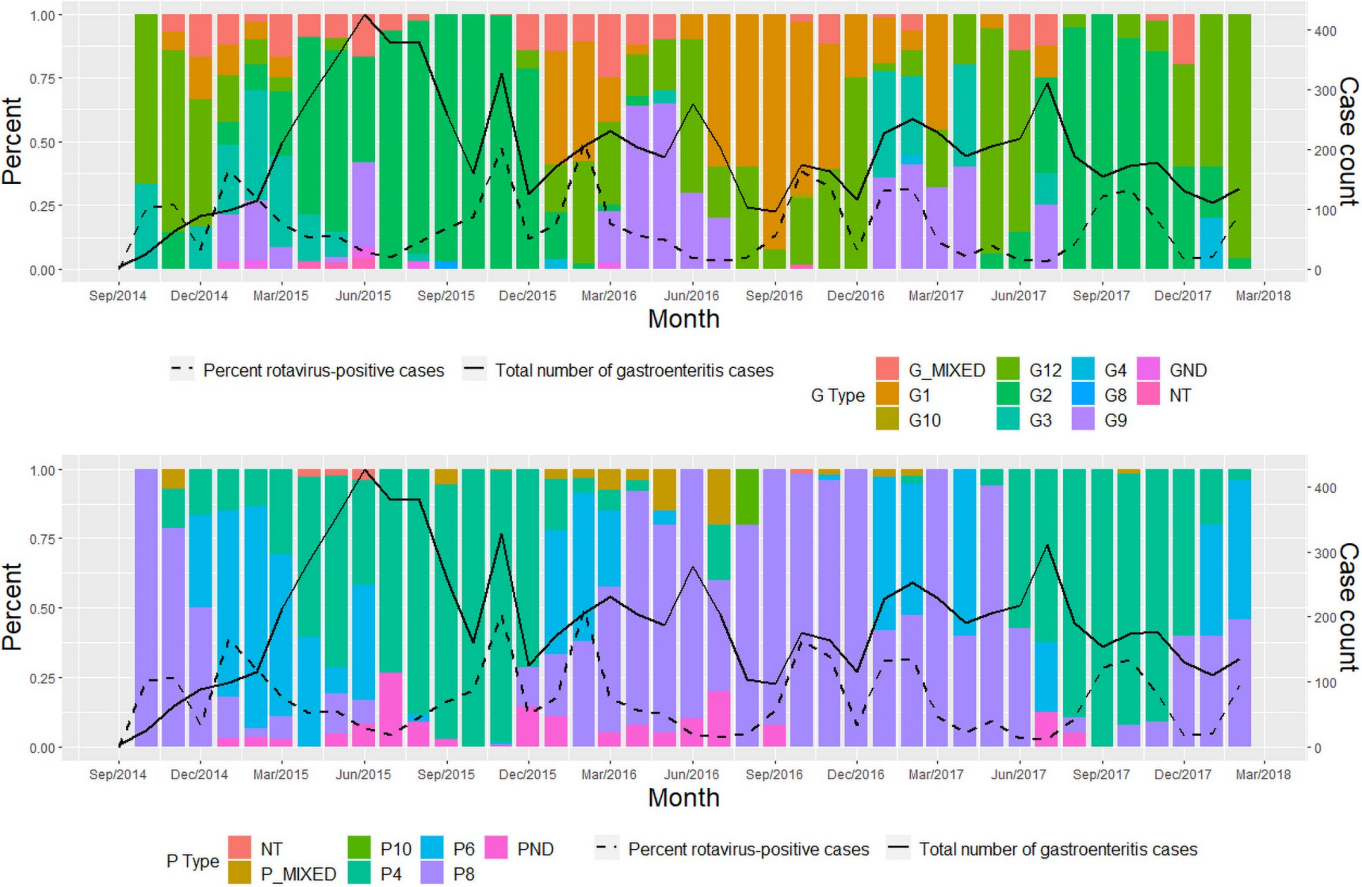
Burden of rotavirus gastroenteritis and circulating rotavirus strains by G type and P type from August 2015 to February 2018. G type (top panel); P type (bottom panel). GND, G type not determined; NT, non-typable; PND, P type not determined.

Circulating rotavirus strains from the entire trial population included 1,380 episodes of rotavirus gastroenteritis from August 2015 to February 2018. Rotaviruses with G types covered by the vaccine predominated during the study period (Fig 3; Table C in S1 Table). G2 was the most prevalent G type and constituted 37.8% of strains among all cases of rotavirus gastroenteritis, followed by genotypes G12 (18.9%) and G1 (15.7%). There was, however, a shift in G type predominance over time (Fig 4; Table D in S2 Table). G2 was the predominant G type during the first peak of the first rotavirus season (detected in 95.9% of stools tested from October to December 2015), whereas in the second peak of the first rotavirus season, G1 gained predominance and there was a marked increase in G12 and G9. In the second year of the study, G1 and G12 dominated the first rotavirus season, while G3 and G9 dominated the second rotavirus season.

Rotasiil provided significant protection against SRVGE caused by rotavirus serotypes contained in the vaccine (1.57 and 3.99 cases per 100 person-years for vaccine and placebo, respectively; vaccine efficacy 60.7%, 95% CI 44.1% to 72.3%), as well as rotavirus serotypes not contained in the vaccine (0.32 and 0.40 cases per 100 person-years for vaccine and placebo, respectively; vaccine efficacy 20.9%, 95% CI −90.9% to 67.2%; Table 3; Table E in S1 Table). By individual rotavirus genotype, estimates of efficacy against SRVGE were consistent with the overall protective efficacy.

**Table 3:** Strain-specific vaccine efficacy against rotavirus gastroenteritis in the per-protocol population, by severity.

Rotavirus gastroenteritis outcome by G type and P type	Rotasiil (*N* = 1,780)			Placebo (*N* = 1,728)			Incidence rate difference, per 100 py (95% CI)	Vaccine efficacy (95% CI)
	Number with ≥1 episode	Person-years	Incidence rate per 100 py	Number with ≥1 episode	Person-years	Incidence rate per 100 py		
**Vaccine G type (G1, G2, G3, G4, and G9)**								
Any VT								
All	179	2,643.28	6.77	253	2,457.57	10.29	−3.52 (−5.13 to −1.91)	34.2 (20.3 to 45.7)
Severe	44	2,805.50	1.57	105	2,633.76	3.99	−2.42 (−3.31 to −1.53)	60.7 (44.1 to 72.3)
Very severe	9	2,846.88	0.32	26	2,732.69	0.95	−0.64 (−1.06 to −0.22)	66.8 (29.1 to 84.4)
G1 alone								
All	29	2,833.37	1.02	46	2,721.79	1.69	−0.67 (−1.28 to −0.05)	39.4 (3.6 to 62.0)
Severe	4	2,854.29	0.14	14	2,751.26	0.51	−0.37 (−0.67 to −0.07)	72.5 (16.3 to 90.9)
Very severe	0	2,858.75	0	0	2,767.26	0	N/A	N/A
G2 alone								
All	113	2,708.38	4.17	169	2,542.25	6.65	−2.48 (−3.74 to −1.21)	37.2 (20.4 to 50.5)
Severe	29	2,819.89	1.03	69	2,676.37	2.58	−1.55 (−2.26 to −0.84)	60.1 (38.5 to 74.2)
Very severe	7	2,848.68	0.25	22	2,738.90	0.80	−0.56 (−0.94 to −0.18)	69.4 (28.4 to 86.9)
G3 alone								
All	10	2,846.51	0.35	14	2,748.80	0.51	−0.16 (−0.50 to 0.19)	31.0 (−55.3 to 69.4)
Severe	2	2,855.87	0.07	10	2,752.59	0.36	−0.29 (−0.54 to −0.05)	80.7 (12.0 to 95.8)
Very severe	1	2,857.42	0.03	2	2,764.11	0.07	−0.04 (−0.16 to 0.08)	51.6 (−433.4 to 95.6)
G4 alone								
All	1	2,857.30	0.03	0	2,767.26	0	0.03 (N/A to N/A)	0.00 (N/A to N/A)
Severe	0	2,858.75	0	0	2,767.26	0	N/A	N/A
Very severe	0	2,858.75	0	0	2,767.26	0	N/A	N/A
G9 alone								
All	33	2,829.89	1.17	29	2,741.66	1.06	0.11 (−0.45 to 0.66)	−10.3 (−81.6 to 33.1)
Severe	9	2,851.67	0.32	13	2,754.32	0.47	−0.16 (−0.49 to 0.17)	33.1 (−56.4 to 71.4)
Very severe	1	2,858.27	0.03	2	2,764.21	0.07	−0.04 (−0.16 to 0.08)	51.7 (−433.3 to 95.6)
Mixed VT								
All	6	2,850.05	0.21	9	2,757.31	0.33	−0.12 (−0.39 to 0.16)	35.5 (−81.2 to 77.0)
Severe	2	2,855.83	0.07	4	2,763.11	0.14	−0.07 (−0.25 to 0.09)	51.6 (−164.1 to 91.1)
Very severe	0	2,858.75	0	1	2,767.24	0.04	N/A	N/A
**Non-vaccine G type (G8 and G12)**								
G8 or G12								
All	33	2,824.84	1.17	35	2,728.27	1.28	−0.11 (−0.70 to 0.47)	8.9 (−46.5 to 43.4)
Severe	9	2,849.24	0.32	11	2,754.24	0.40	−0.09 (−0.40 to 0.23)	20.9 (−90.9 to 67.2)
Very severe	2	2,856.78	0.07	4	2,761.88	0.14	−0.07 (−0.25 to 0.10)	51.7 (−163.9 to 91.2)
G8 alone								
All	0	2,858.75	0	1	2,766.02	0.04	−0.04 (N/A to N/A)	100.0 (N/A to N/A)
Severe	0	2,858.75	0	1	2,766.02	0.04	−0.04 (N/A to N/A)	100.0 (N/A to N/A)
Very severe	0	2,858.75	0	0	2,767.26	0	N/A	N/A
G12 alone								
All	32	2,825.29	1.13	34	2,729.52	1.25	−0.11 (−0.69 to 0.46)	9.1 (−47.4 to 43.9)
Severe	9	2,849.24	0.32	10	2,755.48	0.36	−0.05 (−0.35 to 0.26)	13.0 (−114.2 to 64.6)
Very severe	2	2,856.78	0.07	4	2,761.88	0.14	−0.07 (−0.25 to 0.10)	51.7 (−163.9 to 91.2)
Mixed NVT								
All	12	2,845.36	0.42	13	2,751.37	0.47	−0.05 (−0.40 to 0.30)	10.7 (−95.6 to 59.3)
Severe	4	2,853.72	0.14	2	2,764.31	0.07	0.07 (−0.10 to 0.24)	−93.7 (−957.7 to 64.5)
Very severe	1	2,857.28	0.03	1	2,765.65	0.04	−0.001 (−0.10 to 0.10)	3.2 (−1447.5 to 94.0)
**P type**								
P[4]								
All	118	2,702.34	4.37	171	2,541.26	6.73	−2.36 (−3.64 to −1.08)	35.1 (18.0 to 48.7)
Severe	31	2,816.95	1.10	70	2,676.64	2.62	−1.52 (−2.24 to −0.79)	57.9 (35.8 to 72.4)
Very severe	8	2,847.22	0.28	22	2,738.90	0.80	−0.52 (−0.91 to −0.13)	65.0 (21.4 to 84.4)
P[6]								
All	41	2,809.17	1.46	61	2,696.06	2.26	−0.80 (−1.53 to −0.08)	35.5 (4.2 to 56.6)
Severe	6	2,851.04	0.21	31	2,728.01	1.14	−0.93 (−1.36 to −0.49)	81.5 (55.6 to 92.3)
Very severe	1	2,857.42	0.03	6	2,759.48	0.22	−0.18 (−0.37 to 0.005)	83.9 (−33.7 to 98.1)
P[8]								
All	62	2,806.71	2.21	70	2,699.81	2.59	−0.38 (−1.20 to 0.44)	14.8 (−19.9 to 39.5)
Severe	17	2,843.70	0.60	19	2,747.20	0.69	−0.09 (−0.52 to 0.33)	13.6 (−66.3 to 55.1)
Very severe	3	2,856.31	0.11	4	2,761.82	0.14	−0.04 (−0.22 to 0.15)	27.5 (−224.0 to 83.8)

N/A, not applicable; NVT, non-vaccine type; py, person-years; VT, vaccine type.

## Discussion

In our double-blind, placebo-controlled trial in Niger, Rotasiil provided protection against SRVGE in infants through an extended follow-up period of approximately 2 years. This protection was significant in the first year of life, when the disease burden and risk of death are highest, and against a changing pattern of rotavirus strains during the 2-year efficacy period. Our results highlight the early age of infection in this setting, supporting the benefit of ensuring complete vaccination early in life, but through the second year if doses are missed. As the number and diversity of available, licensed, and WHO-prequalified rotavirus vaccines increases [15], studies such as this provide valuable information for the planning of national immunization programs and future vaccine development.

Our estimate of efficacy against SRVGE during the first year of life is similar to that of other vaccines assessed in other high- and moderate-mortality countries (Fig A in S1 Table; Ghana, Kenya, and Mali: 64.2%, 95% CI 40.2% to 79.4%; Bangladesh and Vietnam: 51.0%, 95% CI 12.8%–73.3%; South Africa and Malawi: 61.2%, 95% CI 44.0% to 73.2% [16–18]). However, as reported elsewhere, this efficacy was substantially lower than that observed in clinical trials in low-mortality countries, where an efficacy of 85%–100% against SRVGE has been demonstrated [19–21]. The underlying mechanisms for this efficacy gap remain poorly understood [22], but the gap may be due to differences in rotavirus epidemiology (e.g., earlier age at first infection in low-resource settings, which confers natural protection in the placebo group) [23,24]; host characteristics (e.g., poor nutritional status, differences in the gut microbiome [25], enteropathy, and enteric coinfections); interference by maternal antibodies in breast milk [26]; or co-administration of OPV, which reduces rotavirus antibody levels [27–29]. A better understanding of the biological causes of reduced rotavirus vaccine efficacy in low-resource settings is needed to maximize the impact of rotavirus vaccines in the populations that are at highest risk for rotavirus morbidity and mortality, and additional studies are underway to elucidate how to improve the performance of live oral attenuated vaccines, including studies evaluating the influence of the microbiome and the effect of additional doses and improved sanitation [30].

We reported declines in vaccine efficacy beyond the first year of life, from 60.3% to 17.8%. This decline is in contrast to low-mortality countries, where vaccine efficacy was maintained into the second year of life [19,31–35], but consistent with clinical studies in high- and moderate-mortality settings, where vaccine efficacy was lower in the second year of life [18,36–38]. Vaccine efficacy was 77% and 40% during the first and second years of life, respectively, in South Africa [38], and 64.2% in the first year of life and 19.6% in the second year in pooled analyses from Ghana, Kenya, and Mali [18]. Several post-introduction effectiveness studies in resource-poor settings have also reported similar observations of a higher impact of the vaccine on hospitalizations in the first year of life [5,39].

While this study was not powered to measure vaccine efficacy in the second year of life, the lower efficacy observed in this period may be due to a number of factors, including high rotavirus incidence in this setting and waning immunity. Since exposure to natural rotavirus infection confers protection against the subsequent development of severe rotavirus disease [24], reduced efficacy in the second year of life could be partly explained by exposure of the placebo group to natural rotavirus infection, and acquired immunity, in the first year of life. Lower incidence and lower efficacy in the second year of life may be due to natural protection among the unvaccinated children due to repeated natural infection [30]. Epidemiological studies suggested that more than three-quarters of rotavirus symptomatic disease occurred in African infants before 12 months of age [40,41], but in a review of the placebo groups of clinical studies, significant exposure to wild-type rotavirus infection was demonstrated to be even earlier, with approximately 11%–13% of infants in Malawi and South Africa exposed to natural infection by 6–8 weeks of age and 25% by 20–24 weeks of age [30,42]. In our study setting, 38% of the placebo group had serological evidence of exposure to natural rotavirus infection at approximately 18 weeks of age [43], suggesting a high baseline incidence and exposure to rotavirus infection early in life. These results are in contrast to studies from Europe that demonstrate a lower incidence [44] and later age of first SRVGE episode, with 40% of infection occurring in children 12–23 months of age [45]. Early immunization (e.g., birth or neonatal administration before exposure to the first symptomatic natural infection) may be considered as a possible strategy to maximize the impact of rotavirus vaccines in settings where the burden of rotavirus gastroenteritis is high in the first 6 months of life [46].

A second possible explanation for lower efficacy over the second year may be related to waning immunity. Children in high- and moderate-mortality settings had lower geometric mean titers of antirotavirus antibodies after vaccination than children in low-mortality countries [18,47–50]. Lower antibody titers after vaccination might wane sooner than higher levels, resulting in lower protection in the second year. Booster doses may be considered to counteract waning immunity.

In our study, we detected a wide variety of rotavirus genotypes circulating over 4 rotavirus seasons. Only 15.7% of the rotavirus strains in this setting were G1, the most commonly occurring strain globally [51]. In addition to the common G1, we observed 7 other G types (G2, G3, G4, G8, G9, G10, and G12) and 4 P types (P[4], P[6], P[8], and P[10]) in circulation; 74.7% of cases were vaccine type. Strain diversity has been shown to be high in Africa, where G1 and G2 strains have dominated but G8 and G9 have emerged [52–54]. Importantly, strain diversity can be cyclical in human populations, with dominant strains emerging every 3–4 years [55,56], but strains are known to have important geographical differences and to evolve over time with natural molecular evolution [57,58]. The second year of this study demonstrated a shift in strain predominance from G2 to G1, as well as a large increase in G9 and G3.

The wide circulation of diverse rotavirus strains in the region raises the question of whether protective immunity is homotypic (same G or P type) or heterotypic (different G or P type) [59] and underscores the importance of demonstrating cross-protective efficacy of the rotavirus vaccines in preventing severe gastroenteritis. Given the diversity of the rotavirus types in circulation and the global emergence of new strains in the human population, homotypic protection alone would be unlikely to provide complete protection against SRVGE. Here, Rotasiil efficacy was demonstrated against individual rotavirus genotypes contained and not contained in the vaccine, suggesting significant homotypic and heterotypic protection against SRVGE. These data are consistent with other clinical trials that have demonstrated heterotypic protection of rotavirus vaccines against multiple rotavirus strains [17,60,61] and suggest that rotavirus diversity per se may not be a critical challenge for vaccine performance. The heterotypic protection afforded by Rotasiil suggests it may be effectively used throughout sub-Saharan Africa and in other regions of the world. The potential for widespread use of rotavirus vaccines to result in evolutionary selective pressure resulting in strain replacement [62,63]—and subsequent impacts on vaccine performance, as was seen after the introduction of pneumococcal vaccine—can be assessed through continued monitoring to ensure that rotavirus vaccines continue overall to provide an important public health impact in reducing disease and virus transmission.

Our study was designed to include a population broadly representative of those in sub-Saharan Africa, with poor socioeconomic conditions and high mortality from diarrhea. Our study setting was notably characterized by a high incidence of rotavirus disease, high exposure to natural rotavirus infection early in infancy, and a wide diversity of circulating rotavirus strains. The delivery of rotavirus vaccines in routine childhood immunization schedules in settings where the burden of rotavirus mortality is highest will have a profound public health impact. Current guidance emphasizes the importance of complete vaccination with any rotavirus vaccine to prevent childhood mortality and morbidity [64]. National cost-effectiveness analyses can help make the case for vaccine delivery and provide compelling evidence to guide policy in resource-limited settings [65].

This study has several limitations. First, study vaccine was not consistently given concomitantly with OPV. Extrapolation to settings with concomitant administration of OPV may be limited; however, secondary analysis estimating vaccine efficacy by OPV vaccine administration status suggested that the high efficacy observed in this study is not due to lower rates of concomitant administration [11]. Second, the Vesikari score was originally designed for use in settings of high parental literacy, which may have led to underscoring of some cases, although this would not differ between groups. Third, the study had limited power to assess efficacy in the second year of life owing to the low number of observed cases. Finally, the study was unblinded following the primary analysis to allow placebo children to receive the study vaccine, and follow-up among children initially randomized to placebo was censored at the time of receipt of study vaccine to allow for comparison of vaccine efficacy. The loss of follow-up may have contributed to further bias, but the magnitude is expected to be limited given the small number of children affected and the older age at the time of vaccine receipt compared to the early age of first infection.

### Conclusion

We showed that Rotasiil, a heat-stable, affordable oral rotavirus vaccine, offered substantial protection against SRVGE through 2 years of life and across a wide diversity of strains—confirming that the potential public health impact of introducing rotavirus vaccines in Niger can be substantial. Vaccines that are safe, effective, and protective against multiple strains represent the best hope for preventing the severe consequences of rotavirus infection, especially in resource-limited settings, where access to care may be limited. Delivery of this vaccine in national childhood immunization schedules can be expected to greatly reduce the burden of rotavirus disease.

## Supporting information

S1 CONSORT Checklist CONSORT checklist. (PDF)

S1 Protocol Study protocol. (PDF)

S1 Statistical Analysis Plan Statistical analysis plan. (PDF)

S1 Table Supplemental results including Table A, Table B, Table C, Table E, and Fig A. (DOCX)

S2 Table Supplemental results including Table D. (XLSX)

## Abbreviations

DSMB: data and safety monitoring board
OPV: oral poliovirus vaccine
SRVGE: severe rotavirus gastroenteritis
