# Supplementary material for: Rotavirus vaccine efficacy up to 2 years of age and against diverse circulating rotavirus strains in Niger: Extended follow-up of a randomized controlled trial
Source: PLoS Med. 2021 Jul 2;18(7):e1003655. doi: 10.1371/journal.pmed.1003655 (PMC8253401; doi:10.1371/journal.pmed.1003655)
Supplement: S1 Protocol — (PDF) [file pmed.1003655.s002.pdf]

Randomized, double-blind, placebo-controlled phase III clinical trial to assess the efficacy and safety of a pentavalent rotavirus vaccine (BRV-PV) against severe rotavirus gastroenteritis among infants in Niger

Study Protocol

Version 6.5

June 6, 2015

**Title**	Randomized, double-blind, placebo-controlled phase III clinical trial to assess the efficacy and safety of a pentavalent rotavirus vaccine (BRV-PV) against severe rotavirus gastroenteritis among infants in Niger
**Study Site**	Madarounfa, Niger
**Primary Sponsor**	Epicentre<br>8 rue Saint Sabin<br>Paris 75011, France
**Secondary Sponsor**	Médecins Sans Frontières-Operational Center Geneva<br>78 rue de Lausanne<br>CP 116<br>1211 Geneva 21, Switzerland
**Sponsor Study Director**	Rebecca F. Grais, PhD<br>Epicentre<br>8 rue Saint Sabin<br>Paris 75011, France<br>Tel : + 33 1 4021 5475<br>Email : rebecca.grais@epicentre.msf.org
**Sponsor Principal Investigator**	Sheila Isanaka, ScD<br>Epicentre<br>8 rue Saint Sabin<br>Paris 75011, France<br>Tel : + 33 1 4021 5498<br>Email : sheila.isanaka@epicentre.msf.org
**Site Principal Investigator**	Ousmane Guindo, MD<br>Epicentre Niger<br>Tel : +277 9944 3721<br>Email: ousmane.guindo@epicentre.msf.org
**Medical Monitor**	Lynda Woï Messé-Touré, MD<br>Epicentre Niger<br>Tel : +277 9780 0444 / 9036 6934<br>Email: lynda.woi-messe@epicentre.msf.org
**Sponsor Co-investigators**	Emmanuel Baron, MD<br>Epicentre<br>Tel : +33 1 4021 5479

	Email : emmanuel.baron@epicentre.msf.org<br><br>Céline Langendorf, PhD<br>Epicentre<br>Tel : + 33 1 4021 5512<br>Email : celine.langendorf@epicentre.msf.org
**Study Statistician**	Brian D. Plikaytis, MSc<br>BioStat Consulting, LLC<br>2739 Livsey Trail<br>Tucker, GA 30084<br>Tel : + 1 770-939-6968 / 770-328-7349<br>Email: bdplikaytis@gmail.com
**Operational Partners**	Health District of Madarounfa, Maradi, Niger<br>Médecins Sans Frontières-Operational Center Paris<br>Forum Santé Niger
**Participating Laboratories**	Aimé Makimere<br>Epicentre Maradi Laboratory<br>Boite Postal 419<br>Maradi, Niger<br>Tel: + 227 2041 0282<br>Email : aime.makimere@epicentre.msf.org<br><br>Monica Malone McNeal, MSc<br>Cincinnati Children's Hospital Medical Center<br>Division of Infectious Diseases<br>LSCS Associate Director<br>3333 Burnet Ave ML6014<br>Cincinnati, OH 45229-3039<br>Tel: 1-513-636-7648<br>Email: monica.mcneal@cchmc.org
**Randomization**	Varsha Parulekar<br>DiagnoSearch Life Sciences Pvt. Ltd<br>702, Dosti Pinnacle, E-7, Road 22,<br>Wagle Estate, Thane 400604<br>India<br>Tel: +91 22 6777 6300<br>Email: varsha.parulekar@diagnosearch.com

**Protocol Version**	Version 6.5 (Issue date: June 6, 2015)
**Revision Chronology (Date and Reason for Amendment)**	September 8, 2013: Protocol Version 6<br>March 14, 2014: Amendment 1 Version 6.2<br>September 12, 2014: Amendment 2 Version 6.4<br>June 6, 2015: Amendment 3, Version 6.5

PROTOCOL APPROVAL PAGE

We, the undersigned, have read and understood this protocol and other appropriate related documents, including the Investigator's Brochure for rotavirus vaccine. We hereby agree to conduct the study in accordance with this protocol and to comply with all requirements regarding the obligations of investigators, the ethical principles that have their origin in the Declaration of Helsinki and all other pertinent requirements of the ICH HARMONISED TRIPARTITE GUIDELINE, GUIDELINE FOR GOOD CLINICAL PRACTICE E6 (R1) and applicable regulatory authority.

We agree to comply with all relevant SOPs required for the conduct of this study. We further agree to ensure that all associates assisting in the conduct of this study are informed regarding their obligations.

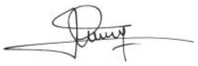

________________________________ 5 June 2015

Dr. Ousmane Guindo Date
Site Principal Investigator
Epicentre Niger
Tel : +277 9944 3721

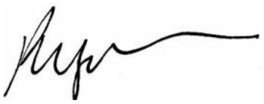

________________________________ 6 June 2015

Sponsor Study Director Date
Dr. Rebecca Freeman Grais
Epicentre, 8 rue Saint Sabin, 75011 Paris France
Email: rebecca.grais@epicentre.msf.org
Tel: +33 (0)1 4021 5475

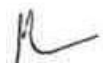

________________________________ 5 June 2015

Secondary Sponsor's Representative: Date
Bruno Jochum
Médecins Sans Frontières-Operational Center Geneva
Email: bruno.jochum@geneva.msf.org
Tel: + 41 22 849 8484

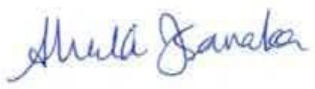

______________________________________	6 June 2015
Primary Sponsor's Representative:	Date

Sheila Isanaka
Epicentre, 8 rue Saint Sabin, 75011 Paris France
Email: sheila.isanaka@epicentre.msf.org
Tel: +33 (0)1 4021 5475

TRIAL REGISTRATION DATA

Primary Registry and Trial Identifying Number: ClinicalTrials.gov (ClinicalTrials.gov Identifier: NCT02145000)

Date of Registration in Primary Registry: May 20, 2014

Secondary Identifying Numbers: None

Sources of Monetary and Material Support
Médecins Sans Frontières-Operational Center Geneva will provide funding for the trial. Vaccine and placebo are to be provided in-kind by the Serum Institute of India, Limited.

Primary Sponsor
Epicentre takes responsibility for initiating, registering and conducting the trial, and as such, will be involved in the study design; collection, management and analysis, and interpretation of data; and writing of the report. Epicentre takes responsibility for ensuring the trial is properly monitored and results are made available.

Primary sponsor contact: Dr. Rebecca Freeman Grais, Director, Department of Epidemiology and Population Health, Epicentre (rebecca.grais@epicentre.msf.org; Tel: +33 (0)1 4021 5475; Address: Epicentre, 8 rue Saint Sabin, 75011 Paris France)

Secondary Sponsor
Médecins Sans Frontières- Operational Center Geneva has agreed with the primary sponsor to act as the secondary sponsor and the primary sponsor's legal representative in relation to the trial site and provide funding for the trial. The secondary sponsor will be involved in the study design, interpretation of data and writing of the report.

Secondary sponsor contact: Dr. Bruno Jochum (General Director, Médecins Sans Frontières-Operational Center Geneva; Address: 78 rue de Lausanne, CP 116, 1211 Geneva 21, Switzerland; Tel: + 41 22 849 84 84; Email: bruno.jochum@geneva.msf.org)

Contact for Public Queries
The following individuals will provide responses to general queries including information about current recruitment status:

- Dr. Ousmane Guindo (Site Principal Investigator, Epicentre Nige; Address : BP 13330 Niamey Niger; Tel : +277 9944 3721; Email : ousmane.guindo@epicentre.msf.org)

Contact for Scientific Queries

- Dr. Rebecca Freeman Grais, Sponsor Study Director, Department of Epidemiology and Population Health, Epicentre (rebecca.grais@epicentre.msf.org; Tel: +33 (0)1 4021 5475; Epicentre, 8 rue Saint Sabin, 75011 Paris France)
- Dr. Sheila Isanaka, Sponsor Principal Investigator, Epicentre (sheila.isanaka@epicentre.msf.org; Tel: +33 (0)1 4021 5498; Epicentre, 8 rue Saint Sabin, 75011 Paris France)

Public Title

Efficacy and safety of a pentavalent rotavirus vaccine (BRV-PV) against severe rotavirus gastroenteritis in Niger

Scientific Title

Randomized, double-blind, placebo-controlled phase III clinical trial to assess the efficacy and safety of a pentavalent rotavirus vaccine (BRV-PV) against severe rotavirus gastroenteritis among infants in Niger

Country of Recruitment

Niger

Health Condition(s) or Problem(s) Studied

Severe rotavirus gastroenteritis

Interventions

Active comparator: Live attenuated bovine-human [UK] reassortant rotavirus vaccine manufactured by the Serum Institute of India, Limited (SIIL). The pentavalent vaccine (BRV-PV) contains rotavirus serotypes G1, G2, G3, G4, and G9 (≥5.6 log10 FFU/serotype/dose). The vaccine is in lyophilized form and supplied with 2.5 ml of citrate bicarbonate buffer that is added for reconstitution just before oral administration.

Placebo comparator: Same constituents as the active vaccine but without the viral antigens; manufactured by SIIL.

Key Inclusion and Exclusion Criteria

The study will be performed in infants in Madarounfa, Niger. Healthy male and female infants meeting the following inclusion criteria are eligible for enrollment:

(1) aged 6-8 weeks at the time of inclusion
(2) able to swallow and no history of vomiting within 24 hours
(3) resident in Madarounfa Health District and within the catchment area of the study health facilities
(4) intending to remain in the study area for 2 years
(5) parent/guardian providing written informed consent

Any of the following will exclude an infant from randomization in the study:

(1) known history of congenital abdominal disorders, intussusception, or abdominal surgery
(2) receipt of intramuscular, oral, or intravenous corticosteroid treatment within 2 weeks
(3) receipt or planned administration of a blood transfusion or blood products, including immunoglobulins
(4) any known immunodeficiency condition
(5) any serious medical condition
(6) any other condition in which, in the judgment of the investigator, would interfere with or serves as a contraindication to protocol adherence or the parent/guardian's ability to give informed consent

NOTE: There will be no restriction at the time of inclusion based on breast feeding around the time of vaccination, receipt of routine pediatric vaccinations, prematurity, low birth weight or HIV status. Need for immediate hospitalization, inability to swallow and history of vomiting within the last 24 hours of enrollment are the only conditions based on the infant's immediate clinical status to delay oral administration of the study vaccine or placebo.

Study Type
Interventional, individually randomized, double (e.g. investigator and participant) blinded, parallel two-arm, phase III event-driven trial to assess the efficacy and safety of a pentavalent rotavirus vaccine (BRV-PV) against severe rotavirus gastroenteritis when administered within the Expanded Programme on Immunization (EPI) of Niger. The trial contains a nested sub-study ("immunogenicity sub-cohort") to assess the immunogenicity of the BRV-PV vaccine and the effect of prenatal nutritional supplementation on infant immune response to the BRV-PV vaccine. Potential interference of the study vaccine with immunogenicity of EPI vaccines will also be explored.

Study design

The study is designed as a double-blinded, randomized, placebo-controlled, end-point driven trial with two groups of infants receiving vaccine or placebo (1:1 allocation) to assess the efficacy and safety of BRV-PV. Three doses of BRV-PV containing ≥ Log10 5.6 FFU/Dose of each serotype G1, G2, G3, G4 and G9 will be administered at 4 week intervals between doses (with a window of –1 to +4 weeks). The first administration will occur at 6-8 weeks of age.

Active surveillance for gastroenteritis episodes will be conducted throughout the trial. Surveillance for adverse events will be carried out among all children from the time of first vaccination until 28 days post-Dose 3. Surveillance for all serious adverse events, including intussusception and death, will be conducted on all participants until they each reach two years of age.

Infants will be individually randomized in a 1:1 ratio to receive three doses of the vaccine or placebo administered orally. The initial dose will be at 6-8 weeks (42-56 days) of age. Each subsequent dose will be administered after a 4-week interval (-1 to +4 weeks). Immunogenicity of the BRV-PV vaccine will be assessed in the immunogenicity sub-cohort at the time of the first dose and 28 days Post-Dose 3. To assess the effect of prenatal nutrition supplementation on infant immune response to the BRV-PV vaccine, study villages in the immunogenicity sub-cohort will be randomized in a 1:1:1 ratio to provide pregnant women with daily iron-folate, multiple micronutrients or a lipid-based nutrition supplement. Infants of participating women, if eligible at 6-8 weeks of age, will be randomized in a 1:1 ratio to receive three doses of vaccine or placebo and enter the main trial as part of the immunogenicity sub-cohort.

Unique identification numbers will be allocated by the Contract Research Organization (CRO) using a computer-generated random number list using permuted blocks of random sizes. Block sizes will not be disclosed to reduce predictability of the random sequence and ensure allocation concealment. Study physicians who oversee randomization will be given a subset of sequentially numbered silver coated booklets prepared by the CRO. The study physician will be instructed to assign the next sequential randomization code noted in the booklet to each eligible infant as (s)he is enrolled.

Vaccine and placebo packages will be labeled with an assigned code and delivered to the study site in otherwise identical presentations. Group assignment will remain concealed from study personnel, investigators and caregivers of participating infants for the whole study period. The Data and Safety Monitoring Board (DSMB) and a sponsor statistician not involved in the rest of the trial will also be masked to the group assignment. The DSMB will remain masked unless otherwise deemed necessary by the DSMB members for any safety related issues. Investigators conducting the final analysis will remain masked to the group assignment until the end of the analysis.

The study code will be broken only in case of a medical event in which the Site Principal Investigator/Medical Monitor deems the participant cannot be appropriately treated without knowing his/her group assignment. A booklet with group assignment will be securely held at the field site with the Medical Monitor. Any such case will be fully documented by the Site Principal Investigator/Medical Monitor and written notification will be provided to the sponsor within 48 hours.

Tentative Date of First Enrollment
June 2014

Target Sample Size
Assuming a 2% attack rate of severe rotavirus gastroenteritis, a 50% true vaccine efficacy and a 20% participant non-assessibility (including withdrawal and loss to follow up), the study will enroll 3885 children per group (total n = 7770) to have at least 90% power to detect a vaccine efficacy with a lower 95% confidence interval bound greater than 0%. Under these assumptions, a sample size of 7770 participants will result in 117 cases of severe rotavirus

gastroenteritis (78 unvaccinated and 39 vaccinated) required to fulfill the primary study objective and trigger analysis.

Assuming a sero-conversion rate of 30% in the placebo group, 20% non-assessibility (including withdrawal and loss to follow up) and 30% exclusion due to detection of rotavirus disease between vaccine doses, we will assess immunogenicity in 660 children per group to have at least 90% power to detect a 20% difference in the proportion of children that sero-convert.

Recruitment Status
Recruiting: participants are being recruited.

Study objectives and endpoints

Primary objective:

To estimate the efficacy of three doses of SIIL BRV-PV vaccine vs. placebo against severe rotavirus gastroenteritis in healthy infants in Niger.

Secondary objectives:

- To estimate the efficacy of the SIIL BRV-PV vaccine vs. placebo against severe rotavirus gastroenteritis from 28 days post-Dose 3 to 1 year of age and from 1 to 2 years of age, from 28 days post-Dose 3 to 2 years of age.
- To evaluate vaccine efficacy against rotavirus gastroenteritis of any severity.
- To estimate vaccine efficacy against rotavirus gastroenteritis with a Vesikari score ≥ 17.
- To estimate vaccine efficacy against gastroenteritis of any cause.
- To estimate vaccine efficacy against severe rotavirus gastroenteritis caused by G serotypes included in the vaccine (G1, G2, G3, G4 and G9).
- To estimate longitudinal prevalence of rotavirus gastroenteritis.
- To estimate vaccine efficacy to reduce hospitalizations due to rotavirus gastroenteritis.
- To estimate vaccine efficacy to reduce hospitalization for any reason.
- To estimate safety of the SIIL BRV-PV vaccine vs. placebo (adverse events [AEs] and serious adverse events [SAEs]).
- To estimate the immunogenicity of BRV PV in a sub-sample of participants.
- To estimate effect of prenatal nutritional supplementation on infant immune response to the vaccine in a sub-sample of participants.
- To demonstrate the immunological non-inferiority of EPI vaccines when co-administered with the BRV-PV as compared to their co-administration with placebo.

Primary endpoint:

The primary endpoint is vaccine efficacy of three doses of the SIIL BRV-PV vaccine vs. placebo against a first episode of laboratory confirmed severe rotavirus gastroenteritis from 28 days

post-Dose 3 until 117 cases are accrued or when all participating infants reach 2 years of age if 117 cases are not attained.

Secondary endpoints:

Efficacy:

- Laboratory-confirmed cases of severe rotavirus gastroenteritis from 28 days post-Dose 3 to 1 year of age and from 1 to 2 years of age, from 28 days post-Dose 3 to 2 years of age
- Laboratory-confirmed episodes of rotavirus gastroenteritis of any severity
- Laboratory-confirmed episodes of rotavirus gastroenteritis with a Vesikari score of ≥ 17
- Laboratory-confirmed cases of severe rotavirus gastroenteritis due to rotavirus serotypes G1, G2, G3, G4 and G9, i.e., the serotypes included in the vaccine
- Episodes of gastroenteritis of any cause
- Longitudinal prevalence of laboratory-confirmed rotavirus gastroenteritis
- Hospitalization due to laboratory-confirmed cases of rotavirus gastroenteritis of any cause
- Hospitalization of any cause

Safety:

- Adverse events from the time of Dose 1 to 28 days post-Dose 3
- SAEs in all participants through two years of age

Immunogenicity

- Anti-rotavirus IgA seroresponse rates and geometric mean titres in a subset of 1320 infants

Immune interference with EPI vaccines:

- Neutralizing antibody titers to polio virus serotypes 1, 2 and 3 ≥ 1:8 (seroprotection)
- Antibody concentrations to Diphtheria toxoid ≥ 0.1 IU/mL (seroprotection)
- Antibody concentrations to Tetanus toxoid ≥ 0.1 IU/mL (seroprotection)
- Antibody concentrations to Pertussis expressed as GMCs
- Antibody concentrations to Hepatitis B (anti-HBs) ≥ 10 mIU/mL (seroprotection)
- Antibody concentrations to Hib (anti-PRP antibodies) ≥ 1 mcg/mL (long term seroprotection)

Data Safety and Monitoring Board

An independent Data and Safety Monitoring Board (DSMB) has been established prior to initiating the study and is composed of 5 experts in operational, medical, and biostatistical aspects of clinical trials, including Dr. Jacqueline Deen, Chair (Menzies School of Public Health), Dr. Irène Adehossi (Ministry of Health Niger), Dr. Nael Lapidus (Université Paris VI)), Dr. Milagritos D. Tapia (University of Maryland), and Dr. Hamadou Ousseini Adamou (World Health Organization). All members of the DSMB will be completely uninvolved in the running of the trial and cannot be unfairly influenced by people or institutions involved in the trial. An initial safety review is planned after the first 1000 infants complete the 28-day post-Dose 3 period, with subsequent reviews every 6 months thereafter. Each meeting will include an administrative review to assess accrual, retention, and the progress of the study, as well as quarterly safety reviews including any serious adverse events.

Table A. Schema of overall study design

WEEK OF AGE		W0	W6	W10	W14	W18	W24	W36	W52	W64	W76	W88	W104
**Study Visit**	Pregnancy	Pre - random-ization	Random-ization and Dose 1	Dose 2	Dose 3	Dose 3 + 28 days	6 months	9 months	12 months	15 months	18 months	21 months	24 months
**ALL PARTICIPANTS**													
Home Visit*			⟶										
Scheduled Facility Visit			X	X	X	X	X	X	X		X		X
Surveillance													
Gastroenteritis and SAE			X	X	X	X	X	X	X	X	X	X	X
AE**			X	X	X	X							
Laboratory Assessment													
Child stool***			X	X	X	X	X	X	X	X	X	X	X
**IMMUNOGENCITY SUB-COHORT**													
Home Visit*	⟶												
Laboratory Assessment													
Child stool***			X	X	X	X	X	X	X	X	X	X	X
Child blood			X			X			X				X
Child urine			X	X	X	X	X	X	X	X	X	X	X
Maternal blood	X	X	X				X						
Maternal stool	X	X	X				X						
Maternal urine	X	X											
Breast milk		X	X				X						

* Home Visits scheduled for Pre-randomization and on a weekly basis until 2 years of age. In the immunogenicity sub-cohort, home visits will be conducted among all consenting women of reproductive age and continue until the child is 2 years of age.

** Surveillance for adverse events from the time of Dose 1 until 28 days post-Dose 3.

*** Stool collected from all participants for any case of gastroenteritis identified at facility or home within a recall period of 7 days until 2 years of age. In the immunogenicity sub-cohort, stool samples will be collected independent of gastroenteritis status at the time of each Dose, 28 days post-Dose 3, 6 months of age and every 3 months thereafter until 2 years of age.

ABBREVIATIONS

AE	Adverse Event
BRV-PV	Bovine-human reassortant rotavirus vaccine-pentavalent
CRF	Case Report Form
CRO	Contract Research Organization
DHS	Demographic and Health Survey
DSMB	Data and Safety Monitoring Board
EPI	Expanded Programme on Immunization
FORSANI	Forum Santé Niger
GAVI	Global Alliance for Vaccines and Immunization
GEMS	Global Enteric Multicenter Study
HIV	Human Immunodeficiency Virus
ICH	International Conference on Harmonization
GCP	Good Clinical Practice
IMCI	Integrated Management of Childhood Illness
ITT	Intention To Treat
MEM	Minimum Essential Medium
MSF	Médecins Sans Frontières
NGO	Non-governmental organization
ORS	Oral rehydration salts
PATH	Program for Appropriate Technology in Health
PI	Principal Investigator
ROC	Receiver Operating Characteristic
SAE	Serious adverse event
SIIL	Serum Institute of India, Limited
SOP	Standard Operating Procedure
UN	United Nations
UNICEF	United Nations Children's Fund
WHO	World Health Organization

TABLE OF CONTENTS

PROTOCOL APPROVAL PAGE 5

TRIAL REGISTRATION DATA 7

ABBREVIATIONS 15

TABLE OF CONTENTS 16

BACKGROUND AND RATIONALE 19

Epidemiology of childhood diarrhea 19

Current strategies for the management of childhood diarrhea 19

Epidemiology of rotavirus and public health response 20

Rotavirus in Niger 21

Rotavirus Vaccine of Serum Institute of India, Limited 22

Clinical evaluation of BRV-PV vaccine 22

Study rationale 24

STUDY OBJECTIVES AND ENDPOINTS 26

METHODOLOGY 28

Study design 28

Study setting 31

Investigational product (vaccine and placebo) 32

Choice of placebo comparator 34

Randomization procedures 35

Blinding procedures 36

Target population 36

Study sites 37

Study procedures 38

Recruitment and screening 38

Randomization and inclusion 39

Vaccination 40

Assessment of compliance 41

Immediate post-vaccination safety assessment 41

Follow up 41

Gastroenteritis definition and surveillance 43

Adverse event (AE) definition and surveillance 44
Discontinuation criteria 45
Immunogenicity sub-cohort 46
Laboratory assessment 48
Participant retention 50
Standard care 50
Sample size 50
Data analysis 51
Data collection, management and quality assurance 53
Timeline 54
SAFETY EVALUATION 55
Standard care 55
Documenting adverse events 55
Assessment of adverse event severity 56
Documenting serious adverse events 56
Intussusception risks, assessment and management 57
Assessment of causality 58
Dose modification 59
Data and Safety Monitoring Board 59
MONITORING AND AUDITING 61
ETHICAL CONSIDERATIONS 63
Summary of known and potential risks 63
Risk minimization and benefits 63
Informed consent 64
Confidentiality 65
Reimbursement 66
Storage of specimens 66
Institutional Review Board approval 66
Declaration of conflict of interests 66
STUDY ADMINISTRATION 67
Protocol amendments 67

Protocol deviations and violations 67
Ancillary care and insurance 67
Data storage and archival 68
Dissemination and authorship policy 68
Data sharing policy 68
STUDY MANAGEMENT 69
Study sponsors 69
Serum Institute of India, Limited 69
Scientific Committee 69
Data and Safety Monitoring Board 69
Human resources 70
Training 72
REFERENCES 74

APPENDICES

Appendix A. Informed consent documents

Appendix B. Standard operating procedures for the collection, analysis and storage of biological specimens

Appendix C. Terms of Reference and Membership, Scientific Committee

Appendix D. Declaration of Helsinki

BACKGROUND AND RATIONALE

Epidemiology of childhood diarrhea

Acute diarrhea remains one of the major causes of morbidity and mortality among children, accounting for 11% of child deaths worldwide, more than malaria (7%) and far greater than HIV (2%) (1). Diarrhea-specific mortality has decreased in recent decades, but worldwide it is estimated to still account for 800,000 deaths in children < 5 years of age each year (2) and there has been little progress in reducing the incidence of diarrheal illness (3). There remains an estimated 2.5 billion episodes of childhood diarrhea per year (4). Children < 2 years of age are thought to experience an average of three to five episodes of diarrhea per year, with the highest rates (six to eight episodes per year) among infants 6-11 months old (5).

Acute diarrhea in children is often caused by a diverse group of infectious agents, including viruses, bacteria, and parasites. Transmission may occur through fecal-oral routes, respiratory secretions, or fomites (inanimate objects such as kitchen utensils). The recently completed Global Enteric Multicenter Study (GEMS) provides new evidence regarding the incidence, etiology and clinical outcome of moderate-to-severe diarrhea in seven sites throughout sub-Saharan Africa and south Asia (6). Using a comprehensive panel of microbiological assays to identify the etiology of moderate-to-severe diarrhea, a substantial proportion of diarrheal disease was attributed to 4 pathogens: rotavirus, *Cryptosporidium*, and heat stable toxin producing enterotoxigenic *E.coli*, and *Shigella*. In the first two years of life, the attributable incidence of moderate-to-severe diarrhea was dominated by rotavirus: incidence in infancy (7 episodes per 100 child-years) was more than double that of any other pathogen.

Current strategies for the management of childhood diarrhea

Acute diarrhea is rapidly dehydrating and can be life-threatening unless fluid therapy is initiated. In 1978, the World Health Organization (WHO) and United Nations Children's Fund (UNICEF) adopted oral rehydration salts (ORS) solution as the primary tool to fight dehydration. For more than 25 years, ORS was the only drug recommended by WHO and UNICEF for the prevention and treatment of dehydration. Rehydration therapy, however, does not decrease stool output, nor the duration or incidence of diarrhea, and ORS uptake is low: it is estimated that only one-third of children with diarrhea in developing countries currently receive ORS for treatment of their illness(4).

In 2004, WHO and UNICEF released revised recommendations for the management of diarrhea that also included therapeutic zinc supplementation for 10 to 14 days and incorporated zinc in the WHO Essentials Medicines List making it possible for zinc to be stocked in UNICEF warehouses (7). Despite the extensive body of scientific evidence supporting zinc for diarrhea management (8), many developing countries have been slow to adopt zinc as an explicit component of their diarrhea control programs, and zinc tablets appropriate for young children remain largely unavailable (4).

To accelerate progress towards reducing childhood diarrhea, calls for continued scaling up of proven interventions continue (9). In 2009, UNICEF and WHO issued the report, 'Diarrhea: why are children still dying and what can be done,' and called for the large-scale implementation of 7 interventions for comprehensive diarrhea control in developing countries. The plan includes a treatment package: fluid replacement (with low-osmolarity ORS and continued breastfeeding or feeding) and zinc treatment to decrease diarrhea severity and duration; and a prevention package: rotavirus and measles vaccination, promotion of early and exclusive breast-feeding and vitamin A supplementation, promotion of hand washing with soap, improved water supply and quality including household water treatment and safe storage of household water, and community wide sanitation (4).

Epidemiology of rotavirus and public health response

Rotavirus is the leading cause of severe gastroenteritis in children and is responsible for an estimated 450,000 deaths per year in children < 5 years of age, with most of the deaths occurring in developing countries (10). In sub-Saharan Africa, the proportion of hospitalizations for diarrhea associated with rotavirus ranges from 29% to 52% (11), with a global estimate of 34% in a meta-analysis of recent studies published between 2006 and 2008 (12).

Rotavirus genotypes are based on the two structural proteins of the virus outer capsid, VP7 (G glycoprotein) and VP4 (P protein). Four genotypes (G1P[8], G2P[4], G3P[8], G4P[8]) were historically recognized to be the most frequent, representing 88% of all strains worldwide in a meta-analysis from 2005, with the single G1P[8] genotype responsible for over 70% of infection in North America, Europe and Australia (13). More recently, G9P[8] has emerged worldwide and other rare genotypes, such as G12 and G6, are also emerging in Asia and Africa (14-16). Overall, recent studies have shown a wider variety of rotavirus genotypes circulating in developing countries compared to industrialized countries (11, 17).

Two rotavirus vaccines are currently available and prequalified by the WHO. Rotarix (GlaxoSmithKline) is a live, attenuated vaccine derived from the human 89-12 strain which belongs to G1P[8] type, while Rotateq (Merck) is a pentavalent human-bovine reassortant strain containing the G1, G2, G3, G4 and P[8] proteins. These vaccines have been shown to be safe and efficacious in high and middle-income countries (18, 19), where the impact of their introduction in routine vaccination programs on rotavirus-related hospital admissions and deaths has been demonstrated (20-24). Recent vaccine trials in Africa and Asia have shown substantially lower vaccine efficacy in low-income countries than in high or middle-income countries, with efficacies ranging from 50% to 64% (25-27). Despite these reduced efficacies, WHO has extended their recommendation for rotavirus vaccine introduction to all countries based on the higher predicted number of deaths averted in low-income countries (28).

Most of the difference in vaccine efficacy between high and low-income countries may be due to lower immunogenicity of oral live vaccines in developing countries (29, 30). The mechanisms underlying lower immunogenicity in developing countries remain poorly understood, but

enteropathy and malnutrition are thought to be major factors responsible for the reduced immune response to oral vaccines (31, 32).

Whether the greater variety of locally circulating genotypes and lower correlation with vaccine genotypes also contribute to the reduced efficacy remains a question for debate. Pooled data from randomized controlled trials show that the G1P[8]-containing Rotarix vaccine offers some protection against non-G1 and non-P[8] strain (33), but vaccine effectiveness studies in countries that have seen the emergence of the fully heterotypic G2P[4] genotype after introduction of Rotarix have shown contradictory results on heterotypic protection (34, 35).

Countries that have introduced rotavirus vaccines into their immunization programs have seen an improvement in child health. Recent studies show the swift and significant impact of rotavirus vaccines following introduction in national immunization programs. In Mexico, diarrheal deaths in children < 5 years of age decreased by 46% during 2007-2009 (36). In Australia, Belgium, El Salvador and the United States, hospitalizations and clinic visits for rotavirus-related diarrhea in children < 5 years of age declined by 60-94% between 2007 and 2010 (22). This reduction of severe diarrhea underscores the potential for rotavirus vaccines to save children's lives. Since 2011, Sudan, Ghana, Rwanda, Moldova, Yemen, Malawi, Armenia and Tanzania have introduced rotavirus vaccines into their national immunization programs. At present, 22 other countries in sub-Saharan Africa are expected to introduce rotavirus vaccines over the next several years (37).

Ongoing support from the Global Alliance for Vaccines and Immunization (GAVI) allows eligible countries to purchase vaccine at reduced cost, but the sustainability of time-limited vaccine subsidies remains a concern. Further, introducing current presentations of rotavirus vaccine into existing immunization programs may substantially disrupt the vaccine supply and cold chains. The added volume of new vaccines could displace other Expanded Programme on Immunization (EPI) vaccines from storage and transport space, overwhelm transport and storage at lower levels of the supply chain, and reduce the availability of all EPI vaccines at health centers where they are delivered. This scenario was born out during the 2006 to 2007 RotaTeq and Rotarix introductions in 7 Latin American countries. RotaTeq and Rotarix were too large for many of the existing supply chains, surpassing refrigerator capacities of many health centers and forcing health care workers to carry extra thermoses and cold boxes (38). Because no contingency plans were in place, these unexpected consequences resulted in the expiration of large stocks of vaccines. While this experience compelled manufacturers to re-design their vaccine packaging, it also underscores the possibility for new vaccines to not fit smoothly into supply chains, failing to reach their target populations and preventing other vaccines from reaching clinics.

Rotavirus in Niger

Since 2009, Epicentre has led a large-scale surveillance effort to gather data on the epidemiology of rotavirus in urban and rural Niger (39). From December 2009 to March 2012, 10,597 children aged 0-59 months presenting to health facilities in Niamey and Maradi with

watery diarrhea and signs of dehydration were enrolled. Stool specimens were systematically collected at presentation, with a rapid test performed on-site to determine the presence of rotavirus and genotyping performed on a subsample of rotavirus-positive specimens to determine genotype distribution and evolution during the study period. Surveillance identified 30.4% (95% CI: 29.6-31.3) of diarrhea to be rotavirus-positive, with 80% of all rotavirus cases found among children < 1 year of age and 96% of all cases found among children < 18 months. A higher proportion of rotavirus was found among cases in rural health centers than urban hospitals (32.4% versus 23.3%). Severe rotavirus diarrhea represented 1.2% of cases among children < 5 years of age and 3.0% among children < 1 year of age. Cases were seen year-round, with a consistent peak in the dry and cool season (October to December) and a natural switch from G2P[4] to G12P[8] genotype predominance observed during the study period. With 30% of children aged 0-59 months with diarrhea and dehydration positive for rotavirus, these results confirm the high burden of rotavirus in Niger, particularly in children < 18 months of age.

The Ministry of Health of Niger was formally approved for a GAVI-subsidized introduction of rotavirus vaccine in 2013, but delayed introduction until August 2014 due to concerns about vaccine presentation, storage and cold chain requirements among others.

Rotavirus Vaccine of Serum Institute of India, Limited

The currently in-development rotavirus vaccine (live attenuated bovine-human [UK] reassortant rotavirus vaccine) manufactured by the Serum Institute of India, Limited (SIIL) holds great promise for immunization programs in countries like Niger. The pentavalent vaccine (BRV-PV) contains rotavirus serotypes G1, G2, G3, G4 and G9 (>5.6 log10 FFU/serotype/dose) and is delivered in lyophilized form supplied with 2.5 ml of citrate bicarbonate buffer that is added for reconstitution just before oral administration. The proposed schedule includes a three-dose series of oral vaccine administered, within EPI. The initial dose is given at 6-8 weeks of age, with each subsequent dose given following a 4-week interval.
Compared to the two WHO prequalified vaccines, the SIIL formulation introduces important advantages for immunization programs (Table 1). First, BRV-PV is expected to be more affordable than available vaccines, with the potential for dramatic cost savings after 2015 when GAVI subsidies can expire. Second, BRV-PV offers the unique potential to be delivered out of cold chain. BRV-PV was tested and found to be stable at 37°C for 1 year and 40°C for 6 months; if delivered out of cold chain, this formulation could introduce significant logistical advantages for national programs in sub-Saharan Africa where cold chain capacity is limited.

Clinical evaluation of BRV-PV vaccine

Because of the promising safety and immunogenicity profile observed in a phase I clinical trial, SIIL conducted a two-center, double-blind, randomized, placebo-controlled phase II clinical trial with three doses of the pentavalent rotavirus vaccine in Pune, India. Sixty healthy infants received three administrations containing Log10 5.6-5.8 FFU/dose or placebo at 8-10 weeks, 12-14 weeks & 16-18 weeks of age, with at least 4 weeks interval between each dose. Safety

parameters included recording of solicited symptoms in the 14-day follow-up period after each dose, monitoring and recording of adverse events (AEs) and serious adverse events (SAEs) in the post-dose 28 day follow-up period, and changes in laboratory parameters including hematology, biochemistry, changes noted during physical examination and vital signs assessment. In total, 132 AEs were reported during the study, and 26 (86.7%) participants in the vaccine arm reported at least one AE during the study compared to 21 (67.7%) participants in placebo arm. Most reported adverse events were mild in intensity (97.4% in vaccine group and 94.6% in placebo group), and all events recovered without any sequelae before the study completion. No SAEs were reported during the entire period of the study. Overall, as the type, frequency, and severity of adverse events observed in vaccine-treated participants appeared similar to those of placebo-treated participants, the SIIL rotavirus vaccine at 3 doses (Log10 5.6-5.8 FFU/dose) was found to be safe and tolerable in infants.

Table 1. Summary of rotavirus vaccines

	Rotarix (GlaxoSmithKline)	Rotateq (Merck)	BRV-PV (SIIL)
Origin	Human monovalent	Bovine pentavalent	Bovine pentavalent
Genotype(s)	G1, P[8]	G1, G2, G3, G4, P[8], G6P[7]	G1, G2, G3, G4, G9
Vaccine course	2 doses – oral	3 doses – oral	3 doses – oral
Schedule	With DTP1 / 2	With DTP1 / 2 / 3	With DTP1 / 2 / 3
Age restrictions	First dose at 6-15 wk of age; Max age for last dose at 32 wk	First dose at 6-15 wk of age; Max age for last dose at 32 wk	First dose at 6-8 wk of age; Max age for last dose at 24 wk
Intussusception risk	None observed	None observed	None observed
Presentation	Lyophilized and reconstituted; or liquid	Liquid, single dose pouch	Lyophilized and reconstituted
Volume per dose	259.8 cm^3/1 dose box	798 cm^3/10 dose box	202.5 cm^3/1 dose box
Storage	2-8^oC; diluent at room temp	2-8^oC	Controlled temperature chain (2-25^oC)
Price (USD)	$2.5 / dose (GAVI)	$5 / dose (GAVI)	< $ 2 / dose (expected)
WHO pre-qualification	2007	2008	Not applicable

Sero-conversion rates at 28 days post-Dose 3 in the phase II trial were 60.0% in the vaccine arm and 7.7% in the placebo arm, indicating that the vaccine is highly immunogenic as compared to placebo. The net sero-conversion rate of 52.3% [60.0% (BRV vaccine) – 7.7% (placebo)] observed in this study is similar to that observed with Rotarix and Rotateq vaccines in India and other developing countries (40-49). At baseline, 23.3% of participants in the vaccine arm and 38.5% of participants in the placebo arm had an IgA concentration of ≥20.00 units/ml, indicating an early exposure to natural rotavirus infection. These results were not unexpected, as similarly high initial sero-conversion rates (in the absence of prior vaccination) have been observed in South Asia in past surveillance and previous rotavirus vaccine studies (40, 41, 50). Nevertheless, the good immunogenic response seen in initially sero-positive infants (57.1%) implies that the vaccine can successfully colonize the infant gut, induce a robust immune

response and significantly increase initial antibody levels, even in the presence of pre-existing IgA antibodies.

In summary, preliminary study has demonstrated that the SIIL rotavirus vaccine has a similar reactogenicity profile compared to placebo and is immunogenic in an environment where a substantial proportion of infants are initially sero-positive. These encouraging results justify conducting a phase III clinical trial to evaluate the protective efficacy of the SIIL rotavirus vaccine with dose ≥Log_{10} 5.6 FFU/serotype/dose.

Permission for a phase III clinical trial was granted by the Drug Controller General of India in May 2013. This will be a double-blind, placebo-controlled study to assess efficacy of the SIIL pentavalent vaccine in prevention of severe rotavirus gastroenteritis. The multi-centric study will be conducted in 6 sites across India in collaboration with the Program for Appropriate Technology in Health (PATH) and has been approved by the Western Institutional Review Board, USA and the institutional ethics committee at each study site. The study was initiated in May 2014 and will enroll 7500 children.

Study rationale

Sub-Saharan Africa carries the largest burden of rotavirus-related mortality, but immunization against rotavirus presents unique challenges. Current supply of the 2 WHO prequalified vaccines is constrained (51), and in many African settings, national immunization programs are challenged by supply shortages and a lack of trained health workers. Unreliable transportation systems and storage facilities also make it difficult to preserve vaccines that require refrigeration. If rotavirus vaccine is to be brought to the infants that need it most through national immunization programs in the region, new vaccines that address these challenges are urgently needed. The BRV-PV vaccine is a relatively low-cost and heat-stable formulation whose introduction into national immunization programs may help minimize the burden on already-strained national programs throughout sub-Saharan Africa.

The WHO Expert Committee on Biological Standardization has recommended that the efficacy of new rotavirus vaccines be demonstrated in diverse geographical regions including developing countries before widespread implementation (52). The Ministry of Health of Niger, Médecins Sans Frontières (MSF) – Operational Center Geneva and Epicentre along with other partners have formed a research consortium to bring additional evidence to inform public health decision making on the potential value of the BRV-PV vaccine in an African setting. The goal of the present study is to collect additional data on the efficacy profile of BRV-PV vaccine in a randomized controlled setting, while gaining further experience with vaccine-related adverse events, and immunogenicity. In recognition of the lower efficacy of oral vaccines observed in developing countries and need to identify potential boosters of immunogenicity in these settings, additional data will be collected with the aim of identifying complementary interventions to increase vaccine efficacy in developing countries. This will be conducted through the performance of a phase III trial in Niger conducted in compliance with the version

of the protocol agreed to by the applicable regulatory authorities and Good Clinical Practice (GCP).

Evidence supporting the efficacy, safety and immunogenicity of the BRV-PV vaccine in an African setting would support the pre-qualification of and increased global access to this formulation. If shown to be efficacious and pre-qualified, the government of Niger would benefit from a low cost vaccine adapted to the logistical and supply demands of the national immunization program.

STUDY OBJECTIVES AND ENDPOINTS

Primary objective:

To estimate the efficacy of three doses of SIIL BRV-PV vaccine vs. placebo against severe rotavirus gastroenteritis in healthy infants in Niger.

Secondary objectives:

- To estimate the efficacy of the SIIL BRV-PV vaccine vs. placebo against severe rotavirus gastroenteritis from 28 days post-Dose 3 to 1 year of age and from 1 to 2 years of age, from 28 days post-Dose 3 to 2 years of age.
- To evaluate vaccine efficacy against rotavirus gastroenteritis of any severity.
- To estimate vaccine efficacy against rotavirus gastroenteritis with a Vesikari score ≥ 17.
- To estimate vaccine efficacy against gastroenteritis of any cause.
- To estimate vaccine efficacy against severe rotavirus gastroenteritis caused by G serotypes included in the vaccine (G1, G2, G3, G4 and G9).
- To estimate longitudinal prevalence of rotavirus gastroenteritis.
- To estimate vaccine efficacy to reduce hospitalizations due to rotavirus gastroenteritis.
- To estimate vaccine efficacy to reduce hospitalization for any reason.
- To estimate safety of the SIIL BRV-PV vaccine vs. placebo (adverse events [AEs] and serious adverse events [SAEs]).
- To estimate the immunogenicity of BRV PV in a sub-sample of participants.
- To estimate effect of prenatal nutritional supplementation on infant immune response to the vaccine in a sub-sample of participants.
- To demonstrate the immunological non-inferiority of EPI vaccines when co-administered with the BRV-PV as compared to their co-administration with placebo

Primary endpoint:

The primary endpoint is vaccine efficacy of three doses of the SIIL BRV-PV vaccine vs. placebo against a first episode of laboratory confirmed severe rotavirus gastroenteritis from 28 days post-Dose 3 until 117 cases are accrued or when all participating infants reach 2 years of age if 117 cases are not attained.

Secondary endpoints:

Efficacy:

- Laboratory-confirmed cases of severe rotavirus gastroenteritis from 28 days post-Dose 3 to 1 year of age and from 1 to 2 years of age, from 28 days post-Dose 3 to 2 years of age
- Laboratory-confirmed episodes of rotavirus gastroenteritis of any severity
- Laboratory-confirmed episodes of rotavirus gastroenteritis with a Vesikari score of ≥ 17

- Laboratory-confirmed cases of severe rotavirus gastroenteritis due to rotavirus serotypes G1, G2, G3, G4 and G9, i.e., the serotypes included in the vaccine
- Episodes of gastroenteritis of any cause
- Longitudinal prevalence of laboratory-confirmed rotavirus gastroenteritis
- Hospitalization due to laboratory-confirmed cases of rotavirus gastroenteritis of any cause
- Hospitalization of any cause

Safety:

- Adverse events from the time of Dose 1 to 28 days post-Dose 3
- SAEs in all participants through two years of age

Immunogenicity

- Anti-rotavirus IgA seroresponse rates and geometric mean titres in a subset of 1320 infants

Immune interference with EPI vaccines:

- Neutralizing antibody titers to polio virus serotypes 1, 2 and 3 ≥ 1:8 (seroprotection)
- Antibody concentrations to Diphtheria toxoid ≥ 0.1 IU/mL (seroprotection)
- Antibody concentrations to Tetanus toxoid ≥ 0.1 IU/mL (seroprotection)
- Antibody concentrations to Pertussis expressed as GMCs
- Antibody concentrations to Hepatitis B (anti-HBs) ≥ 10 mIU/mL (seroprotection)
- Antibody concentrations to Hib (anti-PRP antibodies) ≥ 1 mcg/mL (long term seroprotection)

METHODOLOGY

Study design

The study is designed as a double-blind, placebo-controlled randomized phase III event-driven trial with two parallel groups of infants in Madarounfa, Niger to assess the efficacy, safety and immunogenicity of BRV-PV, a pentavalent rotavirus vaccine manufactured by SIIL. The primary aim of the study is to assess the efficacy of three doses of pentavalent rotavirus vaccine vs. placebo against severe rotavirus gastroenteritis from 28 days post-Dose 3 until 117 cases are accrued, or until all participating infants reach 2 years of age if 117 cases are not attained when administered within EPI. A total of 7,770 infants aged 6-8 weeks (42-56 days) will be individually randomized in a 1:1 ratio using permuted blocks of random sizes to receive three doses BRV-PV or placebo. The first dose will be administered at 6-8 weeks (42-56 days) of age followed by two more doses at 4 week (–1 to +4 weeks) intervals. The primary endpoint is vaccine efficacy of three doses of the SIIL BRV-PV vaccine vs. placebo against a first episode of laboratory confirmed severe rotavirus gastroenteritis from 28 days post-Dose 3 until 117 cases are accrued or when all participating infants reach 2 years of age if 117 cases are not attained. Gastroenteritis is defined as three or more looser-than-normal stools in a 24-hour period with or without vomiting. Episodes of gastroenteritis will be identified through facility- and home-based surveillance until 2 years of age (see Table 2).

As an event-driven trial, the primary efficacy analysis of the study will be conducted when 117 cases of severe rotavirus gastroenteritis are identified from 28 days post-Dose 3, or when all study participants reach 2 years of age if 117 cases are not attained. In the event that 117 cases are accrued before completion of recruitment, recruitment will not be interrupted and follow up will continue until all participating infants reach 2 years of age. All secondary efficacy analyses will be conducted on cases accrued up until the time when the primary objective is fulfilled (i.e., 117 SRVGE cases accrued), as well as at the end of the study. Secondary efficacy analysis will be conducted in the per protocol cohort (children receiving all three scheduled rotavirus vaccine/placebo and without any major protocol deviation), as well as the intention to treat cohort (all randomized children).

Secondary aims of the study include the assessment of safety and immunogenicity of three doses of BRV-PV. All infants will be followed for the incidence of adverse events (to 28 days post-Dose 3) and serious adverse events (to 2 years of age). Immunogenicity of the BRV-PV vaccine, as well as the effect of prenatal nutritional supplementation on immunogenicity of the BRV-PV vaccine and non-interference with concomitant EPI immune response, will be assessed in a nested sub-study among the sub-sample of participants (“immunogenicity sub-cohort”) 28 days post-Dose 3.

Placebo, instead of an active comparator, will be used in this trial Placebo-controlled data from an African setting are needed to support eventual WHO prequalification of BRV-PV (see Choice of Placebo Comparator below).

An independent DSMB will be established to monitor the conduct of the trial. The DSMB will periodically examine vaccine safety and provide recommendations to the Sponsor regarding continuation of the study.

Table 2. Schema of overall study design

WEEK OF AGE		W0	W6	W10	W14	W18	W24	W36	W52	W64	W76	W88	W104
**Study Visit**	Pregnancy	Pre - random-ization	Random-ization and Dose 1	Dose 2	Dose 3	Dose 3 + 28 days	6 months	9 months	12 months	15 months	18 months	21 months	24 months
**ALL PARTICIPANTS**													
Home Visit*			⟶	⟶	⟶	⟶	⟶	⟶	⟶	⟶	⟶	⟶	⟶
Scheduled Facility Visit			X	X	X	X	X	X	X		X		X
Surveillance													
Gastroenteritis and SAE			X	X	X	X	X	X	X	X	X	X	X
AE**			X	X	X	X							
Laboratory Assessment													
Child stool***			X	X	X	X	X	X	X	X	X	X	X
**IMMUNOGENCITY SUB-COHORT**													
Home Visit*	⟶	⟶	⟶	⟶	⟶	⟶	⟶	⟶	⟶	⟶	⟶	⟶	⟶
Laboratory Assessment													
Child stool***			X	X	X	X	X	X	X	X	X	X	X
Child blood			X			X			X				X
Child urine			X	X	X	X	X	X	X	X	X	X	X
Maternal blood	X	X	X				X						
Maternal stool	X	X	X				X						
Maternal urine	X	X											
Breast milk		X	X				X						

* Home Visits scheduled for Pre-randomization and on a weekly basis until 2 years of age. In the immunogenicity sub-cohort, home visits will be conducted among all consenting women of reproductive age and continue until the child is 2 years of age.

** Surveillance for adverse events from the time of Dose 1 until 28 days post-Dose 3.

*** Stool collected from all participants for any case of gastroenteritis identified at facility or home within a recall period of 7 days until 2 years of age. In the immunogenicity sub-cohort, stool samples will be collected independent of gastroenteritis status at the time of each Dose, 28 days post-Dose 3, 6 months of age and every 3 months thereafter until 2 years of age.

Study setting

Niger is one of the poorest countries in the world, ranking 186 of 187 in 2011 on the Human Development Index (53). Fertility is high, estimated by the 2006 Demographic and Health Survey (DHS) at 7·1 children per woman (54). While rates of child and neonatal mortality in Niger have been among the highest in the world, significant progress has been made with investments in maternal, child and newborn program and policy. Recent data suggests child mortality in Niger declined 43% between 1998 and 2009, from 226 to 128 deaths per 1000 live births (55). Progress in reducing neonatal mortality however has been slower, with the same data showing high levels of neonatal mortality and no significant reduction in neonatal mortality between 1998 and 2009 (39 vs. 33 neonatal deaths per 1000 live births).

The health system in Niger is a pyramidal system in line with the 1985 Lusaka agreements, based on health structures with increasing levels of service capacity: health posts (cases de santé) provide basic care and preventive services and are most often staffed by community health workers who are helped by community representatives. Health centers (centres de santé intégrés) are staffed by nurses and ensure the provision of all services not requiring hospitalization. Complications are referred to the district hospital and to the regional hospital from the district hospital. Integrated Management of Childhood Illness (IMCI) algorithms provide the basis for the organization of care and referral system for children. In this organization, severity signs are assessed at the level of health centers and only severe cases are referred to the district hospital, with the exception of severe dehydration, which should be treated immediately at the health center.

The current organization of the health system is based on several international initiatives launched in 1995-1996. In Niger, this period was marked by the creation of Health Districts for decentralization of care, the gradual introduction of IMCI in all districts, and the implementation of cost recovery systems following the Bamako Initiative. Despite these efforts, the most recent final report from the national DHS in Niger in 2006 showed that only 17% of caretakers of children < 5 years of age sought advice or treatment in case of diarrhea in their child (54). With the aim of reducing child mortality in line with the UN Millennium Development Goals, free care for children under 5 years of age was introduced in April 2007.

The study will take place in the region of Maradi, in south-central Niger along the Nigerian border. The region of Maradi is comprised of seven Health Districts with a total estimated population in 2009 of about 3 million inhabitants. The proposed site for this study is the Madarounfa Health District, a rural area of 4700 km^2 largely representative of the Sahel region of Niger and sub-Saharan Africa (56). In 2009, the average number of public health structures per 100 km^2 was 1.5 and the public health system coverage, defined as the proportion of the population with access to any health structure within a distance of less than 5 km, was 83% (57).

In collaboration with the Ministry of Health, MSF has been supporting pediatric care in the Madarounfa Health District of Maradi since 2001. Since 2009, project activities have been

transferred to local control and implemented through a Nigerien non-governmental organization, Forum Santé Niger (FORSANI) in collaboration with the Ministry of Health. FORSANI provides care and treatment to over 30,000 children in the Madarounfa Health District each year. Epicentre, the epidemiologic and research organization affiliated with MSF has been present in Niger since 2009. In close partnership with the Ministry of Health, MSF, and other partners working in the area, Epicentre develops and conducts research aimed at responding to the medical and operational objectives of local and regional public health actors. A team of medical professionals, epidemiologists, biologists and data management specialists work in Maradi and Niamey.

Investigational product (vaccine and placebo)

The study vaccine, BRV-PV, is a pentavalent rotavirus vaccine containing rotavirus serotypes G1, G2, G3, G4, and G9 manufactured by SIIL. Each dose of vaccine contains an estimated potency of ≥ 5.6 $\log_{10}$ infectious units / serotype per dose; dose selection was based on demonstrated immunogenicity in phase II trials. Placebo, also manufactured by SIIL, will contain the same constituents as the active vaccine but without the viral antigens; both are lyophilized and will be reconstituted with 2.5 ml of liquid citrate bicarbonate buffer before administration (see Table 3). The initial dose of study vaccine or placebo will be administered orally at a health facility by a study physician at 6-8 weeks of age. The second and third doses of study vaccine or placebo will each be administered at a health facility following a 4 week internal (-1 to +4 weeks).

All children will be referred to the local health authority for administration of EPI vaccines according to the national immunization schedule. Administration of the study intervention will not be delayed if EPI vaccines are unavailable at the time of study dosing. Infants who have already received EPI vaccines at the time of study dosing can still receive the study intervention. However in the sub-study where non-interference with EPI vaccines will be evaluated, infants will be administered EPI vaccines by the study team concomitantly with the study vaccine or placebo.

Table 3. Characteristics of study interventions

Study Vaccine	Live attenuated Bovine-Human (UK) reassortant pentavalent rotavirus vaccine containing ≥ $\log_{10}$5.6 FFU/dose of each serotype G1, G2, G3, G4 and G9
Placebo	Placebo, consisting of the lyophilized Eagle's MEM culture medium (6.63 mg), glycine (50 mg) and sucrose (50 mg)
Diluent	Buffered Diluent (2.5 ml containing 64 mg of sodium bicarbonate and 24 mg citric acid)
Manufacturer	Serum Institute of India Limited, India

Preparation and administration. The process for preparation and administration of vaccine and placebo will be fully detailed in the study Standard Operating Procedures (SOP). In brief, the

vaccine and placebo are dispensed as single dose and are for one time use only. The reconstituted vaccine and placebo, as well as the vial containing buffered diluent, should be inspected visually for any foreign particulate matter and/or abnormal physical appearance prior to administration. In the event either is observed, the vaccine or placebo will be discarded and a replacement kit will be used for vaccination.

BRV-PV and placebo are for oral use only and should under no circumstances be injected. Neither vaccine nor placebo should be mixed with other medicinal products. Procedures for reconstitution are as follows:

- Remove plastic caps from the vials containing buffered diluent and lyophilized powder.
- Fix transfer adapter to 5 ml disposable syringe (without needle).
- Connect syringe/adapter to the vial containing buffered diluent.
- Pull the plunger back and aspirate 2.5 ml of buffered diluent from vial into the syringe.
- Remove the assembly from buffer diluent vial and connect it with the vaccine or placebo vial containing lyophilized powder.
- Inject the entire contents of the syringe into the vial.
- With syringe still attached shake the vial and examine for complete suspension of the powder. The reconstituted vaccine or placebo will appear as a clear, pinkish solution.
- Pull the plunger back and aspirate reconstituted solution from vial into the syringe.
- Remove the syringe from the transfer adapter.

Study vaccine and placebo must be administered immediately after reconstitution as far as possible but not beyond one hour after reconstitution.

Packaging and labeling. Each single oral dose of study vaccine and placebo will be approximately 2.5 ml in volume. Packaging for both study vaccine and placebo will contain 1 vial of lyophilized vaccine/placebo, 1 vial of citrate bicarbonate buffer, 1 adapter and 1 syringe (without needle) of 5ml capacity for vaccine reconstitution. Only the specific buffer diluent provided must be used for reconstitution.

The vials used for administration of the study products will be labeled according to the local regulations and requirements of the study protocol. Label text will be approved by the sponsor prior to label printing. All labels will contain the following minimum information**:**

- Name of Sponsor and Manufacturer
- Imprint “For Clinical Trial Use Only”
- Imprint “For Oral use only”
- Blind code to identify content

Storage. The study vaccine and placebo will be stored between 2-8°C up from the time of supply by SIIL to arrival at the central facility in Maradi. Changes in temperature outside the allowed range before arrival at the central facility-level will be immediately reported and any

study product experiencing such out of range changes will be brought to the attention of the sponsor and manufacturer (by the sponsor) for determination of appropriate action.

After dispatch from the central facility-level in Maradi, study vaccine and placebo will be stored out of cold chain until administration. Ambient temperatures will be recorded on a daily basis at the Epicentre Maradi Weather Station to describe the conditions observed out of cold chain during the study period. Procedures for proper storage from the manufacturer (SIIL) to Niamey, the central facility level (Maradi) and to administration at the rural health facilities (Madarounfa) will be fully detailed in the study SOPs.

Study supply and accountability. All study vaccine and placebo will be provided by SIIL. The sponsor will acknowledge receipt of the vaccine and placebo indicating shipment content and condition. The sponsor will maintain an inventory record of study product received and account for all study products used in the study using appropriate accountability records.

Temperature will be monitored during shipment, and the sponsor will check and maintain temperature records on file. The sponsor will inform SIIL immediately of any shipment that is out of range. The randomization code of used study products will be recorded at the time of administration for accountability and used vials destroyed on site after verification by the Medical Monitor as per study SOPs.

The site will receive instruction from SIIL regarding the final disposition of any remaining study products. At the completion of the study (last subject last visit), there will be a final reconciliation of study products shipped, study products consumed, and study products remaining. Any discrepancies noted will be investigated, resolved, and documented prior to return or destruction of unused study products. All unused or partially used study products and packaging will be destroyed on site after verification and accountability by the Site Principal Investigator/Medical Monitor as per local regulations. A certificate of destruction will be generated and kept on file. The process for disposing of unused or partially used materials will be fully detailed in the study SOPs.

Choice of placebo comparator

Two vaccines are currently WHO prequalified, however, several issues arise with respect to their use in the evaluation of BRV-PV. A placebo-control, instead of an active comparator, has been selected for this trial given the following issues and ethical considerations (58, 59):

- It is known that the current oral vaccines have lower efficacy in low-resource settings than in high-resource settings. To inform eventual WHO pre-qualification and increased access to rotavirus vaccine, it will be important to demonstrate the absolute efficacy of new formulations in low-resource settings. A placebo-controlled design is needed to directly demonstrate absolute efficacy; an active-comparator design would determine relative efficacy and be less informative in the process towards WHO pre-qualification.

- There is no efficacy data on any licensed rotavirus vaccine in Niger, and a placebo-controlled trial remains defensible and appropriate where there is no “proven” intervention, as per the Declaration of Helsinki (Appendix D).

- Although children in the placebo group will not potentially benefit from vaccination during the study period, all children will benefit from prompt, free and appropriate primary care, minimizing the risks and enhancing benefits to participants. If shown to be safe and efficacious and pre-qualified, the vaccine should be made available to all children in Niger through the national immunization program.

- A non-inferiority trial using an active comparator would require a prohibitively large sample size and involve a disproportionate use of time and resources. It would be practically infeasible to conduct such a trial, and the resulting impact of such an investment has been subject to debate and criticism due to the potential delay in bringing rotavirus vaccines to children who stand to benefit most.

- Although rotavirus vaccine was introduced in Niger in August 2014 by the national regulatory authority (la Direction de la Pharmacie, des Laboratoires et de la Médecine Traditionnelle). Current global supply of the 2 WHO prequalified vaccines is constrained (51).

Prenatal nutrition intervention. Three prenatal nutrition supplements will be evaluated in the immunogenicity sub-cohort: 1) iron-folate (60 mg iron + 400 µg folic acid as standard of care during pregnancy); 2) multiple micronutrients with approximately two times the Recommended Dietary Allowance of micronutrients, and 3) 40 g lipid-based nutrient supplement specifically designed for pregnant and lactating women with energy, protein, fats and multiple micronutrients. Daily supplements will be provided on a weekly basis until pregnancy outcome. An acceptability test of the 40 g lipid-based nutrient supplement will be conducted prior to study initiation to ensure acceptability of the formulation among pregnant women in the study zone.

Randomization procedures

Infants will be individually randomized in a 1:1 ratio to receive three doses of the vaccine or placebo. The initial dose will be orally administered at 6-8 weeks of age. The second and third doses of study vaccine or placebo will each be administered following a 4 week interval (-1 to + 4 weeks). To assess the effect of prenatal nutrition supplementation on infant immune response to the BRV-PV vaccine, study villages in the immunogenicity sub-cohort will be randomized in a 1:1:1 ratio to provide women with daily iron-folate, multiple micronutrients or a lipid-based nutrition supplement in pregnancy. Infants of participating women, if eligible at 6-8 weeks of age, will be randomized in a 1:1 ratio to receive three doses of vaccine or placebo and enter the main trial as part of the immunogenicity sub-cohort.

The Contract Research Organization (CRO) will prepare a randomization list using a computer-generated random number list with permuted blocks of random sizes. The randomization list will contain a unique 4-digit code for each participant that contains no identifiable information. Block sizes will not be disclosed to reduce predictability of the random sequence and ensure allocation concealment. Study physicians who will oversee randomization will be given a subset of sequentially numbered silver coated booklets that will be prepared by the CRO and contain a printed randomization code. The study physician will be instructed to assign the next sequential randomization code noted in the booklet to each eligible infant as (s)he is enrolled. Adherence to the randomization list will be periodically verified by the Site Principal Investigator/Medical Monitor. A copy of the randomization list will remain with the CRO for the duration of the study; randomization will therefore be conducted without any influence of sponsors or field personnel. A copy of the randomization list as silver coated booklets will remain with a representative of the Sponsor not involved in study activities for the duration of the study.

Blinding procedures

Vaccine and placebo packages will be labeled with a randomization code and delivered to the study site in otherwise identical presentations. Vaccine and placebo presentation and packaging will be indistinguishable to ensure that caregivers and investigators cannot identify to which group the infant has been assigned. Group assignment will remain concealed from study team, investigators and parents /guardians of participating infants for the whole study period. The Data and Safety Monitoring Board (DSMB) and sponsor statistician will also be masked to the group assignment. The DSMB will remain masked unless otherwise deemed necessary by the DSMB members for any safety related issues. Investigators conducting the final analysis will remain masked to the group assignment until the time of final analysis.

The study code will be broken only in case of a medical event in which the Site Principal Investigator/Medical Monitor deems the participant cannot be appropriately treated without knowing his/her group assignment. A set of sealed envelopes with group assignment will be held at the field site with the Site Principal Investigator. All code breaks will be fully documented and reported to the sponsor within 48 hours, and the date, time and reason for unblinding will be noted. Codes will not be freely available to the sponsors or other study personnel until after the completion of the trial and final data review. Unblinding will not be sufficient reason for individual discontinuation from the study.

The prenatal nutrition intervention implemented in the immunogenicity sub-cohort will be open, as it will not be possible to blind participants or study staff to type of supplement received.

Target population

The study will be performed in infants in Madarounfa, Niger. Healthy male and female infants meeting the following inclusion criteria are eligible for enrollment:

(1) aged 6-8 weeks at the time of inclusion
(2) able to swallow and no history of vomiting within 24 hours
(3) resident in Madarounfa Health District and within the catchment area of the health facilities
(4) intending to remain in the study area for 2 years
(5) parent/guardian providing written informed consent

There will be no restriction based on breast feeding around the time of vaccination, receipt of routine pediatric vaccinations, prematurity, low birth weight or known HIV status (60). Need for immediate hospitalization, inability to swallow and history of vomiting within the last 24 hours are the only conditions based on the infant's immediate clinical status to delay oral administration of the study vaccine or placebo.

Exclusion criteria will include the following:

(1) known history of congenital abdominal disorders, intussusception, or abdominal surgery
(2) receipt of intramuscular, oral, or intravenous corticosteroid treatment within 2 weeks
(3) receipt or planned administration of a blood transfusion or blood products, including immunoglobulins
(4) any known immunodeficiency condition
(5) any serious medical condition
(6) any other condition in which, in the judgment of the Site Principal Investigator, would interfere with or serves as a contraindication to protocol adherence or the parent/guardian's ability to give informed consent*.

*While determining eligibility, study physicians will be asked to use good clinical judgment in considering a participant's overall fitness for inclusion. Some participants may not be appropriate for the study even if they meet all inclusion criteria. For instance, medical, occupational or other conditions of the caregiver may make routine home visits and evaluation difficult or make the child a poor candidate for retention. Any such potential exclusion must be validated by the Site Principal Investigator.

In the immunogenicity sub-cohort, all women of reproductive age (18 to 45 years) within the sub-study catchment area will be invited to participate in regular pregnancy surveillance. Women severely anemic (hemoglobin < 7 g/ dL) when pregnancy is confirmed will be excluded from participation in the prenatal nutritional intervention. All children in the sub-study catchment will be evaluated for eligibility for the immunogenicity sub-cohort at 6-8 weeks of age regardless of participation in the prenatal nutritional intervention.

Study sites

Study activities will take place in the Madarounfa Health District, Maradi, Niger. Health facilities in the Madarounfa Health District, including 1 hospital, 5 central health centers and 12 health posts in the 5 rural health zones of Gabi, Safo, Madarounfa, Dan Issa, and Tofa will be included as Surveillance Sites, where a study physician or nurse will be present to assess symptoms of gastroenteritis and adverse events. All 5 central health centers will be designated as Enrolment and Dosing Sites. Enrolment and Dosing Sites will be staffed by study physicians / designees trained to seek written informed consent and equipped with appropriate emergency medical treatment in the event of any adverse event or serious adverse event 30 minutes post-immunization. The number and location of Enrolment and Dosing Sites has been made to ensure reasonable access by the study population and in consideration of available human resources.

Additional study activities will take place at the participants' home (see Recruitment and Follow up for further detail).

Study procedures

Recruitment and screening

Rotavirus in Niger is known to circulate year-round in the Maradi region (with a consistent peak in October to December), therefore enrolment will occur year-round. In the event that 117 cases are accrued before recruitment of 7770 infants, an intermediate analysis can be triggered but recruitment and follow up of all 7770 children to 2 years of age will not be interrupted.

Eligible children will be continuously identified during an approximate 18-month enrollment period using community health agents selected from each village. These individuals are intended to be well regarded and trusted members of the community with broad knowledge of community events, such as births, deaths and migration. Community health agents will record all pregnancies , births and infant in-migrations in his/her village. The registry will be routinely updated and transferred to study physicians and nurses throughout the enrollment period.

Within about 48 hours of a live birth, a study nurse will visit mothers at home for a **Pre-randomization Visit**, at which time the nurse will provide the household with information about the study. The Pre-randomization Visit will allow caregivers adequate time to consider their participation in the trial and prepare any questions they may have, thereby reducing the number of defaulters once randomization has occurred. At this time, oral informed consent will be sought specifically to allow recording of maternal mid-upper arm circumference (MUAC), infant birth weight using standard field procedures, and gestational age at birth using the New Ballard score (61). Written informed consent to participate in the full trial will be sought at the time of randomization between 6-8 weeks of age. Infants < 8 weeks of age who migrate into the study area will be identified by community health agents and similarly visited at home by a study nurse for a Pre-randomization Visit. At all Pre-randomization Visits, the study nurse will confirm receipt of routine vaccinations scheduled for administration at birth [e.g. Bacillus Calmette-Guérin and oral poliovirus vaccines] through review of the infant's vaccination card, and as needed, invite the caregiver to have the child vaccinated at the nearest health facility.

At 6-8 weeks of age, the community health agent will ask caregivers to present with their child to the nearest Enrollment and Dosing Site for the **Randomization Visit**. Final eligibility determination will depend on the results of the medical history, clinical examination, fulfillment of the inclusion and absence of any of the exclusion criteria, appropriate understanding of the study and completion of the written informed consent process.

Investigators should always use good clinical judgment in considering a participant's overall fitness for inclusion in the trial. Some participants may not be appropriate for the study even if they meet all inclusion/exclusion criteria. For instance, medical, occupational or other conditions present in the parents/guardians may make safety evaluations difficult or make the infants poor candidates for retention. All infants targeted for enrollment will need to have parents/guardians who can comprehend the purpose of the study and provide written informed consent. Sufficient number of healthy infants will be screened to enroll 7770 participants with written parental consent in the study.

Issues of confidentiality will be underscored. No financial or non-financial incentives will be provided to study staff for enrolment.

If during the eligibility evaluation a child is found to require immediate hospitalization, be unable to swallow or to have history of vomiting within 24 hours but is otherwise eligible for study inclusion, the study physician can delay study procedures. The caregiver and infant will be asked to return to the Enrollment and Dosing Site 24 hours after symptoms resolve but before 8 weeks of age to continue study procedures. If a child is found to be entirely ineligible for inclusion, the child will be recorded as not included on an Eligibility Evaluation Case Report Form (CRF), with the reason for non-inclusion noted.

In the immunogenicity sub-cohort, all women of reproductive age (18-45 years) resident in villages within the sub-cohort catchment will be invited to participate and provide written informed consent. Every two weeks, the community health agent will visit participating women and administer a short questionnaire to determine the date of last menstrual period. When a menses has been missed, a urine pregnancy test will be conducted at home and women with a positive test will begin to receive the assigned nutritional supplementation until pregnancy outcome. All infants identified in the catchment will receive a standard Pre-randomization Visit and be evaluated for eligibility for entry into the main trial as part of the immunogenicity sub-cohort at 6-8 weeks of age regardless of participation in the prenatal nutrition intervention.

The recruitment period will extend approximately over 18 months. We anticipate recruiting 108 infants per week in 5 rural zones of the Madarounfa Health District [e.g. Gabi, Safo, Madarounfa, Dan Issa, and Tofa], where there were 9597live births in 2013 (62).

Randomization and inclusion

Once written informed consent for participation in the full trial is provided by the parent/guardian, the child will be enrolled in the study and a blinded randomization code

assigned for administration of vaccine or placebo. The unique randomization code will be allocated by CRO using computer-generated random number list using permuted blocks of random sizes.

A detailed background questionnaire will be administered to the caregiver, including information on maternal age, nutritional status (e.g. weight, height and MUAC) and reproductive history (e.g. parity, age of first delivery); household size and socio-demographic characteristics; infant's medical and breast feeding history; and infant anthropometry. The caregiver will receive a study identification card at this time, which should be shown at the time of presentation to any health facility.

Vaccination

A three-dose series of study vaccine or placebo will be orally administered to infants with the initial dose given at time of the Randomization Visit (6-8 weeks of age) and each subsequent administration given after a 4-week interval (-1 to +4 weeks). All doses of study vaccine or placebo will be administered at a designated Enrollment and Dosing Site by a trained study physician. Study vaccination may be delayed if child is found to require immediate hospitalization, be unable to swallow or have history of vomiting. The caregiver and infant will be asked to return to the Dosing Site 24 hours after symptoms resolve for the next administration of the study intervention. If a child is found to have gastroenteritis at any Dosing Visit, dosing will not be delayed but a stool sample will be collected for detection of rotavirus antigen.

A Dosing CRF will be completed to record information on the study product received, date and time. If a child vomits any of the study product immediately after administration, a new dose will be administered and the event will be recorded on the appropriate Dosing CRF. A repeat dose will not be administered if the child drools or spits up any of the study product. Study staff will remain with the infant for a minimum of 30 minutes to record any adverse event or serious adverse event post-immunization. Any reactions that occur during this time will be recorded on the standardized Dosing CRF, and appropriate emergency medical treatment will be readily available in case of any reaction following administration of a study product. Breastfeeding 30 minutes before or after administration of the study product will be documented on the Dosing CRF.

At each Dosing Visit, the study physician will verify previous receipt of any EPI vaccine through review of the infant's vaccination card, and as needed, refer the child to the local health authority for administration of any required routine EPI immunization (see Standard Care below for schedule of EPI vaccines to be provided). Administration of the study intervention will not be delayed if EPI vaccines are unavailable at the time of study dosing. Infants who have already received EPI vaccines at the time of study dosing (e.g. oral polio vaccine administration provided through routine mass vaccination campaigns) will still receive the study intervention as scheduled. Infants in the immunogenicity sub-study cohort, where non-interference with EPI

vaccines will be evaluated, will receive all EPI vaccines scheduled at 6, 10 and 14 weeks of age concomitantly with the study intervention.

If a child does not present to the health facility for the Dose 2 or Dose 3 Visit as scheduled, the study team will conduct a home visit to encourage the caregiver to present to the health facility as soon as possible. If the child cannot be seen within -1 to +4 weeks of the scheduled dosing visit, the child will remain eligible to receive the study dose at the time of next presentation to the health facility and will continue follow up without modification for consideration in the secondary intention to treat analyses. However, children in the immunogenicity sub-study cohort must follow the schedule and if they miss -1 to +4 weeks of the scheduled dosing visit, the child will not be included in immunogenicity analysis.

Assessment of compliance

Study vaccine or placebo will be orally administered by trained study physicians only to infants included in this study. The date, dosage, and time of the vaccinations will be recorded on a standardized Dosing CRF. The Site Principal Investigator/Medical Monitor will track vaccines received, used and wasted and will manage all unused or expired products.

In the immunogenicity sub-cohort, compliance with the prenatal nutrition supplement will be assessed through the weekly collection of unused supplements at home.

Immediate post-vaccination safety assessment

Study vaccine or placebo will be orally administered by trained study physicians. The date and time of the vaccinations will be recorded on a standardized Dosing CRF. Study staff will remain with the infant for a minimum of 30 minutes to record any adverse event or serious adverse event post-immunization. At 30 minutes post-vaccination, vital signs will be measured, and if indicated, a targeted physical examination will be performed. Any reactions that occur during this time will be recorded on the standardized Dosing CRF, and appropriate emergency medical treatment will be readily available in case of any reaction following administration of a study product. Breastfeeding 30 minutes before or after administration of the study product will be documented on the Dosing CRF.

Follow up

All children will be followed from the time of the first dose until 2 years of age (gastroenteritis and serious adverse events) and from the time of first dose to 28 days post-Dose 3 (adverse events) using both facility- and weekly home-based follow up.

Scheduled and interim facility visits: Caregivers will be informed about the signs and symptoms of gastroenteritis and all adverse events, and they will be asked to immediately seek care at a local facility if there is any condition that requires medical attention or is of concern. Upon presentation at the facility, study staff will conduct a history of symptoms and medical

interventions received for the current illness and perform a clinical examination to document physical signs and indicators of disease severity. Appropriate medical care will be provided in accordance with GCP. If an episode is ongoing at the time of discharge from the health facility (up to 28 days post-Dose 3 for adverse events), daily home-based follow up by a nurse assistant will be organized from the time of discharge until resolution of the episode. Facility-based follow up will also include regular facility visits with a physical examination by study staff at 6, 9, 12, 18 and 24 months of age.

Weekly home-based follow up: **Weekly Home Visits** will be performed by a community health agent. At each weekly visit, the community health agent will ask caregivers to recall all medical signs or symptoms that are a concern to them at that time and review the Weekly Diary Card completed by caregivers to confirm information on events since the last visit, including cases of gastroenteritis, adverse events, events to be reviewed as potential serious adverse events, and medical intervention (including treatments received, and admission to hospital or therapeutic nutritional programs after 6 months of age). At each weekly visit, the community health agent will remind caregivers to immediately contact the study team (the community health agent in the village, or the study physician or nurse at the health facilities) in the event of any episode of gastroenteritis,adverse event, or serious adverse event.

Children found to require medical intervention during the Weekly Home Visit will be referred to a study health facility for management free of charge by study staff and in accordance with GCP. Every 4 weeks, the child's anthropometric status (e.g. weight, height and MUAC), breastfeeding status (e.g. ever vs. never; currently exclusive vs. partial vs. no breastfeeding; frequency of feeds in last 24 hours) and dietary intake will be assessed at home using standard procedures with assistance from the caregiver. If a child is not present at the time of a Weekly Home Visit, the community health agent may contact neighbors or relatives in the area to ascertain the infant's vital and health status and expected time/day of return so that a repeat visit may be scheduled. After all procedures of the Weekly Home Visit have been completed, a reminder will be given about the next scheduled study visit and how to complete the Weekly Diary Card at home. HIV counseling and testing will not be systematically provided by study staff during the Weekly Home Visit but may be offered as clinically appropriate.

It should be noted that as an event-driven trial, analysis of the primary study endpoint can take place when 117 cases of severe rotavirus gastroenteritis are accrued. This intermediate analysis may be triggered before all participants reach their second year of age, but weekly follow-up for each child will continue in the same manner until all participants reach 2 years of age.

In the immunogenicity sub-cohort, participating pregnant women will receive a facility-based prenatal visit at 20 and 30 weeks gestation. At each prenatal visit, maternal anthropometry (weight, height, mid upper arm circumference), blood pressure and hemoglobin will be assessed as part of the standard prenatal check, and a blood, urine and stool sample will be collected. Following a live birth in the immunogenicity sub-cohort, infant follow up will continue with the standard Pre-randomization and Randomization Visits regardless of participation in the prenatal nutrition intervention.

Gastroenteritis definition and surveillance

Gastroenteritis will be defined as the passage of three or more looser-than-normal stools within a 24 hour period, with or without forceful vomiting. Severe rotavirus gastroenteritis will be defined using the 20-point Vesikari scale (63), where an episode of gastroenteritis with a score of 11 or more is considered as severe. Secondary analyses will consider severe gastroenteritis defined clinically as an episode of gastroenteritis that needed overnight treatment in hospital and/or rehydration therapy equivalent to the WHO Plan B (oral rehydration therapy) or Plan C (intravenous rehydration therapy) in health facility. A gastroenteritis episode will be considered to be caused by rotavirus if a rotavirus strain is identified in a stool sample collected at or before 7 days after the end of symptoms. Gastroenteritis episodes will be classified as two separate episodes if there is an interval of 5 or more consecutive, diarrhea free days between the episodes.

Cases of gastroenteritis episodes will be captured through facility- and home-based surveillance from the moment the first dose of vaccine or placebo is administered until 2 years of age. Through facility-based surveillance, one study nurse or physician will be assigned to each health facility able to receive children for gastroenteritis serving the study population of Madarounfa (e.g. 1 hospital, 5 health centers and 12 health posts). Surveillance at all levels of health facilities has been selected for case detection based on available evidence regarding health care seeking behavior in Maradi. In 2009, we conducted a household survey including 2940 children < 5 years of age in 4 districts of Maradi to assess health care utilization practices for the treatment of childhood diarrhea (64). The survey found 37% (95% CI: 34-40%) of children had at least one episode of diarrhea in the previous 1 month and over 70% of cases were managed at a health structure. Lower level health facilities were found to be more frequently visited than hospital for diarrhea management, with 54% of severe diarrhea cases presenting to a health center, 26% to a health post and 11% to a hospital. This evidence suggests that hospital-based case detection alone would be inadequate in this context, and we therefore propose inclusion of health facilities at all levels, in combination with home-based surveillance, for complete case detection.

To encourage presentation at health facilities, caregivers will be informed about the signs and symptoms of gastroenteritis and will be asked to seek care at a local facility if any episode of gastroenteritis is suspected. Compensation for travel to the local facility will be provided for each visit made. Caregivers will be instructed to show the study identification card provided at randomization at the time of presentation to the facility that will identify the infant as a participant in the study. At the facility, the study staff assigned to that facility will collect a stool sample if three or more looser-than-normal stools per day is identified within a recall period of 7 days, record history of symptoms and medical intervention received for the current illness through caregiver interview, and conduct a clinical examination to document physical signs and clinical indicators of disease severity. Documentation of clinical indicators will include temperature, the number and duration of vomiting and diarrhea episodes, dehydration status as defined by WHO (65), overnight hospitalization and treatment received in order to assign a Vesikari score. If stool is not available, a rectal swab will be taken. After discharge from the

facility, all continuing gastroenteritis episodes will be followed at home by a nurse assistant on a daily basis until resolution (defined as ≥ 5 consecutive, diarrhea free days). If 2 consecutive diarrhea -free days are not recorded within 7 days of discharge, the nurse assistant will refer the child back to a study facility for follow up. Community sensitization and outreach with community leaders, traditional medicine providers and others will be continuously reinforced to facilitate referral of sick children to health facilities throughout the study period.

A home-based surveillance system will be used to identify episodes of gastroenteritis for which a caregiver chooses not to present to a health facility. Caregivers will be advised to immediately inform their community health agent whenever there are three or more looser-than-normal stools within 24 hours. Caregiver communication of gastroenteritis episodes to community health agents will be reinforced through continued education intended to maintain caregiver awareness and interest in study activities and gastroenteritis surveillance. Episodes not immediately reported to the community health agent will be captured during review of the Weekly Diary Card at the Weekly Home Visit with caregivers. Once an episode of gastroenteritis is identified by a community health agent (either through caregiver notification intermediate to a scheduled Weekly Home Visit or during a scheduled Weekly Home Visit), it will be referred to a nurse assistant, who will monitor the episode with daily home visits until resolution (defined as ≥ 5 consecutive, diarrhea -free days). At each visit, the nurse assistant will complete a standardized Case Surveillance Card with the caregiver to collect information on the number of stools, vomiting episodes, temperature, dehydration status, medication or rehydration administered, and any medical attention sought (defined as medical personnel or facility contact, advice, visit or admission). If an episode of gastroenteritis is identified within 7 days of a home visit, a stool sample will be collected and transported to the Maradi laboratory on the same day. If any gastroenteritis episode is found to require medical attention, the nurse assistant will refer the child to a study physician to ensure appropriate medical care is received.

All data collected during the episode through facility- and home-based follow up will be integrated and transcribed to a standardized Gastroenteritis Surveillance CRF by study physicians after resolution of the episode.

Adverse event (AE) definition and surveillance

An AE is any untoward medical occurrence in a clinical investigation subject administered a pharmaceutical product and that does not necessarily have a causal relationship with this treatment. An AE can therefore be any unfavorable and unintended sign (including an abnormal laboratory finding), symptom, or disease temporally associated with the use of a medicinal (investigational) product, whether or not related to the medicinal (investigational) product. AEs include all events, including but not limited to fever diarrhea, vomiting, decreased appetite, -decreased activity level, otitis media, and nasopharyngitis. All adverse events will be assessed using facility- and home-based surveillance in all participants and graded for severity from the time of first dose until 28 days post-Dose 3. Caregivers will be informed about the signs and symptoms of adverse events and will be asked to seek care at a local facility in the event any adverse event is suspected and of concern. At the facility, study staff will record history of

symptoms of the current illness through caregiver interview and conduct a clinical examination to document physical signs and clinical condition.

In home-based surveillance, caregivers will be trained to complete the Weekly Diary Card including adverse events and be advised to immediately inform their community health agent when an adverse event is suspected. Events not reported to the community health agent will be captured during the Weekly Home Visit, when the community health agents will review the Weekly Diary Card and confirm information on the incidence of all adverse events and related medical interventions. If any adverse event is identified up to 28 days post-Dose 3 either through caregiver notification intermediate to a scheduled Weekly Home Visit or during a scheduled Weekly Home Visit, it will be referred to a nurse assistant, who will follow the case with daily home visits until resolution. Nurse assistants will refer all adverse events that require medical attention, progress clinically or do not resolve within 7 days to a study physician to ensure appropriate medical care is received.

Serious adverse events (SAEs) defined as any untoward medical occurrence that at any dose results in death, is life threatening, requires inpatient hospitalization or prolongation of existing hospitalization, or results in persistent or significant disability or incapacity, or any medically important event / reaction that may jeopardize the subject and may require medical or surgical intervention to prevent one of the outcomes listed above (66, 67), will be assessed in all participants from the time of first dose until 2 years of age through facility- and home-based surveillance. Caregivers will be informed about the signs of SAEs and will be asked to seek care at a local facility if any is suspected. At the facility, study staff will record history of symptoms of the current illness through caregiver interview and conduct a clinical examination to document physical signs. As with cases of gastroenteritis and adverse events, a standardized Weekly Diary Card will be completed by caregivers and reviewed by community health agents in home-based surveillance to document the incidence of any potential SAE not attended to at a health facility. SAEs will be followed up until the event resolves, stabilizes, or is otherwise explained by the Site Principal Investigator/Medical Monitor, who will determine whether the event is causally associated with vaccination.

Discontinuation criteria

Vaccinations may be discontinued, but follow-up continued, for any of the following situations:

- If subject suffers from immediate hypersensitivity reaction following vaccination
- If subject suffers from significant inter-current illness or undergoes major surgeryduring the course of the study
- If there is protocol violation
- If the participant receives a licensed rotavirus vaccine

A participant will be discontinued from follow-up if parent/guardian consent is withdrawn, or if the participant has permanently migrated or moved from the study area or is lost to follow up. Participation may also be discontinued if it is the opinion of the Site Principal Investigator

Investigator that it is not within the participant's best interest to continue vaccination or follow up.

Site Principal Investigator will discuss any potential discontinuation with the Medical Monitor and Sponsor Study Director. The Site Principal Investigator will have final authority to discontinue and resume vaccination and/or follow up of any participant.

In the event of a participant's discontinuation in study follow up, an Early Termination Visit will be performed by the Site Principal Investigator. The Early Termination Visit will include a review of address and contact details; physical examination; update of relevant medical history and intervention; review of vaccination history; and documentation of reason(s) for termination. In the event of a Serious Adverse Event leading to discontinuation, the participant will be followed until resolution of the event and/or the end of the study (whichever is earlier). A child's parent/guardian can withdraw consent for participation in the study at any time without prejudice.

After discontinuation of follow up, assessment of all endpoints will cease, and any participant discontinued from the study will not be replaced. Data collected up to the point of last contact will be included in the analysis. Participants who are discontinued due to migration or lost to follow up but later present and are willing to continue participation will continue to be followed until the end of follow-up, and all available data will be included in the analysis.

Immunogenicity sub-cohort

Immunogenicity: A total of 1320 infants (half vaccine, half placebo recipients) for whom permission is obtained will be enrolled in an "immunogenicity sub-cohort" to assess immune response to the vaccine. Venous blood samples will be collected at 4 time points at a health facility for measurement of concentrations of anti-rotavirus IgA: at inclusion, 28 days (± 7 days) Post Dose-3 and at 1 and 2 years of age (± 7 days). The sera will be tested for anti-rotavirus IgA at the Cincinnati Children's Hospital Medical Center (CCHMC), Cincinnati, Ohio, USA. Sero-conversion will be defined as ≥ 3 fold rise in serum titre of anti-rotavirus IgA from baseline to 28 days after receipt of Dose 3.

Prenatal nutrition intervention: Three prenatal nutrition supplements will be evaluated in the immunogenicity sub-cohort: 1) iron-folate (60 mg iron + 400 mcg folic acid as standard of care during pregnancy); 2) multiple micronutrients with approximately two times the Recommended Dietary Allowance of micronutrients, and 3) 40 g lipid-based nutrient supplement specifically designed for pregnant and lactating women with energy, protein, fats and multiple micronutrients. Daily supplements will be provided on a weekly basis until pregnancy outcome. In this sub-cohort, the participating pregnant women will receive a facility-based prenatal visit at 20 and 30 weeks gestation. At each prenatal visit, maternal anthropometry (weight, height,

mid upper arm circumference), blood pressure and hemoglobin will be assessed as part of the standard prenatal check, and a blood, urine and stool sample will be collected. Following a live birth in the immunogenicity sub-cohort, infant follow up will continue with the standard Pre-randomization and Randomization Visits regardless of participation in the prenatal nutrition intervention.

EPI interference: We intend to assess, in an exploratory way, any potential interference of BRV-PV on the immune responses to EPI vaccines administered concurrently at 6, 10 and 14 weeks. The 28 days (± 7 days) Post Dose-3 sera will be tested for following:

- Neutralizing antibody titers to polio virus serotypes 1, 2 and 3 (≥1:8 titers – seroprotection)
- Antibody concentrations to Diphtheria Toxoid ≥ 0.1 IU/mL (seroprotection)
- Antibody concentrations to Tetanus toxoid ≥ 0.1 IU/mL (seroprotection)
- Antibody concentrations to Pertussis expressed as GMCs
- Antibody concentrations to Hepatitis B (anti-HBs) ≥ 10 mIU/mL
- Antibody concentrations to Hib (anti-PRP antibodies) ≥ 1 mcg/mL (long term seroprotection)

Appropriate written consent will be obtained from their parents/guardians for participation in the immunogenicity sub-cohort.

Table 4. Summary of follow up and assessment schedule in efficacy cohort⁺

	Pre-randomization visit	Randomization and Dose 1 facility visit	Dose 2 facility visit	Dose 3 facility visit	Dose 3 + 28 Days facility visit	Weekly home visit	Facility visit	Year 1 and Year 2
PRE-RANDOMIZATION								
Informed consent for pre-randomization assessment	X							
Birth weight, gestational age and maternal MUAC	X							
ENROLMENT								
Eligibility evaluation		X						
Informed consent for trial participation		X						
Randomization		X						
INTERVENTION								
Vaccine		X	X	X				
Placebo		X	X	X				
ASSESSMENTS								
Socio-demographic questionnaire		X						X
Anthropometry		X	X	X	X	X*		X
Diary card for gastroenteritis, AE, SAE			X	X	X	X		X
Clinical exam and history		X	X	X	X	X	X	X
Post-immunization safety assessment		X	X	X				
Review of discontinuation criteria			X	X	X	X	X	
Stool sample						X**	X**	

⁺ Immunogenicity sub-cohort will enroll and follow women during pregnancy with the addition of two prenatal visits at 20 and 30 weeks gestation and then follow the same schedule of follow up as the efficacy cohort. Additional samples (blood, urine, stool and breast milk) will be collected from the mother and child..
*Height, weight and MUAC recorded at home every 4th weekly visit until 2 years of age.
** Stool collection in the event of gastroenteritis identified within a 7 day recall period.

Laboratory assessment

Stool. Stool samples will be collected in health facilities at the time of presentation for gastroenteritis, and at home for any gastroenteritis identified within a recall period of 7 days. A minimum of 5-10 ml of stool (if watery) or 5 gm (if semi-solid) will be collected using clean, dry, leak-proof plastic containers or, if no stool is available during the observation period, using a

rectal swab. Specimens will be transported in freezer packs at 2-8° C to the Epicentre laboratory in Maradi on the same day. Upon arrival at the Maradi laboratory, designated laboratory personnel will verify the stool container contains an adequate amount of specimen and is adequately labeled before storing at -80° C. If the sample is deemed inadequate, a request will be made to the nurse assistant for an additional sample. Rotavirus antigen in stool will be detected by enzyme immunoassay (Premier Rotaclone) by the Epicentre Maradi laboratory. All rotavirus positive stool samples will be shipped to the Cincinnati Children's Hospital Medical Center in Cincinnati, Ohio, USA for testing by reverse transcriptase PCR followed by reverse hybridization assay and sequencing to identify G and P types. The Site Principal Investigator will monitor specimen handling, storage and transport throughout the trial. Sample SOPs for enzyme immunoassay and reverse transcriptase PCR analysis are provided in Appendix B. Testing for other enteric pathogens will not be systematically conducted during the study period, though stool samples may be stored for later analysis. In the immunogenicity sub-cohort, additional stool samples from mothers (at inclusion, 20 and 30 weeks gestation, birth, 6 weeks and 6 months post-partum) and children (at birth, all Dosing Visits, 28 days post-Dose 3, 6 months of age and every 3 months thereafter) will be collected and stored at-80°C for future analysis.

Venous blood. For immunogenicity assessment, 4 ml of venous blood will be collected from 660 infants per group in the immunogenicity sub-cohort to determine the serum concentrations of anti-rotavirus IgA antibodies at the Randomization Visit, 28 days (± 7 days) after Dose 3 Visit, and at 1 and 2 years of age (± 7 days). All blood samples will be collected at the health facilities and transported on the same day in freezer packs at 2-8° C to the Epicentre laboratory in Maradi, where they will be separated into sera. Sera will be aliquoted and stored at -80°C until shipment for analysis. Samples will be shipped on dry ice to the Cincinnati Children's Hospital Medical Center in Cincinnati, Ohio, USA, where they will be analyzed for anti-rotavirus IgA by enzyme immunosorvent assay using 1% blotto. Sample SOPs for anti-rotavirus IgA by enzyme immunosorvent analysis in serum are provided in Appendix B. Anti-poliovirus types 1, 2 and 3, diphtheria-tetanus-whole cell pertussis, *Haemophilus influenzae* type b and hepatitis B antibody titres in serum will be measured. Results will be used to evaluate if sero-conversion for the EPI vaccine antibody response is non-inferior between the vaccinated and placebo groups. Maternal blood will be collected at inclusion, 20 and 30 weeks of gestation, birth, 6 weeks and 6 months post-partum and stored at -80°C future analysis, including serum micronutrient status.

Urine. In the immunogenicity sub-cohort, 1-2 mL of urine will be collected from mothers (at inclusion, 20 and 30 weeks gestation, birth) and from children (birth, all Dosing Visits, 28 days post-Dose 3, 6 months of age and every 3 months thereafter) and stored at -80°C for future analyses.

Breast milk. Immune factors in breast milk at the time of immunization have been hypothesized to contribute to lower immunogenicity of live oral rotavirus vaccines in developing countries (44, 68, 69), therefore we will assess concentrations of maternal anti-rotavirus IgA antibodies in breast milk in order to compare immunogenicity by level of maternal

anti-rotavirus IgA concentration. Breastfeeding mothers of children included in the immunogenicity sub-cohort will be asked to provide breast milk samples at brith, 6 weeks and 6 months post-partum. Samples of 5 to 10 ml will be requested in order to measure concentrations of maternal anti-rotavirus IgA antibodies by enzyme immunosorvent assay. Samples will be stored at -80°C until shipment to Cincinnati Children's Hospital Medical Center in Cincinnati, Ohio, USA for analysis.

Participant retention

Once an infant is randomized, the study team will make every reasonable effort to ensure administration of the study intervention as per protocol and continue follow-up of the infant for the entire study period. It is projected that the annual rate of loss-to-follow-up will be 10%. The sponsor will be responsible for developing study SOPs to achieve this level of follow-up.

The Pre-randomization Visit is organized to allow caregivers adequate time to consider their participation in the trial and prepare any questions they may have, thereby reducing the number of defaulters once randomization has occurred. At each household contact, study staff will provide a reminder of the next visit, and if a child is ever not present at the time of a Weekly Home Visit, the community health agent can contact neighbors or relatives in the area to ascertain expected time/day of return so that a repeat visit may be scheduled. In addition, study teams will maintain community interest in the study through periodic sensitization of community leaders and provide feedback to caregivers on child growth and development. Preventive interventions (e.g. soap, bed nets) will be regularly provided as in-kind motivation to participating households (see Reimbursement below).

Standard care

During regular study visits, study staff will verify previous receipt of any EPI vaccine through review of the infant's vaccination card, and as needed, refer study participants to the local health authority for administration of routine EPI vaccinations and vitamin A supplementation free of charge. Vaccines to be provided include: Bacillus Calmette-Guérin (birth); oral poliovirus vaccine (birth, 6, 10 and 14 weeks); combined pentavalent vaccine including diphtheria-tetanus-whole cell pertussis, *Haemophilus influenzae* type b and hepatitis B (6, 10, and 14 weeks); pneumococcal conjugate vaccine (PCV13) (6, 10, and 14 weeks); measles (9 and 16 months); and yellow fever (9 months). Vitamin A supplementation is recommended at 6, 12 and 18 months of age. Study staff will review vaccination and Vitamin A history at 9 months of age and directly administer any missing doses at that time to ensure complete coverage among study participants. For all other medical intervention to be provided during the study period, study children will be referred to study staff for management free of charge and in accordance with GCP.

Sample size

Assuming a 2% attack rate of severe rotavirus gastroenteritis, a 50% true vaccine efficacy and a 20% participant non-assessibility (including withdrawal and loss to follow up), the study will enroll 3885 children per group (total n = 7770) to have at least 90% power to detect a vaccine efficacy with a lower 95% confidence interval bound greater than 0% (70). Under these assumptions, a sample size of 7,770 participants will result in 117 cases of severe rotavirus gastroenteritis (78 unvaccinated and 39 vaccinated) required to fulfill the primary study objective and trigger an intermediate analysis.

Assuming a sero-conversion rate of 30% in the placebo group, 20% non-assessibility (including withdrawal and loss to follow up) and 30% exclusion due to detection of rotavirus disease between vaccine doses, we will assess immunogenicity in 660 children per group to have at least 90% power to detect a 20% difference in the proportion of children that sero-convert (71). To address the secondary objective to study the potential effect of prenatal nutritional supplementation on the immunogenicity of BRV-PV, the 660 children (n = 220 per prenatal nutritional intervention) receiving vaccine in the immunogenicity sub-cohort will provide > 80% power to detect 20% difference in the proportion of children that sero-convert between nutritional interventions, assuming a sero-conversion rate of 30% among those receiving iron-folic acid and 20% non-assessibility. Assuming a standard deviation of birth weight of 400 g and 20% non-assessibility, we will include a minimum of 1000 women receiving each prenatal nutritional intervention to provide > 80% power to detect a difference of 70g in the secondary endpoint of birth weight, allowing for study of interaction with the prenatal nutritional intervention and maternal infection (assumed 25% prevalence at inclusion).

Data analysis

The trial is event-driven, therefore an intermediate per-protocol analysis of vaccine efficacy will be triggered by the occurrence of 117 cases of severe rotavirus gastroenteritis identified from 28 days after Dose 3 among children receiving the full vaccine/placebo regimen. In the event that 117 cases are not accrued by the time all infants reach their second year of age, the analysis of efficacy will be conducted based on all available data.

The primary per-protocol analysis of vaccine efficacy will include children who received three doses of vaccine/placebo as per randomization on schedule. The primary per protocol analysis will exclude participants with a laboratory confirmed rotavirus disease from randomization to 28 days post-Dose 3 of vaccine or placebo. Infants who have at least one episode of severe rotavirus gastroenteritis during the period from 28 days after the last dose until 117 cases are accrued, or until all participating infants reach 2 years of age if 117 cases are not attained, will be considered as having achieved the primary outcome. Only gastroenteritis episodes in which rotavirus is identified in a stool specimen will be included as an event in the primary efficacy analysis. Gastroenteritis episodes that cannot be classified as rotavirus gastroenteritis or non-rotavirus gastroenteritis with certainty because of incomplete data will be considered as not having achieved the primary outcome. For participants with more than one episode of severe rotavirus gastroenteritis, only the first episode will be counted towards the primary endpoint.

Vaccine efficacy with 95% confidence interval will be calculated as $(1-IR_1/IR_0)*100$, where IR_1 is the person-time incidence rate in the vaccinated group and IR_0 is the person-time incidence rate in the placebo group. 95% confidence intervals will be derived from the exact confidence interval using the conditional binomial distribution. The cumulative hazard of a first episode of severe rotavirus gastroenteritis between groups will be estimated as a minus-log transformation of the Kaplan–Meier survival curve, with the P value calculated using the log-rank test. The proportions of infants having at least one gastroenteritis episode will be compared between groups by Fisher's exact test and expressed in terms of relative risk. The number of events prevented by 100 vaccinated infant-years will be obtained from 100 times the difference in the incidence rate; the associated confidence interval will be derived using the method of Zou and Donner (72).

Secondary analysis of efficacy will be done for efficacy against severe rotavirus gastroenteritis from 28 days post-Dose 3 to 1 year of age, from 1 to 2 years of age, and the combined period from 28 days post-Dose 3 until 2 years of age; rotavirus gastroenteritis of any severity; rotavirus gastroenteritis with a Vesikari score of ≥ 17; severe rotavirus gastroenteritis defined clinically as an episode of rotavirus gastroenteritis that needed overnight treatment in hospital and/or rehydration therapy equivalent to the WHO Plan B (oral rehydration therapy) or Plan C (intravenous rehydration therapy) in a health facility rotavirus gastroenteritis by strain [vaccine-contained and non-vaccine G and P types]; gastroenteritis of any cause; longitudinal prevalence of rotavirus gastroenteritis; hospitalization due to rotavirus gastroenteritis; and hospitalization for any reason. In strain-specific analyses of efficacy, when more than one G type is isolated for an episode, the episode will be counted in every G type category. Exploratory analyses will assess sensitivity of all analyses to the method of case detection (facility- vs. home-based surveillance).

Intention-to-treat (ITT) analyses will be done including all participants who were vaccinated with at least one dose of vaccine or placebo and follow up beginning from the time of enrollment. Sample size allowing, vaccine efficacy will be calculated among children receiving a total of 1 or 2 doses to determine whether vaccine confers protection to infants before completion of the 3-dose regimen.

The analysis of safety will be done using the ITT population, i.e. all infants who received at least one dose of the study vaccine or placebo, and include follow up from the time of enrollment until 28 days post-dose 3 (adverse events) or the end of follow up (serious adverse events). The incidence of adverse and serious adverse events will be compared between groups with the two-sided asymptotic score test for the null hypothesis of identical incidence by group.

The analysis of immunogenicity will be done on the basis of the per-protocol population for whom immunogenicity data are available in the immunogenicity sub-cohort. Participants with laboratory confirmed rotavirus disease between study doses will not be included in the analysis of immunogenicity. Sero-conversion rates 28 days following Dose 3 will be calculated with corresponding 95% confidence intervals using the binomial distribution. Differences in geometric mean titres of serum anti-rotavirus IgA between pre-Dose 1 and 28 days following

Dose 3 will be measured with 95% confidence intervals assuming a normal distribution of log-transformed means. To assess individual-level correlates of protection, Receiver Operating Characteristic (ROC) analyses will be used to estimate the IgA threshold most predictive of protection against severe rotavirus gastroenteritis. To explore potential mediating factors of immunogenicity in this setting, sero-conversion and log-transformed geometric mean infant IgA titres 28 days post-Dose 3 will be compared by prenatal nutrition intervention group and by level of maternal breast milk anti-rotavirus IgA antibodies using the chi-square and t-test. To assess the potential interference of the study vaccine with concomitant EPI immunization, the statistical difference in anti-poliovirus, diphtheria-tetanus-whole cell pertussis, *Haemophilus influenzae* type b and hepatitis B antibody serum titres 28 days post-Dose 3 will be compared by study intervention among those receiving concomitant study vaccine and EPI administration using the chi-square test.

There are numerous risk factors for the primary endpoint which will be measured at the time of randomization, including medical history, birth weight, breastfeeding practice, and indicators of socioeconomic status. Randomization ensures that, on average, study groups will be balanced with respect to all of these risk factors, but we realize that this may not be true in any given randomized study. Thus, we will collect additional background data and compare baseline characteristics between groups to identify any significant independent imbalances. Secondary analyses will assess the group effect after adjusting for the risk factors associated with imbalances at the $P < 0.20$ significance level.

This study makes no pre-specified hypotheses regarding *a priori* effect modifiers and is therefore not powered to detect any effect modification which may occur. We acknowledge that unless there is strong modification of an observed intervention effect, the power of our study to detect effect modification will be low. However in analyses for all endpoints, we will consider whether vaccine effects are modified by the following baseline characteristics: child sex; breastfeeding practice (exclusive / partial / none); gestational age (± 37 weeks); birth weight (± 2500 g) ; breast milk IgA antibody concentration; child serum anti-rotavirus IgA concentration at Dose 1; season of inclusion; concomitant administration of EPI; and child anthropometry. To assess the statistical significance of each interaction, we will use the Wald test for risk-ratio homogeneity in the risk analyses.

All P values will be 2-sided with $P < 0.05$ considered statistically significant. No adjustment for multiple comparisons will be made as this study considers a single primary endpoint, vaccine efficacy. All other analyses of secondary endpoints would be best regarded as exploratory, and any significant findings for these endpoints would need to be confirmed. Missing data will be assumed to be missing at random. Sensitivity analyses will be conducted to assess the robustness of trial results under other methods for handling missing data (e.g. missing indicator, last observation carried forward). Data analysis will be conducted using SAS software (version 9.3 or higher).

Data collection, management and quality assurance

All the information required by the study protocol will be entered on standardized CRFs provided by the sponsor in French. The Site Principal Investigator/Medical Monitor will validate all CRFs for completeness and accuracy, signing and dating each to attest to his/her responsibility for the quality of all data recorded and that the data represents a complete and accurate record of each child's participation in the study. Source documents and CRFs will be maintained at the study site in a secure location to ensure confidentiality and will be available for review by the Study Monitor to ensure all collected data are consistent with the CRFs.

All validated CRFs will be double-entered, compared and verified for accuracy in Maradi, Niger. Data quality will be enforced through a variety of mechanisms, including referential data rules, valid values, range checks, and consistency checks against data already stored in the database (i.e., longitudinal checks).

A data validation plan will be prepared by the sponsor before study initiation. Errors will be detected by programs designed to detect missing data or specific errors in the data. These errors will be summarized along with detailed descriptions of the specific problem in a Data Query Report, which will be sent to the Site Principal Investigator and Medical Monitor for resolution by checking the original forms for inconsistency, checking other sources to determine the correction, and modifying the original (paper) form as appropriate. Written documentation of changes will be available via electronic logs. A complete back up of the study database will be performed twice a month; incremental data back-ups will be performed on a daily basis. The data manager will provide monthly email reports with information on missing data, missing forms, and missing visits to the sponsor. The primary sponsor alone will have full access to the data.

Timeline

The estimated total duration of the study is approximately 54 months. Given Epicentre's established field presence in Maradi and experience with rotavirus surveillance in the region, we anticipate 6 months will be used for study preparation, including development of SOPs and CRFs, ethical clearance, regulatory approvals, recruitment and training of field personnel, initiation of the community-based birth registry and facility-based surveillance system and pilot testing. Recruitment of 7770 infants will take approximately 18 months. We anticipate recruiting approximately 108 infants per week in 5 rural zones of the Madarounfa Health District [e.g. Gabi, Safo, Madarounfa, Dan Issa, and Tofa], where there were 9597 live births in 2013 (62). The primary endpoint will be achieved when 117 cases of severe rotavirus gastroenteritis are accrued, however surveillance for identification of additional cases will continue until all participating children reach two years of age. Therefore, the duration from enrollment of the first participant until the end of follow-up of the last participant enrolled will be 42 months. Analysis and write-up will be completed in the remaining 6 months after study closure.

SAFETY EVALUATION

Standard care

For the duration of the study period, all study children found needing medical intervention will be referred to study staff for management free of charge. Study staff will verify previous receipt of any EPI vaccine and vitamin A supplementation at study visits through review of the infant's vaccination card, and as needed, refer the child to the local health authority for administration free of charge. Vitamin A supplementation will be provided at 6, 12 and 18 months of age and vaccines to be provided include: Bacillus Calmette-Guérin (birth); oral poliovirus vaccine (birth, 6, 10 and 14 weeks); the combined pentavalent vaccine including diphtheria-tetanus-whole cell pertussis, *Haemophilus influenzae* type b and hepatitis B (6, 10, and 14 weeks); pneumococcal conjugate vaccine (6, 10, and 14 weeks), measles (9 and 16 months); and yellow fever (9 months). Study staff will review vitamin A supplementation and vaccination history at 9 months of age and directly administer any missing doses at that time to ensure complete coverage among study participants.

Documenting adverse events

The collection, recording, assessment and reporting of post-immunization reactions, adverse events (AEs) and serious adverse events (SAEs) represent the core activities for the safety evaluation of the vaccine. The participant's caregiver will be instructed to present at a health facility immediately, should the participant manifest any signs or symptoms they perceive as serious during the study period.

Surveillance for adverse events will be conducted from the time of the first dose to 28 days post-Dose 3 (adverse events) in all participants by trained study staff. Adverse events can include but are limited to fever, diarrhea, and vomiting, decreased appetite, decreased activity level, otitis media and nasopharyngitis. All adverse events will be graded for severity.

During the surveillance period, AEs observed by the study team or reported by the participant's caregiver spontaneously or in response to a direct question (up to 28 days post-Dose 3) will be followed on a daily basis by a nurse assistant until resolution. The diagnosis, date and time (where appropriate) of onset, outcome (e.g. resolved without sequelae; resolved with sequelae; ongoing; died; or lost to follow up/unknown); and treatment received will be recorded.

NOTE: Any identified gastroenteritis episode from randomization to 28 days post-Dose 3 will be reported as an AE. After that period, gastroenteritis will not be reported as adverse event but will be considered towards the primary efficacy endpoint. Episodes of gastroenteritis will be recorded separately from other AEs throughout the study. Hospitalization of any duration for GE will be regarded as an SAE.

Assessment of adverse event severity

The severity of all AEs/SAEs occurring during study follow up will be graded as per the clinical judgment of the study physicians, taking into account information provided by parents/guardians. Each event will be assigned to one of 4 categories (intensity grades):

- GRADE 1 Mild - No medical intervention/therapy required
- GRADE 2 Moderate - Some assistance may be needed; no or minimal medical intervention/therapy required
- GRADE 3 Severe - Some assistance usually required; medical intervention/therapy required, hospitalizations possible
- Grade 4 Life Threatening - any adverse experience that places the child, in the view of the investigator, at immediate risk of death from the reaction as it occurred. (The investigator should not grade a reaction as life-threatening if had it occurred in a more severe form then it might have caused death.)

Note: An AE that is assessed as severe should not be confused with the term SAE. Severity is a category utilized for rating the intensity of an event; both AEs and SAEs can be assessed as severe.

Documenting serious adverse events

Surveillance for serious adverse events (SAEs) will continue from the time of the first dose until 2 years of age. SAEs will be defined as an adverse event that at any dose

(1) results in death;
(2) is life threatening;
(3) requires inpatient hospitalization or prolongation of existing hospitalization;
(4) results in persistent or significant disability or incapacity (73)
(5) is medically important event / reaction that may jeopardize the subject and may require medical or surgical intervention to prevent one of the outcomes listed above

Children that die in a facility will have events before and at the time of death transcribed from hospital records to determine the cause of death. If death occurs outside of the hospital, cause of death will be established using a standard verbal autopsy form administered to determine the clinical picture and treatment received before death.

SAEs identified through facility- or home-based surveillance will be reported to the Medical Monitor and Site Principal Investigator within 24 hours of becoming aware of the SAE, whether considered to be associated with the study intervention or not. The field team will assemble

specific documentation, including medical records and other supporting documents, and will record on the CRF: type of SAE; description of event with time of onset in relation to vaccination and severity; and any medical actions taken and outcome. Where applicable, hospital records and verbal autopsies should be obtained. The dossier will be submitted to the Site Principal Investigator/Medical Monitor for adjudication and determination whether the cases were vaccine-related. All SAEs will be followed up until the event resolves, stabilizes, or is otherwise explained. If an SAE remains unresolved at the time of study closure, a clinical assessment will be made by the Site Principal Investigator/Medical Monitor and sponsor to determine if continued follow up of the SAE is warranted. All relevant field personnel will be appropriately trained in the reporting and treatment of serious adverse events according to GCP. The DSMB and the National Ethical Committee of Niger will receive a quarterly status report including all notified SAEs. Copies of each report and documentation of IRB notification will be kept in the Trial Master File by the Site Principal Investigator/Medical Monitor.

HIV status may be made known to the Site Principal Investigator/Medical Monitor through review of existing medical records during assessment of SAE causality. If the HIV status of an infant experiencing a SAE is unknown but deemed necessary for appropriate clinical management of the SAE, HIV counseling and testing may be recommended by the Site Principal Investigator/Medical Monitor at the time of SAE documentation.

Intussusception risks, assessment and management

In 1998, the rotavirus vaccine, RotaShield, was recommended for use in the United States. However, within less than a year and more than 500,000 children vaccinated, RotaShield was found to cause a transient increased risk of intussusception (estimated to occur in 1 child in 10,000) in the first 10 days after initial vaccination. RotaShield was subsequently withdrawn from the market before detailed public discussion of the risks and benefits surrounding its use (74, 75). Post-licensure studies of the 2 second-generation rotavirus vaccines currently WHO-prequalified (RotaTeq and Rotarix) have monitored the risk of intussusception in the United States, Latin America, Mexico and some countries in sub-Saharan Africa. These studies suggest that all orally administered live rotavirus vaccines carry some detectable risk of intussusception (e.g. the risks associated with Rotashield are not unique) but the risk of intussusception seems to be small (76). The Global Advisory Committee on Vaccine Safety (GACVS), an expert clinical and scientific advisory body, has concluded that currently available rotavirus vaccines continue to exhibit a good safety profile but may be associated with an increased (up to 6-fold) risk of intussusception after the first dose of vaccine in some populations (77). The levels of risk observed are substantially less than those observed with the previous vaccine, RotaShield, and the benefits of rotavirus vaccination to all infants are thought to exceed the risks, particularly in developing countries with moderate and high mortality from rotavirus disease. Active surveillance of intussusception in African and Asian countries that plan to introduce rotavirus vaccines is warranted to eventually provide additional benefit–risk information related to rotavirus vaccination (77).

A definitive diagnosis of intussusception is based on the demonstration of invagination of the intestine on contrast enema (air or liquid), ultrasound or surgery. However, a clinical case definition for the diagnosis of acute intussusception in infants and children has been developed following recommendations of a WHO consultation and through consensus of the Brighton Collaboration Intussusception Working Group (78). This definition provides a case definition that is suitable for use in studies conducted in different geographical regions with different health care facilities and resources, and has been validated in a developed and developing country setting (79). The Brighton clinical case definition for intussusception has been endorsed by the CIOMS/WHO Working Group on Vaccine Pharmacovigilance.

Parents/guardians and study staff will be trained on screening tools to promptly identify and assess any suspected cases of intussusception, including bloody stools, continuous vomiting, abdominal distension and/or abdominal “lumps”. Children presenting with any suspected symptoms or signs at the time of a home visit or presentation to a study facility will be promptly referred to the Regional Reference Hospital of the Region of Maradi, where radiological and clinical examination will be performed before evaluating the risks and benefits of surgical intervention. All subsequent surgical interventions to resolve the invagination will be performed on site. Any patient deemed complicated by the attending physician based on their clinical judgment will be referred to the Regional Hospital of Zinder for management. Resolved cases of intussusception will be followed with home visits 2, 6 and 14 days after discharge from hospital.

This study will use the clinical case definition of intussusception from the Brighton Collaboration Working Group on Intussusception (78). All cases of suspected or confirmed intussusception identified among children ≤24 months at the time of diagnosis will be analyzed by the Site Principal Investigator/Medical Monitor following WHO guidance (80). The Site Principal Investigator/Medical Monitor will define cases of intussusception as Level 1, 2, or 3 according to the certainty of the diagnosis and the Brighton Collaboration clinical case definition. A case will be considered a potential rotavirus vaccine-related intussusception case if a rotavirus vaccine was received prior to the episode of intussusception. Cases for which there is insufficient information to establish the diagnosis according to the Brighton Collaboration clinical case definition will be classified as an unconfirmed case of intussusception and analyzed accordingly with other serious adverse events. Cases for which a diagnosis of intussusception is excluded on the basis of clinical assessment and/or appropriate investigations defined in the Brighton Collaboration clinical case definition will also be considered as another potential serious adverse event.

Assessment of causality

All AEs from the time of first vaccination to 28 days post-Dose 3 and all SAEs until 2 years of age will be assessed for a causality relationship with the study product by the Site Principal Investigator / designee. Every effort will be made by the study team to explain each event and assess its causal relationship, if any, to administration of the study vaccine or placebo. Appropriate medical judgment will be used to determine the causal relationship, considering all

relevant factors including the pattern of reaction, temporal relationship, re-challenge, biological plausibility, and confounding factors such as concomitant medication, concomitant disease and relevant history.

The likelihood of the relationship of the event to the investigational product will be recorded as follows:

> **Related,** when there is a reasonable possibility that the investigational product contributed to the event.
>
> **Unrelated,** when administration of the investigational product is not suspected to have contributed to the event (73).

All post-immunization reactions (e.g. those occurring within 30 minutes of administration) will be considered related to vaccination. All AEs, SAEs, intussusception cases will be assessed for a causality relationship with the study product by the Site Principal Investigator/Medical Monitor. Hospitalization related to a pre-existing condition which did not increase in severity or frequency following initiation of the study, or for routine clinical procedures (including hospitalization for "social" reasons) that are not the result of an SAE, will be recorded on the CRF but not considered related to the vaccine.

Dose modification

No modification of the study intervention dose is allowed. Participants who experience unacceptable adverse events attributed to the study vaccine should not receive further vaccination and should be treated under the Site Principal Investigator's discretion.

Data and Safety Monitoring Board

An independent Data and Safety Monitoring Board (DSMB) has been established prior to initiating the study and is composed of 5 experts in operational, medical, and biostatistical aspects of clinical trials, including Dr. Jacqueline Deen, Chair (Menzies School of Public Health), Dr. Irène Adehossi (Ministry of Health Niger), Dr. Nael Lapidus (Université Paris VI), Dr. Milagritos D. Tapia (University of Maryland), and Dr. Hamadou Ousseini Adamou (World Health Organization). All members of the DSMB will be completely uninvolved in the running of the trial and cannot be unfairly influenced by people or institutions involved in the trial. An initial safety review is planned after the first 1000 infants complete the 28-day post-Dose 3 period, with subsequent reviews every 6 months thereafter. Each meeting will include an administrative review to assess accrual, retention, and the progress of the study, as well as quarterly safety reviews including any serious adverse events. Guidelines for trial review and modification, including statistical and non-statistical criteria for early stopping or modification based on quarterly safety reviews, will be determined at the first DSMB meeting and made available upon request through the study sponsor.

The DSMB will be provided with the following summary of blinded accruing safety data on a quarterly basis:

- Accrual and participant status data with regard to completion/discontinuation of study vaccinations
- Summaries of adverse event data by severity grade and duration
- Reported SAEs, including cases of intussusception, hospitalization and deaths

DSMB reviews will be summarized with recommendations to the sponsor, including recommendations regarding safety concerns and if the study should continue without change, be modified, or terminated. The DSMB will discuss potential stopping or any modification to the trial with the Scientific Committee and sponsor. The sponsor will have final authority to stop or modify the trial for any reason.

MONITORING AND AUDITING

The sponsor will permit trial-related monitoring, audits, Institutional Review Board review, and regulatory inspection(s), providing direct access to source data and documents.

A qualified and appropriately GCP-trained Study Monitor will be designated by the sponsor to carefully monitor all aspects of the study.

The Study Monitor will perform an initial audit before the start of the study to ensure all necessary tools and supports are in place for study implementation in accordance with GCP. During the project period, the Study Monitor will routinely contact study sites and perform on-site visits to inspect facilities and documentation, observe performance of study procedures, discuss the protocol in detail and identify and clarify any areas of weakness. The extent, nature and frequency of site visits during the project period will be based on considerations of study objectives, study design and complexity, and enrollment rate. Periodicity and nature of monitoring activities will be described in the Monitoring Plan, with a minimum of 3 formal audits (e.g. study start, 6 months after first enrollment and study closure).

Monitoring will be conducted according to ICH-GCP guidelines and study SOPs. The Study Monitor will have access to all records necessary to ensure the integrity/validity of the recorded data and will periodically review the progress of the study. During site visits and contacts, the Study Monitor will specifically:

1. Check and assess the progress of the study
2. Review study data collected
3. Perform source data verification to verify compliance with study SOPs, including adherence to inclusion/exclusion criteria, dosing schedule, and recording of concomitant medications
4. Verify correct storage, distribution and inventory of study products
5. Verify compliance with human subjects protection and research guidelines, including confidentiality procedures and informed consent process
6. Identify any issues and discuss possibilities to address their resolution with the Sponsor and Site Principal Investigators

This will be done in order to verify that:

1. The data are authentic, accurate and complete
2. The safety and rights of participants are being protected
3. The study is conducted in accordance with the approved protocol (and any subsequent amendment), GCP and all applicable regulatory requirements

Representatives of the Scientific Committee and DSMB authorized by the sponsors may accompany the Study Monitor during site visits to conduct independent audits as needed and with due consideration to relevant security issues. The processes to be reviewed can relate to participant enrolment, consent, eligibility, and allocation to study groups; adherence to trial

interventions and policies to protect participants, including reporting of harms; completeness, accuracy, and timeliness of data collection; and adherence to the International Conference on Harmonization GCP.

ETHICAL CONSIDERATIONS

Summary of known and potential risks

There is no data on the safety of BRV-PV in sub-Saharan Africa. However, based on documentation of safety of the vaccine in other settings, the risks associated with the use of the vaccine, placebo and various other study procedures proposed in this trial are expected to be minimal. All clinical and immunization procedures (oral vaccine administration and collection of venous blood, urine, stool and breast milk) will be performed by adequately trained and experienced personnel under regular supervision to minimize any risk or discomfort to participants. There is a very small risk of anal/rectal area skin abrasion while taking a swab from the rectal area. Additionally, there is also a small risk associated with phlebotomy for participants who are requested to give a blood sample. This may include pain, redness and, very rarely, local infection at the phlebotomy area.

In 1998, the rotavirus vaccine, RotaShield, was recommended for use in the United States. However, within less than a year, it was found to cause a transient increased risk of intussusception (estimated to occur in 1 child in 10,000) in the first 10 days after initial vaccination. Intussusception is a rare event, and rates of intussusception vary between countries and populations, as the rotavirus vaccine–intussusception association may be modified by environmental or genetic factors that differ between populations. Factors that are hypothesized to affect risk of intussusception or the immune response to rotavirus vaccines—including differences in infant diet, breastfeeding, concomitant administration of oral poliovirus vaccine vs. inactivated poliovirus vaccine, and maternal antibody levels—may also contribute to the variation in intussusception risk by country (44, 47). Given biological differences between the various rotavirus vaccine strains, including rates of intestinal vaccine virus replication and shedding in fecal specimens, any potential risk of intussusception may also vary between vaccine formulations.

Post-licensure studies of the 2 second-generation rotavirus vaccines currently WHO-prequalified (RotaTeq and Rotarix) have monitored the risk of intussusception in North America, Latin America, and sub-Saharan Africa. With almost 800,000 doses of pentavalent rotavirus vaccine delivered, these analyses found that any orally administered live rotavirus vaccine carries some detectable risk of intussusception but conclude that the risk of intussusception is small: an excess risk of 1 intussusception event per 65,287 RV5 vaccinees following dose 1 can be reliably excluded (81). Based on available evidence, the benefits of rotavirus vaccination to all infants greatly exceed any potential low-level risk for intussusception, particularly in countries with moderate and high mortality from rotavirus disease.

Risk minimization and benefits

All personnel involved in taking biological samples are trained health care personnel, who will be provided with additional training to avoid or minimize the possibility of any unplanned side

effects of these procedures. The trial will be conducted in compliance with protocol, GCP, and the applicable regulatory requirements. Sterile techniques and disposable sterile needles and syringes will be utilized to obtain blood.

The direct benefit individual participants may expect from participating in this study is regularly seeing a member of the study team and the assurance of the best available medical care with close and regular surveillance. Recipients of the vaccine may also potentially benefit from the probable protective effect of the vaccine against severe rotavirus gastroenteritis. At the population-level, an important benefit of obtaining data on the efficacy of the BRV-PV vaccine, if proven protective, is the vaccine potentially being made available for a larger population of children in Niger.

Informed consent

Information about the study aims, procedures and informed consent process will be provided to community leaders in all study villages before recruitment begins. Informed consent will be obtained from each participant’s parent/guardian prior to any study-related screening procedures being performed on the participant. The informed consent process will give individuals all of the relevant information they need to decide whether to participate. The consent forms to be reviewed with the parent/guardian will include information on the purpose of the study, the study intervention, procedures to be followed and the risks and benefits of participation. All informed consent procedures and documents will be in the local language.

Informed consent will be sought at 2 times during the main efficacy study. During the Pre-randomization Visit (at birth), oral informed consent will be sought by a nurse supervisor to allow recording of maternal MUAC, as well as infant birth weight and gestational age at birth. At this time, the study nurse will play a standard audio recording in the local language to present details of the full study with a parent/guardian and a description of procedures to be conducted that day. The nurse supervisor will record the subsequent discussion with the parent/guardian including all questions and answers, and if granted, confirmation that the parent/guardian agrees to participate in the Pre-randomization activities. The consent audio recording will serve as record that the oral consenting process was fair, unbiased, and that the subject had a chance to ask questions and received satisfactory responses. The nurse supervisor will also sign the Pre-randomization Signature Page (Appendix A.1) to attest the consent has been obtained following the procedure described in the study protocol and in accordance with GCP. The electronic audio file will be kept confidentially under electronic password protection. By providing details of the full study at the Pre-randomization Visit, we will allow the parent/guardian adequate time to consider their participation in the full trial and prepare any questions they may have for the Randomization Visit.

During the Randomization Visit, written informed consent for participation in the full study will be sought.
The informed consent process will begin with a study physician / designee describing the study protocol, procedures and potential risk and benefits to the parent/guardian using a

standardized Information Sheet (Appendix A.2). Study staff will give the parent/guardian ample opportunity to inquire about details of the study and ask any questions. Illiterate individuals will have the Information Sheet and Informed Consent Form (Appendix A.2) read to them in their native language in the presence of a literate and impartial witness. The witness will sign and date the consent form to attest the consent process appears to be fair and the parent/guardian voluntarily allows the child to be included. The study physician / designee who administered the consent procedure will sign to confirm that the consent has been obtained following the procedure described in the study protocol and in accordance with GCP. An infant will not be enrolled into the full study and given a randomization code until written informed consent for participation in the full trial is provided during the Randomization Visit.

One copy of the Informed Consent Form will be kept on file by the Site Principal Investigator/Medical Monitor for possible inspection by regulatory authorities and the sponsor. The participant's parent/guardian will receive the Information Sheet and a second copy of the signed and dated Informed Consent Form.

In the immunogenicity sub-cohort, all women of reproductive age (18-45 years) resident in villages within the sub-cohort catchment will be invited to participate. A study staff member will inform pregnant women of all pertinent aspects pertaining to the study, including study aims, methods and potential risk and benefits, and then seek informed consent for participation in writing. Materials consent will be obtained at this time to address the collection of venous blood, urine, stool and breast milk specimens for use in the specified protocol and in future unspecified research. If eligible at 6-8 weeks of age, infants of participating women will be randomized to receive three doses of vaccine or placebo and enter the main trial as part of the immunogenicity sub-cohort.

Confidentiality

Participants will be identified by a unique individual identification number that contains no personal identifiable information in all CRFs and laboratory specimens. All records that contain names or other personal identifiers, such as locator forms and informed consent forms, will be stored separately from study records identified by code number. The participant's caregiver will be informed that representatives of the sponsor, ethical committees or regulatory authorities may inspect their medical records to verify the information collected, and that all personal information made available for inspection will be handled in the strictest confidence. Participants' study information will not otherwise be released outside of the study without the written permission of the participant's caregiver. The Site Principal Investigator and Medical Monitor will maintain a personal list of participant identification numbers and names to enable records to be found at a later date in the event of a medical need. This information will be destroyed upon study completion as per local regulatory requirements. Personal identifiers will not be included in any study report.

All study records and data will be kept confidentially under lock and key and/or electronic password protection, as appropriate and in accordance with local data protection laws for 5 years. Only senior study personnel will have access to these records.

Reimbursement

There are no plans to provide monetary payment for participation in this study. However, as the study will require caregivers to visit a study facility for scheduled visits, the study will provide compensation for travel for all scheduled facility visits at the time of the visit. The study will provide a small prepared meal at the time of all scheduled and interim visits to a health facility. In addition, in recognition of the burden associated with the study requirement to be present for all home visits, the study will provide an in-kind motivation for participation and retention. This will include 3 pieces of soap each month for the duration of the study and one mosquito net at the time of randomization. Distribution of soap will take place at home upon completion of the designated Weekly Home Visit e.g. one time per month.

Storage of specimens

Stored study research samples will be labeled by a unique individual identification number code that can only be linked to the participant by senior study staff. All stored research samples will be entered into a secure database and all uses will be documented. Samples may be stored at several different laboratories in order to complete the analyses required to meet study primary, secondary and exploratory analyses.

Institutional Review Board approval

The study will be approved by the research ethics committee of the Ministry of Health Niger, the Comité de Protection des Personnes Ile-de-France, Commission d'Ethique de la Recherche sur l'Etre Humain, Hôpitaux Universitaires de Genève, the World Health Organization Research Ethics Review Committee and the Western Institutional Review Board, and will be done in accordance with the Declaration of Helsinki and ICH GCP guidelines.

Declaration of conflict of interests

The primary and secondary sponsors declare no conflict of interests. SIIL has donated the study products in kind.

STUDY ADMINISTRATION

Protocol amendments

Any modifications to the protocol which may impact the conduct of the study or may affect patient safety / benefit, including changes of study objectives, study design, patient population, sample sizes, and study procedures, will require a formal amendment to the protocol. Such amendment will be agreed upon by the Scientific Committee and sponsors and approved by the appropriate ethics committee prior to implementation. The sponsor will be responsible to notify the appropriate regulatory agencies and trial registry. Administrative changes of the protocol, including minor corrections and/or clarifications that have no effect on the way the study is to be conducted, will be agreed upon by Scientific Committee and sponsors and will be documented in a memorandum.

Protocol deviations and violations

A protocol violation is any departure from the approved protocol, trial documents or any other information relating to the conduct of the study which may affect the safety of trial participants or the study outcomes. Examples include failure to obtain informed consent (i.e. no documentary evidence) or enrolment of participants that do not meet inclusion/exclusion criteria.

A protocol deviation is any departure from the approved protocol, trial documents or any other information relating to the conduct of the trial that does not result in harm to the trial participants and does not significantly affect the study outcomes. Examples of deviations include missed Dosing Visits or a Dosing Visit date outside the study visit window or an isolated incident of a missed or incomplete study procedure. Serious or repeated protocol violations or deviations will require assessment of the root cause and implementation of corrective and preventive action plans. They may constitute grounds to interrupt the trial at a study site.

Any changes from protocol-specified procedures and study-related SOPs occurring during the conduct of the trial will be documented and reported as protocol violations or deviations. Protocol deviations and violations will be monitored by the Site Principal Investigator/Medical Monitor and immediately reported to the sponsor. The sponsor will inform reviewing ethical committees, as appropriate and in accordance with the requirements of the involved committees.

Ancillary care and insurance

In the event that a participant suffers injury attributable to participation in this study, appropriate medical management and treatment will be paid for by the study and provided by the local health authority with support from the study staff. The study sponsor will have insurance to cover non-negligent harm associated with the protocol.

Data storage and archival

The sponsor will provide the Site Principal Investigator/Medical Monitor with a Trial Master File, which will be used to file the protocol, drug accountability records, correspondence with the IRB and sponsor, and other study-related documents. The Site Principal Investigator and Medical Monitor will maintain, and store securely, complete, accurate and current study records throughout the study.

The sponsor will keep essential documents, including participant's medical records, until at least 5 years after study closure. No data will be destroyed without the permission of the sponsor.

Dissemination and authorship policy

When the clinical study report is completed, the investigators will share the summary results with local, regional and national immunization program representatives to provide results and answer any questions.

The findings from this study will also be published in a peer-reviewed scientific journal and disseminated at appropriate national and international conferences. Each paper or abstract will be submitted to the Scientific Committee for review of its appropriateness and scientific merit prior to submission. The Scientific Committee may recommend changes to the authors but the ultimate decision to submit will remain with the primary sponsor. Every attempt will be made to reduce to an absolute minimum the interval between the completion of data collection and the release of the study results. A period of 3 to 6 months is expected to compile the final results paper for an appropriate journal. Trial results will be disseminated to key stakeholders regardless of the direction or magnitude of effect.

The sponsors and Scientific Committee will determine the specific topics and numbers of publications, with rights to authorship being determined by intellectual contribution to the study design, implementation, and analysis, as is specified by most major scientific journals. Preference will be given for publication in peer-reviewed, open-access journals with appropriate readership and high impact factors.

Data sharing policy

The research data will be the property of the sponsors, though we realize that the data collected from this study may provide other investigators with the opportunity to answer scientific questions about a number of ancillary issues. Therefore, data will be made as widely and freely available as possible, in a timely fashion, while safeguarding the privacy of participants and protecting confidential data. A de-identified data set can be made available under a data sharing agreement that provides for a commitment to using the data only for research purposes and securing data using appropriate technology.

STUDY MANAGEMENT

Study sponsors

The primary sponsor will develop the study protocol, with substantial input from Serum Institute of India, Limited (SIIL), and other partners. The primary sponsor will hold the data and conduct all analyses. The final report will be written by the primary sponsor, who will have full access to the data and final responsibility for the data analysis and decision to submit for publication. SIIL will have no direct oversight, participation in field activities, DSMB meetings, or data analysis. Primary sponsor staff will independently monitor study execution at field sites. The sponsor will participate in open sessions of DSMB meetings but will not participate in closed sessions of DSMB meetings. The study will be managed by a primary sponsor-investigator who both initiates and conducts the trial. The obligations of a sponsor-investigator include both those of a sponsor and those of an investigator.

The secondary sponsor is Médecins Sans Frontières- Operational Center Geneva (MSF-OCG). MSF-OCG has agreed with the primary sponsor to act as the Primary Sponsor’s legal representative in relation to the trial site and provide funding for the trial.

Serum Institute of India, Limited

Vaccine and placebo are to be provided in kind by SIIL.

Scientific Committee

The Scientific Committee (see Appendix C for Terms of Reference and Membership) will be asked to contribute to the following activities:

- Review drafts of the trial protocol, and agree on a final version;
- Advise and agree on an Analysis Plan covering data generated by the trial;
- Reply promptly to specific queries regarding the trial methods, practical implementation, analysis and interpretation;
- Review drafts of the final trial report, and agree on a final version.

Reporting procedures of the Scientific Committee to the sponsor are as outlined in the Terms of Reference and Membership (Appendix C). The Sponsor Study Director assumes responsibility that the Scientific Committee is informed of all components of the trial protocol.

Data and Safety Monitoring Board

An independent Data and Safety Monitoring Board (DSMB) has been established prior to initiating the study and is composed of 5 experts in operational, medical, and biostatistical aspects of clinical trials. All members of the DSMB will be completely uninvolved in the running of the trial and cannot be unfairly influenced by people or institutions involved in the trial. An initial safety review is planned after the first 1000 infants complete the 28-day post-Dose 3

period, with subsequent reviews every 6 months thereafter. Each meeting will include an administrative review to assess accrual, retention, and the progress of the study, as well as quarterly safety reviews including any serious adverse events. Guidelines for trial review and modification, including statistical and non-statistical criteria for early stopping or modification based on quarterly safety reviews will be determined at the first DSMB meeting and made available upon request through the study sponsor. Reporting procedures to the study sponsor will be outlined in the DSMB charter.

Human resources

Overall study development and direction will be provided by a team of 5 investigators based in Paris and Niger (Figure 1). Rebecca Freeman Grais will serve as the Sponsor Study Director for the study. Dr. Grais is an international expert in vaccination in sub-Saharan Africa, and as the Sponsor Study Director, will be the guarantor of the trial. Sheila Isanaka, Sponsor Principal Investigator, is a recognized epidemiologist with extensive experience in conducting large-scale trials of high burden pediatric morbidities. Dr. Isanaka will be responsible for ensuring appropriate study design and procedures. Ousmane Guindo, Site Principal Investigator, Epicentre Niger is a medical doctor with extensive experience in the conduct of clinical trials in the Sahel. Emmanuel Baron, Co-investigator, General Director of Epicentre and former Medical Director of Médecins Sans Frontières-Operational Center Paris, will ensure the appropriate medical management of all study participants. Dr. Baron has over 15 years of experience in providing and managing medical care throughout sub-Saharan Africa. Celine Langendorf, Co-investigator, Epicentre, has extensive laboratory experience in sub-Saharan Africa and Asia and will ensure the rigor of all laboratory procedures.

In addition, the study will be supported a permanent GCP-trained Study Monitor based in Maradi, Niger. Through routine contact and site visits, the Study Monitor will ensure all necessary tools and supports are in place for study implementation, review collected data and perform source data verification in accordance with GCP.

All day-to-day study activities will take place in the Madarounfa Health District, Maradi, Niger, and interaction with participants will be primarily carried out by the study field team. The field team will be comprised of a Site Principal Investigator and Medical Monitor based in Maradi, and specialized health care personnel based in rural sites throughout the Madarounfa Health District and responsible for the enrollment and medical follow-up of all study participants. The Site Principal Investigator will be under the supervision of the Sponsor Principal Investigator. There will be at least weekly communication by email, teleconference or videoconference to discuss study activities and challenges with the Site Principal Investigator, Medical Monitor and Sponsor Principal Investigator.

The Site Principal Investigator will be responsible for coordination of all field worker and supervisor schedules, standardization of follow up methods and assurance that activities are conducted according to protocol. The Site Principal Investigator/Medical Monitor will be

responsible for overseeing the medical management and follow-up of all study children. The Site Principal Investigator will supervise 3 teams with the following specific roles:

- Facility medical team will include field-based and facility-based personnel. Facility-based personnel (n = at least 1 per site during normal facility operating hours) will include study physicians and study nurses. One study physician will be based in each designated Enrollment and Dosing Site and will be responsible for recruitment (including the informed consent process), administration of study vaccine and placebo and post-immunization observation, receiving children presenting to the facility with symptoms of gastroenteritis or other medical complication, and recording all CRFs. Two study nurses will also be based at each Enrollment and Dosing Site to facilitate pre-randomization and enrollment activities. In addition, one study nurse will be based in all remaining facilities in the Health District and will be responsible for receiving children presenting to the facility with symptoms of gastroenteritis or adverse events and recording pertinent medical information for transcription to standardized CRFs by the study physicians.

- Community surveillance team will include community health agents and nurse assistants. The community health agent (n = 1 per 20-25 children; 4-5 home visits per day) will maintain a population registry for their village, conduct the Weekly Home Visits to review the Weekly Diary Card with caregivers, support notification of the nurse assistant when an episode of gastroenteritis or adverse event is identified. The nurse assistant (n = 1 per 100 children; 4-5 visits per day) will follow children at home with gastroenteritis or any adverse event to collect information regarding the severity of the episode, and as appropriate stool samples. Nurse supervisors (n= 1 per 10-15 agent) will directly oversee the community surveillance team by tracking follow up logs and attending home and facility visits to ensure procedures are standardized and conducted according to protocol.

- Laboratory team, including 2 laboratory technicians and 1 laboratory supervisor, will be responsible for the preparation and storage of all biological samples collected during the study period; analysis of collected stool for the presence of rotavirus antigen by enzyme immunoassay; and preparation all rotavirus positive stool samples for shipment to the Cincinnati Children’s Hospital Medical Center in Cincinnati, Ohio, USA for further testing by reverse transcriptase PCR.

- Data management team, including 5 data entry operators and 1 Data Manager, will be responsible for the double-entry and validation of all data collected on standard CRFs.

- Study Communicator will be responsible for maintaining consistent dialogue with community leaders and study participants identify and resolve issues related to study procedures and developments.

All teams will be adequately staffed with sufficient back-up personnel to allow continuity of study activities over standard annual and sick leaves.

Training

Study staff will be trained in GCP, serious adverse event guidelines, clinical assessment of patients, completion of relevant source documents and CRFs, specimen collection and storage of samples. Initial training will be provided by the Site Principal Investigator and Medical Monitor, with support from the Study Monitor and other partners. Refresher training will be provided on a quarterly basis to all study personnel by the Site Principal Investigator and Medical Monitor. Routine audits performed by the Study Monitor will be used identify procedures that need to be strengthened and reinforced in routine refresher training.

Figure 1. Study organogram

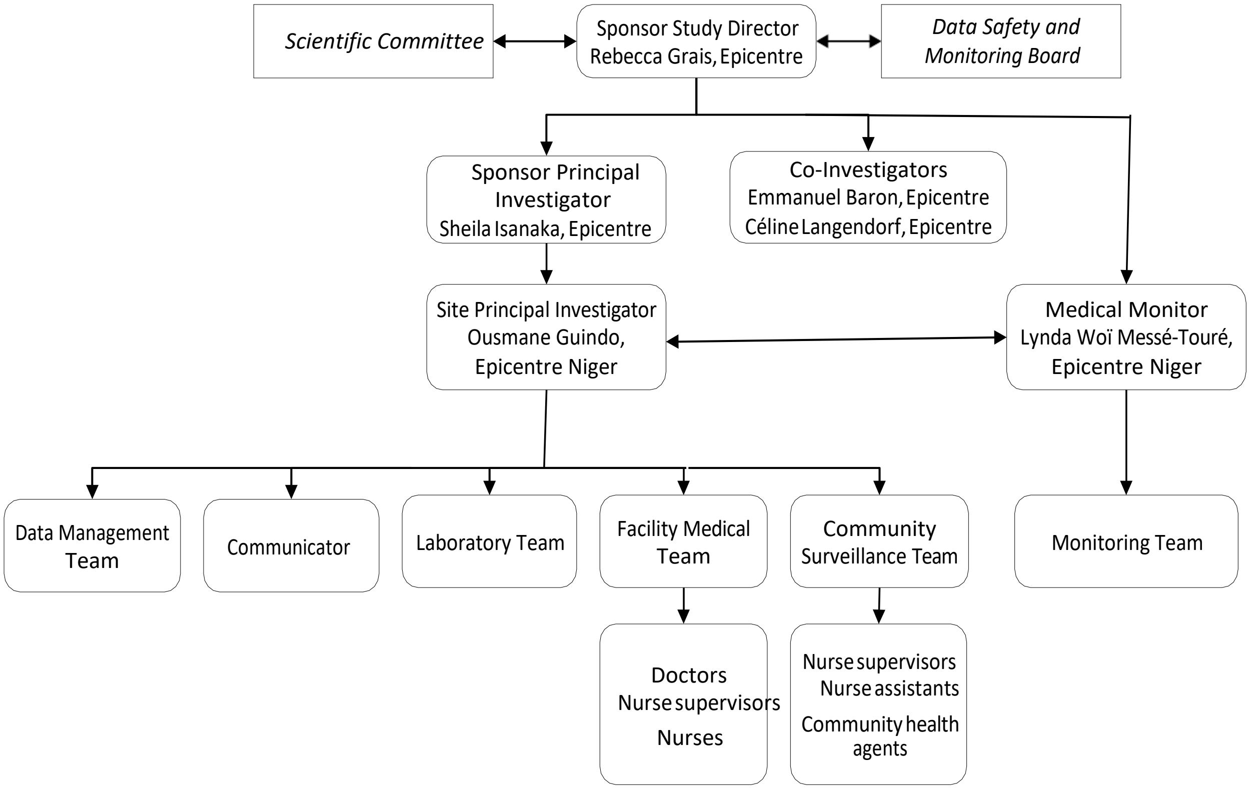

REFERENCES

1. Liu L, Johnson HL, Cousens S, Perin J, Scott S, Lawn JE, Rudan I, Campbell H, Cibulskis R, Li M, Mathers C, Black RE. Global, regional, and national causes of child mortality: an updated systematic analysis for 2010 with time trends since 2000. Lancet. 2012;379(9832):2151-61. Epub 2012/05/15. doi: 10.1016/S0140-6736(12)60560-1 S0140-6736(12)60560-1 [pii]. PubMed PMID: 22579125.
2. United Nations Children's Fund. Pneumonia and diarrhoea: tackling the deadliest diseases for the world's poorest children. . New York: United Nations Children's Fund, 2012.
3. Kosek M, Bern C, Guerrant RL. The global burden of diarrhoeal disease, as estimated from studies published between 1992 and 2000. Bull World Health Organ. 2003;81(3):197-204. Epub 2003/05/24. doi: S0042-96862003000300010 [pii]. PubMed PMID: 12764516; PubMed Central PMCID: PMC2572419.
4. United Nations Children's Fund and World Health Organization. Diarrhoea: why children are dying and what can be done. New York: United Nations Children's Fund, 2009.
5. Dewey KG, Mayers DR. Early child growth: how do nutrition and infection interact? Matern Child Nutr. 2011;7 Suppl 3:129-42. Epub 2011/10/05. doi: 10.1111/j.1740-8709.2011.00357.x. PubMed PMID: 21929641.
6. Kotloff KL, Nataro JP, Blackwelder WC, Nasrin D, Farag TH, Panchalingam S, Wu Y, Sow SO, Sur D, Breiman RF, Faruque AS, Zaidi AK, Saha D, Alonso PL, Tamboura B, Sanogo D, Onwuchekwa U, Manna B, Ramamurthy T, Kanungo S, Ochieng JB, Omore R, Oundo JO, Hossain A, Das SK, Ahmed S, Qureshi S, Quadri F, Adegbola RA, Antonio M, Hossain MJ, Akinsola A, Mandomando I, Nhampossa T, Acacio S, Biswas K, O'Reilly CE, Mintz ED, Berkeley LY, Muhsen K, Sommerfelt H, Robins-Browne RM, Levine MM. Burden and aetiology of diarrhoeal disease in infants and young children in developing countries (the Global Enteric Multicenter Study, GEMS): a prospective, case-control study. Lancet. 2013. Epub 2013/05/18. doi: S0140-6736(13)60844-2 [pii] 10.1016/S0140-6736(13)60844-2. PubMed PMID: 23680352.
7. World Health Organization. The Treatment of Diarrhoea: A manual for physicians and other senior health workers. Geneva: World Health Organization, 2005.
8. Fischer Walker CL, Black RE. Micronutrients and diarrheal disease. Clin Infect Dis. 2007;45 Suppl 1:S73-7. Epub 2007/07/14. doi: CID50040 [pii] 10.1086/518152. PubMed PMID: 17582575.
9. Santosham M, Chandran A, Fitzwater S, Fischer-Walker C, Baqui AH, Black R. Progress and barriers for the control of diarrhoeal disease. Lancet. 2010;376(9734):63-7. Epub 2010/07/09. doi: 10.1016/S0140-6736(10)60356-X S0140-6736(10)60356-X [pii]. PubMed PMID: 20609988.
10. Tate JE, Burton AH, Boschi-Pinto C, Steele AD, Duque J, Parashar UD. 2008 estimate of worldwide rotavirus-associated mortality in children younger than 5 years before the introduction of universal rotavirus vaccination programmes: a systematic review and meta-analysis. Lancet Infect Dis. 2012;12(2):136-41. Epub 2011/10/28. doi: 10.1016/S1473-3099(11)70253-5 S1473-3099(11)70253-5 [pii]. PubMed PMID: 22030330.

11. Mwenda JM, Ntoto KM, Abebe A, Enweronu-Laryea C, Amina I, McHomvu J, Kisakye A, Mpabalwani EM, Pazvakavambwa I, Armah GE, Seheri LM, Kiulia NM, Page N, Widdowson MA, Steele AD. Burden and epidemiology of rotavirus diarrhea in selected African countries: preliminary results from the African Rotavirus Surveillance Network. J Infect Dis. 2010;202 Suppl:S5-S11. Epub 2010/08/13. doi: 10.1086/653557. PubMed PMID: 20684718.
12. Sanchez-Padilla E, Grais RF, Guerin PJ, Steele AD, Burny ME, Luquero FJ. Burden of disease and circulating serotypes of rotavirus infection in sub-Saharan Africa: systematic review and meta-analysis. Lancet Infect Dis. 2009;9(9):567-76. Epub 2009/08/22. doi: 10.1016/S1473-3099(09)70179-3
S1473-3099(09)70179-3 [pii]. PubMed PMID: 19695493.
13. Santos N, Hoshino Y. Global distribution of rotavirus serotypes/genotypes and its implication for the development and implementation of an effective rotavirus vaccine. Rev Med Virol. 2005;15(1):29-56. Epub 2004/10/16. doi: 10.1002/rmv.448. PubMed PMID: 15484186.
14. Nordgren J, Nitiema LW, Sharma S, Ouermi D, Traore AS, Simpore J, Svensson L. Emergence of unusual G6P[6] rotaviruses in children, Burkina Faso, 2009-2010. Emerg Infect Dis. 2012;18(4):589-97. Epub 2012/04/04. doi: 10.3201/eid1804.110973. PubMed PMID: 22469076; PubMed Central PMCID: PMC3309693.
15. Page N, Esona M, Armah G, Nyangao J, Mwenda J, Sebunya T, Basu G, Pyndiah N, Potgieter N, Geyer A, Steele AD. Emergence and characterization of serotype G9 rotavirus strains from Africa. J Infect Dis. 2010;202 Suppl:S55-63. Epub 2010/08/13. doi: 10.1086/653551. PubMed PMID: 20684719.
16. Page NA, de Beer MC, Seheri LM, Dewar JB, Steele AD. The detection and molecular characterization of human G12 genotypes in South Africa. J Med Virol. 2009;81(1):106-13. Epub 2008/11/26. doi: 10.1002/jmv.21362. PubMed PMID: 19031449.
17. Todd S, Page NA, Duncan Steele A, Peenze I, Cunliffe NA. Rotavirus strain types circulating in Africa: Review of studies published during 1997-2006. J Infect Dis. 2010;202 Suppl:S34-42. Epub 2010/08/13. doi: 10.1086/653555. PubMed PMID: 20684715.
18. Ruiz-Palacios GM, Perez-Schael I, Velazquez FR, Abate H, Breuer T, Clemens SC, Cheuvart B, Espinoza F, Gillard P, Innis BL, Cervantes Y, Linhares AC, Lopez P, Macias-Parra M, Ortega-Barria E, Richardson V, Rivera-Medina DM, Rivera L, Salinas B, Pavia-Ruz N, Salmeron J, Ruttimann R, Tinoco JC, Rubio P, Nunez E, Guerrero ML, Yarzabal JP, Damaso S, Tornieporth N, Saez-Llorens X, Vergara RF, Vesikari T, Bouckenooghe A, Clemens R, De Vos B, O'Ryan M. Safety and efficacy of an attenuated vaccine against severe rotavirus gastroenteritis. N Engl J Med. 2006;354(1):11-22. Epub 2006/01/06. doi: 354/1/11 [pii]
10.1056/NEJMoa052434. PubMed PMID: 16394298.
19. Vesikari T, Matson DO, Dennehy P, Van Damme P, Santosham M, Rodriguez Z, Dallas MJ, Heyse JF, Goveia MG, Black SB, Shinefield HR, Christie CD, Ylitalo S, Itzler RF, Coia ML, Onorato MT, Adeyi BA, Marshall GS, Gothefors L, Campens D, Karvonen A, Watt JP, O'Brien KL, DiNubile MJ, Clark HF, Boslego JW, Offit PA, Heaton PM. Safety and efficacy

of a pentavalent human-bovine (WC3) reassortant rotavirus vaccine. N Engl J Med. 2006;354(1):23-33. Epub 2006/01/06. doi: 354/1/23 [pii]
10.1056/NEJMoa052664. PubMed PMID: 16394299.

20. Buttery JP, Lambert SB, Grimwood K, Nissen MD, Field EJ, Macartney KK, Akikusa JD, Kelly JJ, Kirkwood CD. Reduction in rotavirus-associated acute gastroenteritis following introduction of rotavirus vaccine into Australia's National Childhood vaccine schedule. Pediatr Infect Dis J. 2011;30(1 Suppl):S25-9. Epub 2011/01/13. doi: 10.1097/INF.0b013e3181fefdee
00006454-201101001-00006 [pii]. PubMed PMID: 21183837.

21. do Carmo GM, Yen C, Cortes J, Siqueira AA, de Oliveira WK, Cortez-Escalante JJ, Lopman B, Flannery B, de Oliveira LH, Carmo EH, Patel M. Decline in diarrhea mortality and admissions after routine childhood rotavirus immunization in Brazil: a time-series analysis. PLoS Med. 2011;8(4):e1001024. Epub 2011/04/29. doi: 10.1371/journal.pmed.1001024. PubMed PMID: 21526228; PubMed Central PMCID: PMC3079643.

22. Patel MM, Glass R, Desai R, Tate JE, Parashar UD. Fulfilling the promise of rotavirus vaccines: how far have we come since licensure? Lancet Infect Dis. 2012;12(7):561-70. Epub 2012/06/30. doi: 10.1016/S1473-3099(12)70029-4
S1473-3099(12)70029-4 [pii]. PubMed PMID: 22742639.

23. Richardson V, Parashar U, Patel M. Childhood diarrhea deaths after rotavirus vaccination in Mexico. N Engl J Med. 2011;365(8):772-3. Epub 2011/08/26. doi: 10.1056/NEJMc1100062. PubMed PMID: 21864191.

24. Tate JE, Mutuc JD, Panozzo CA, Payne DC, Cortese MM, Cortes JE, Yen C, Esposito DH, Lopman BA, Patel MM, Parashar UD. Sustained decline in rotavirus detections in the United States following the introduction of rotavirus vaccine in 2006. Pediatr Infect DisJ. 2011;30(1 Suppl):S30-4. Epub 2011/01/12. doi: 10.1097/INF.0b013e3181ffe3eb
00006454-201101001-00007 [pii]. PubMed PMID: 21183838.

25. Armah GE, Sow SO, Breiman RF, Dallas MJ, Tapia MD, Feikin DR, Binka FN, Steele AD, Laserson KF, Ansah NA, Levine MM, Lewis K, Coia ML, Attah-Poku M, Ojwando J, Rivers SB, Victor JC, Nyambane G, Hodgson A, Schodel F, Ciarlet M, Neuzil KM. Efficacy of pentavalent rotavirus vaccine against severe rotavirus gastroenteritis in infants in developing countries in sub-Saharan Africa: a randomised, double-blind, placebo-controlled trial. Lancet. 2010;376(9741):606-14. Epub 2010/08/10. doi: 10.1016/S0140-6736(10)60889-6
S0140-6736(10)60889-6 [pii]. PubMed PMID: 20692030.

26. Madhi SA, Cunliffe NA, Steele D, Witte D, Kirsten M, Louw C, Ngwira B, Victor JC, Gillard PH, Cheuvart BB, Han HH, Neuzil KM. Effect of human rotavirus vaccine on severe diarrhea in African infants. N Engl J Med. 2010;362(4):289-98. Epub 2010/01/29. doi: 10.1056/NEJMoa0904797
362/4/289 [pii]. PubMed PMID: 20107214.

27. Zaman K, Dang DA, Victor JC, Shin S, Yunus M, Dallas MJ, Podder G, Vu DT, Le TP, Luby SP, Le HT, Coia ML, Lewis K, Rivers SB, Sack DA, Schodel F, Steele AD, Neuzil KM, Ciarlet M. Efficacy of pentavalent rotavirus vaccine against severe rotavirus gastroenteritis in infants in developing countries in Asia: a randomised, double-blind, placebo-controlled

trial. Lancet. 2010;376(9741):615-23. Epub 2010/08/10. doi: 10.1016/S0140-6736(10)60755-6
S0140-6736(10)60755-6 [pii]. PubMed PMID: 20692031.

28. Rotavirus vaccines:an update. Wkly Epidemiol Rec. 2009;84(50):533-40. Epub 2009/12/26. PubMed PMID: 20034143.
29. Holmgren J, Svennerholm AM. Vaccines against mucosal infections. Curr Opin Immunol. 2012;24(3):343-53. Epub 2012/05/15. doi: 10.1016/j.coi.2012.03.014
S0952-7915(12)00063-5 [pii]. PubMed PMID: 22580196.
30. Lopman BA, Pitzer VE, Sarkar R, Gladstone B, Patel M, Glasser J, Gambhir M, Atchison C, Grenfell BT, Edmunds WJ, Kang G, Parashar UD. Understanding reduced rotavirus vaccine efficacy in low socio-economic settings. PLoS One. 2012;7(8):e41720. Epub 2012/08/11. doi: 10.1371/journal.pone.0041720
PONE-D-12-09574 [pii]. PubMed PMID: 22879893; PubMed Central PMCID: PMC3412858.
31. John TJ. Antibody response of infants in tropics to five doses of oral polio vaccine. Br Med J. 1976;1(6013):812. Epub 1976/04/03. PubMed PMID: 1260345; PubMed Central PMCID: PMC1639412.
32. Levine MM. Immunogenicity and efficacy of oral vaccines in developing countries: lessons from a live cholera vaccine. BMC Biol. 2010;8:129. Epub 2010/10/06. doi: 10.1186/1741-7007-8-129
1741-7007-8-129 [pii]. PubMed PMID: 20920375; PubMed Central PMCID: PMC2958895.
33. Steele AD, Neuzil KM, Cunliffe NA, Madhi SA, Bos P, Ngwira B, Witte D, Todd S, Louw C, Kirsten M, Aspinall S, Van Doorn LJ, Bouckenooghe A, Suryakiran PV, Han HH. Human rotavirus vaccine Rotarix provides protection against diverse circulating rotavirus strains in African infants: a randomized controlled trial. BMC Infect Dis. 2012;12:213. Epub 2012/09/15. doi: 10.1186/1471-2334-12-213
1471-2334-12-213 [pii]. PubMed PMID: 22974466; PubMed Central PMCID: PMC3462149.
34. Justino MC, Linhares AC, Lanzieri TM, Miranda Y, Mascarenhas JD, Abreu E, Guerra SF, Oliveira AS, da Silva VB, Sanchez N, Meyer N, Shafi F, Ortega-Barria E, Soriano-Gabarro M, Colindres RE. Effectiveness of the monovalent G1P[8] human rotavirus vaccine against hospitalization for severe G2P[4] rotavirus gastroenteritis in Belem, Brazil. Pediatr Infect Dis J. 2011;30(5):396-401. Epub 2010/12/15. doi: 10.1097/INF.0b013e3182055cc2. PubMed PMID: 21150692.
35. Snelling TL, Andrews RM, Kirkwood CD, Culvenor S, Carapetis JR. Case-control evaluation of the effectiveness of the G1P[8] human rotavirus vaccine during an outbreak of rotavirus G2P[4] infection in central Australia. Clin Infect Dis. 2011;52(2):191-9. Epub 2011/02/04. doi: 10.1093/cid/ciq101
ciq101 [pii]. PubMed PMID: 21288843.
36. Richardson V, Hernandez-Pichardo J, Quintanar-Solares M, Esparza-Aguilar M, Johnson B, Gomez-Altamirano CM, Parashar U, Patel M. Effect of rotavirus vaccination on death from childhood diarrhea in Mexico. N Engl J Med. 2010;362(4):299-305. Epub 2010/01/29. doi: 10.1056/NEJMoa0905211

362/4/299 [pii]. PubMed PMID: 20107215.
37. PATH. Country introductions of rotavirus vaccines. 2013 [July 14 2013.]. Available from: http://sites.path.org/rotavirusvaccine/rotavirus-advocacy-and-communications-toolkit/country-introduction-maps-and-list/.
38. Lee BY, Assi TM, Rajgopal J, Norman BA, Chen SI, Brown ST, Slayton RB, Kone S, Kenea H, Welling JS, Connor DL, Wateska AR, Jana A, Wiringa AE, Van Panhuis WG, Burke DS. Impact of introducing the pneumococcal and rotavirus vaccines into the routine immunization program in Niger. Am J Public Health. 2012;102(2):269-76. Epub 2011/09/24. doi: 10.2105/AJPH.2011.300218
AJPH.2011.300218 [pii]. PubMed PMID: 21940923; PubMed Central PMCID: PMC3386610.
39. Page AL, Jusot V, Mamaty A, Adamou L, Pothier J, Djibo A, Mamzo ML, Toure B, Langendorf C, Collard J, Grais RF. Multicentric surveillance of rotavirus diarrhea in urban and rural areas of Nigerin preparation.
40. Bhandari N, Sharma P, Taneja S, Kumar T, Rongsen-Chandola T, Appaiahgari MB, Mishra A, Singh S, Vrati S. A dose-escalation safety and immunogenicity study of live attenuated oral rotavirus vaccine 116E in infants: a randomized, double-blind, placebo-controlled trial. J Infect Dis. 2009;200(3):421-9. Epub 2009/06/24. doi: 10.1086/600104. PubMed PMID: 19545211.
41. ClinicalTrials.gov. Safety, Tolerability and Immunogenicity of Vaccination With Rotateq in Healthy Infants in India - Study Results [2013 August 2.]. Available from: http://clinicaltrials.gov/ct2/show/results/NCT00496054?term=rotateq+india&rank=1&sect=X4015#othr
42. GlaxoSmithKline. Clinical study register. [Accessed 2013 August 2]. Available from: http://www.gsk-clinicalstudyregister.com/index.jsp.
43. Narang A, Bose A, Pandit AN, Dutta P, Kang G, Bhattacharya SK, Datta SK, Suryakiran PV, Delem A, Han HH, Bock HL. Immunogenicity, reactogenicity and safety of human rotavirus vaccine (RIX4414) in Indian infants. Hum Vaccin. 2009;5(6):414-9. Epub 2009/03/12. doi: 8176 [pii]. PubMed PMID: 19276664.
44. Patel M, Shane AL, Parashar UD, Jiang B, Gentsch JR, Glass RI. Oral rotavirus vaccines: how well will they work where they are needed most? J Infect Dis. 2009;200 Suppl 1:S39-48. Epub 2009/10/13. doi: 10.1086/605035. PubMed PMID: 19817613; PubMed Central PMCID: PMC3673012.
45. Salinas B, Perez Schael I, Linhares AC, Ruiz Palacios GM, Guerrero ML, Yarzabal JP, Cervantes Y, Costa Clemens S, Damaso S, Hardt K, De Vos B. Evaluation of safety, immunogenicity and efficacy of an attenuated rotavirus vaccine, RIX4414: A randomized, placebo-controlled trial in Latin American infants. Pediatr Infect Dis J. 2005;24(9):807-16. Epub 2005/09/09. doi: 00006454-200509000-00014 [pii]. PubMed PMID: 16148848.
46. Sow SO, Tapia M, Haidara FC, Ciarlet M, Diallo F, Kodio M, Doumbia M, Dembele RD, Traore O, Onwuchekwa UU, Lewis KD, Victor JC, Steele AD, Neuzil KM, Kotloff KL, Levine MM. Efficacy of the oral pentavalent rotavirus vaccine in Mali. Vaccine. 2012;30 Suppl 1:A71-8. Epub 2012/05/02. doi: 10.1016/j.vaccine.2011.11.094
S0264-410X(11)01890-1 [pii]. PubMed PMID: 22520140.

47. Steele AD, De Vos B, Tumbo J, Reynders J, Scholtz F, Bos P, de Beer MC, Van der Merwe CF, Delem A. Co-administration study in South African infants of a live-attenuated oral human rotavirus vaccine (RIX4414) and poliovirus vaccines. Vaccine. 2010;28(39):6542-8. Epub 2008/09/13. doi: 10.1016/j.vaccine.2008.08.034
S0264-410X(08)01132-8 [pii]. PubMed PMID: 18786585.
48. Vesikari T, Karvonen A, Korhonen T, Espo M, Lebacq E, Forster J, Zepp F, Delem A, De Vos B. Safety and immunogenicity of RIX4414 live attenuated human rotavirus vaccine in adults, toddlers and previously uninfected infants. Vaccine. 2004;22(21-22):2836-42. Epub 2004/07/13. doi: 10.1016/j.vaccine.2004.01.044
S0264410X04000271 [pii]. PubMed PMID: 15246619.
49. Zaman K, Sack DA, Yunus M, Arifeen SE, Podder G, Azim T, Luby S, Breiman RF, Neuzil K, Datta SK, Delem A, Suryakiran PV, Bock HL. Successful co-administration of a human rotavirus and oral poliovirus vaccines in Bangladeshi infants in a 2-dose schedule at 12 and 16 weeks of age. Vaccine. 2009;27(9):1333-9. Epub 2009/01/24. doi: 10.1016/j.vaccine.2008.12.059
S0264-410X(09)00002-4 [pii]. PubMed PMID: 19162114.
50. Phua KB, Emmanuel SC, Goh P, Quak SH, Lee BW, Han HH, Ward RL, Bernstein DI, De Vos B, Bock HL. A rotavirus vaccine for infants: the Asian experience. Ann Acad Med Singapore. 2006;35(1):38-44. Epub 2006/02/14. PubMed PMID: 16470273.
51. Berkeley S, editor. Update from the GAVI Alliance (conference presentation). World Health Assembly, May 22 2013; 2013; Geneva, Switzerland.
52. WHO Expert Committee on Biological Standardization. Fifty-sixth report. Geneva, Switzerland: WHO Expert Committee on Biological Standardization; 2007.
53. United Nations Development Program. Human Development Report 2011. [Aug 7, 2013]. Available from: http://www.undp.org/content/undp/en/home/librarypage/hdr/human_developmentreport2011.html.
54. Institut National de la Statistique Ministére de l'Èconomie et des Finances and Macro International Inc. Enquete Demographique et de Sante et a Indicateurs Multiples. Niamey and Calverton: 2007.
55. Amouzou A, Habi O, Bensaid K. Reduction in child mortality in Niger: a Countdown to 2015 country case study. Lancet. 2012;380(9848):1169-78. Epub 2012/09/25. doi: 10.1016/S0140-6736(12)61376-2
S0140-6736(12)61376-2 [pii]. PubMed PMID: 22999428.
56. Institut National de la Statistique. Le Niger en Chiffres 2013. Niamey: Ministére de l'Èconomie et des Finances, 2013.
57. Ministère de la Santé Publique et de la Lutte contre les Endémies RdN. Plan de Développement Sanitaire 2005 - 2009. Niamey: Ministère de la Santé Publique, 2005.
58. Emanuel EJ, Wendler D, Grady C. What makes clinical research ethical? JAMA. 2000;283(20):2701-11. Epub 2000/05/20. doi: jsc90374 [pii]. PubMed PMID: 10819955.
59. . Ethical considerations arising from vaccine trials conducted in pediatric populations with high disease burden in developing countries. WHO / IVR Ethics Meeting; 2002; Accra, Ghana.

60. Advisory Committee on Immunization Practices. Rotavirus infections. In: Pickering LK, Baker CJ, Long SS, editors. RedBook: 2009 Report of the Committee on Infectious Diseases 28th ed. Elk Grove Village, IL: American Academy of Pediatrics; 2009. p. 576–9.
61. Ballard JL, Khoury JC, Wedig K, Wang L, Eilers-Walsman BL, Lipp R. New Ballard Score, expanded to include extremely premature infants. The Journal of pediatrics. 1991;119(3):417-23. PubMed PMID: 1880657.
62. Centre de surveillance epidemiologique. Repartition de la population par tranches d'age en 2014. District Sanitaire de Madarounfa, Region de Maradi, Ministère de la Santé Publique., 2014 Februaru 2014. Report No.
63. Lewis K. Vesikari Clinical Severity Scoring System Manual. Seattle: PATH, 2011.
64. Page AL, Hustache S, Luquero FJ, Djibo A, Manzo ML, Grais RF. Health care seeking behavior for diarrhea in children under 5 in rural Niger: results of a cross-sectional survey. BMC Public Health. 2011;11:389. Epub 2011/05/27. doi: 10.1186/1471-2458-11-389
1471-2458-11-389 [pii]. PubMed PMID: 21612640; PubMed Central PMCID: PMC3121637.
65. World Health Organization. Integrated management of childhood illness. Geneva, Switzerland: World Health Organization, 2005.
66. Bonhoeffer J, Heininger U, Kohl K, Chen RT, Duclos P, Heijbel H, Jefferson T, Loupi E. Standardized case definitions of adverse events following immunization (AEFI). Vaccine. 2004;22(5-6):547-50. Epub 2004/01/27. doi: S0264410X03005115 [pii]. PubMed PMID: 14741142.
67. Bonhoeffer J, Kohl K, Chen R, Duclos P, Heijbel H, Heininger U, Jefferson T, Loupi E. The Brighton Collaboration: addressing the need for standardized case definitions of adverse events following immunization (AEFI). Vaccine. 2002;21(3-4):298-302. Epub 2002/11/27. doi: S0264410X02004498 [pii]. PubMed PMID: 12450705.
68. Glass R, Ing D, Stoll B. Immune response to rotavirus vaccines among breast-fed and nonbreast-fed children. In: Jea M, editor. Immunology of Milk and the Neonate. New York: Plenum Press; 1991. p. 249–54.
69. Moon SS, Tate JE, Ray P, Dennehy PH, Archary D, Coutsoudis A, Bland R, Newell ML, Glass RI, Parashar U, Jiang B. Differential Profiles and Inhibitory Effect on Rotavirus Vaccines of Nonantibody Components in Breast Milk From Mothers in Developing and Developed Countries. Pediatr Infect Dis J. 2013;32(8):863-70. Epub 2013/04/16. doi: 10.1097/INF.0b013e318290646d. PubMed PMID: 23584581.
70. Blackwelder WC. Sample size and power for prospective analysis of relative risk. Stat Med. 1993;12(7):691-8. PubMed PMID: 8511445.
71. Fleiss JL, Tytun A, Ury HK. A simple approximation for calculating sample sizes for comparing independent proportions. Biometrics. 1980;36:343-6.
72. Zou GY, Donner A. Construction of confidence limits about effect measures: a general approach. Stat Med. 2008;27(10):1693-702. Epub 2007/10/26. doi: 10.1002/sim.3095. PubMed PMID: 17960596.
73. Conseil des Organisations Internationales des Sciences Médicales. Lignes directrices internationales d'éthique pour la recherche biomédicale impliquant des sujets humains

Geneva2003 [cited 2014 March 14]. Available from: http://www.cioms.ch/publications/guidelines/french_text.htm.
74. Bines J. Intussusception and rotavirus vaccines. Vaccine. 2006;24(18):3772-6. Epub 2005/08/16. doi: S0264-410X(05)00669-9 [pii]
10.1016/j.vaccine.2005.07.031. PubMed PMID: 16099078.
75. Greenberg HB. Rotavirus vaccination and intussusception--act two. N Engl J Med. 2011;364(24):2354-5. Epub 2011/06/17. doi: 10.1056/NEJMe1105302. PubMed PMID: 21675894.
76. Patel MM, Haber P, Baggs J, Zuber P, Bines JE, Parashar UD. Intussusception and rotavirus vaccination: a review of the available evidence. Expert Rev Vaccines. 2009;8(11):1555-64. Epub 2009/10/30. doi: 10.1586/erv.09.106. PubMed PMID: 19863248.
77. World Health Organization. Global Advisory Committee on Vaccine Safety, December 2011. Wkly Epidemiol Rec. 10 February 2012;6(87):53–60.
78. Bines JE, Ivanoff B, Justice F, Mulholland K. Clinical case definition for the diagnosis of acute intussusception. J Pediatr Gastroenterol Nutr. 2004;39(5):511-8. Epub 2004/12/02. doi: 00005176-200411000-00012 [pii]. PubMed PMID: 15572891.
79. Bines JE, Liem NT, Justice F, Son TN, Carlin JB, de Campo M, Jamsen K, Mulholland K, Barnett P, Barnes GL. Validation of clinical case definition of acute intussusception in infants in Viet Nam and Australia. Bull World Health Organ. 2006;84(7):569-75. Epub 2006/08/01. doi: S0042-96862006000700019 [pii]. PubMed PMID: 16878231; PubMed Central PMCID: PMC2627399.
80. World Health Organization. Post-marketing surveillance of rotavirus vaccine safety (WHO/IVB/09.01). Geneva: World Health Organization.
81. Shui IM, Baggs J, Patel M, Parashar UD, Rett M, Belongia EA, Hambidge SJ, Glanz JM, Klein NP, Weintraub E. Risk of intussusception following administration of a pentavalent rotavirus vaccine in US infants. JAMA. 2012;307(6):598-604. Epub 2012/02/10. doi: 10.1001/jama.2012.97
307/6/598 [pii]. PubMed PMID: 22318281.
