# Supplementary material for: Rotavirus vaccine efficacy up to 2 years of age and against diverse circulating rotavirus strains in Niger: Extended follow-up of a randomized controlled trial
Source: PLoS Med. 2021 Jul 2;18(7):e1003655. doi: 10.1371/journal.pmed.1003655 (PMC8253401; doi:10.1371/journal.pmed.1003655)
Supplement: S1 Statistical Analysis Plan — (PDF) [file pmed.1003655.s003.pdf]

Statistical Analysis Plan (SAP)

Protocol Title:

Randomized, double-blind, placebo-controlled phase III clinical trial to assess the efficacy and safety of a pentavalent rotavirus vaccine (BRV-PV) against severe rotavirus gastroenteritis among infants in Niger

Protocol: ROSE

Phase III

Effective Date: 13 July 2014

Protocol Version: 6.2

Statistical Analysis Plan (SAP)

Signature Page

**Type of document**	Statistical Analysis Plan	**Identifier**	ROSE
**Effective date**	13 July 2014	**Version number**	v1
		**Supersedes version**	None

Rebecca Grais<br>Research Directo	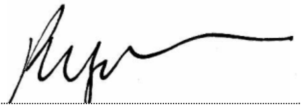	01 January 2015
Name and Title	Signature	Date
Name and Title	Signature	Date
Name and Title	Signature	Date
Name and Title	Signature	Date

Revision History

Version:	SOP ID:	Document Name:
Reason for change:		

SAP

Table of Contents

1. INTRODUCTION 8

1.1 STATISTICAL ANALYSIS PLAN 8

1.2 RATIONALE 8

2. STUDY DESIGN AND OBJECTIVES 9

2.1 STUDY OBJECTIVES 9

- *2.1.1 Primary Objective 9*
- *2.1.2 Secondary Objectives 9*

2.2 ASSESSMENT OF OBJECTIVES 10

- *2.2.1 Efficacy Assessments 10*
- *2.2.2 Safety Assessments 19*
- *2.2.3 Other Assessments 22*

2.3 CHANGE IN THE PLANNED ANALYSIS DURING THE CONDUCT OF THE STUDY **ERROR! BOOKMARK NOT DEFINED.**

2.4 STUDY DESIGN 23

2.5 SAMPLE SIZE AND POWER CONSIDERATIONS 23

2.6 RANDOMIZATION 24

2.7 BLINDING 24

3 GENERAL ANALYSIS REQUIREMENTS 25

3.1 STUDY DURATION 25

3.2 SCHEDULE OF STUDY VISITS, VISIT WINDOWS AND PROCEDURES 27

3.3 STUDY COHORTS 28

- *3.3.1 Inclusion Criteria 28*
- *3.3.2 Exclusion Criteria 28*
- *3.3.3 Withdrawal / Discontinuation Criteria 29*

3.4 TREATMENT ASSIGNMENT AND STUDY ARMS 29

4 STATISTICAL METHODS 30

4.1 ANALYSIS POPULATIONS 30

- *4.1.1 Intent-To-Treat (ITT) Analysis Population 30*
- *4.1.1.1 Per Protocol (PP) Analysis Population 30*
- *4.1.2 Immunogenicity Analysis Cohort 30*
- *4.1.3 Safety Population 30*
- *4.1.4 Protocol Deviations 31*

4.2 TIMING OF ANALYSIS 32

4.3 METHODS FOR HANDLING MISSING DATA 32
- *Handling of Screen Failures / Ineligible subjects 32*

4.4 STATISTICAL ANALYSIS 33
- *4.4.1 Derived Data 33*
- *4.4.2 Multiplicity Consideration 33*
- *4.4.3 Subject Disposition 33*
- *4.4.4 Baseline Characteristics 34*
- *4.4.5 Efficacy Analysis 36*
- *4.4.6 Safety Analysis 40*
- *4.4.7 Quality of Life Analysis 55*
- *4.4.8 Pharmacokinetics and Pharmacodynamic Analysis 55*

5 EVALUATION OF TREATMENT COMPLIANCE AND EXPOSURE 55

5.1 EXPOSURE TO STUDY DRUG 55
- *5.1.1 Extent of Exposure 55*

5.2 SUBGROUP / EXPLORATORY ANALYSIS 55
5.3 STATISTICAL ANALYTICAL ISSUES 55

6 INTERIM ANALYSIS AND SAFETY MONITORING ANALYSIS 55

6.1 INTERIM ANALYSIS 55
6.2 SAFETY MONITORING ANALYSIS 55

7 STATISTICAL TABLES TO BE GENERATED 56

8 STATISTICAL LISTINGS TO BE GENERATED 67

9 STATISTICAL GRAPHS TO BE GENERATED 68

List of Abbreviations

AE	Adverse Event
AR	Attack Rate
ARP	Attack Rate in Placebo Group
ARV	Attack Rate in Vaccinated Group
BCG	Bacillus Calmette-Guerin
BRV-PV	Bovine Rotavirus Pentavalent Vaccine
CI	Confidence Interval
CM	Concomitant Medication
CRO	Contract Research Organization
CRF	Case Report Form
DDC	Diarrhea Diary Card
DMC	Data Management Center
DSMB	Data and Safety Monitoring Board
ELISA	Enzyme-Linked Immunosorbent Assay
EW	Early Withdrawal
FFU	Fluorescent Focus Units
GCP	Good Clinical Practice
GE	Gastroenteritis
GMT	Geometric Mean Titre
ICF	Informed Consent Form
ICH	International Conference on Harmonization
IEC	Independent Ethics Committee
IMNCI	Integrated Management of Neonatal and Childhood Illnesses
ITT	Intent to Treat
IP	Investigational Product
IVRS	Interactive Voice Response System
IWRS	Interactive Web Response System
IgA	Immunoglobulin A
Ho	Null Hypothesis
H_1	Alternative Hypothesis
LLN	Lower Limit of Normal
Max	Maximum
MedDRA	Medical Dictionary for Drug Regulatory Activities
MEM	Minimal Essential Medium

mg	milligram
Min	Minimum
mL	milliliter
N	Number of Subjects
OC	Observed Case
PI	Principal Investigator
PIDC	Post-Immunization Diary Card
PPT	Per Protocol
PSRT	Protocol Safety Review Team
RR	Relative Risk
RV	Rotavirus
RVGE	Rotavirus Gastroenteritis
SAE	Serious Adverse Event
SD	Standard Deviation
SII	Serum Institute of India, Limited
SOC	System Organ Class
SOP	Standard Operating Procedure(s)
SAGE	Strategic Advisory Group of Experts on Immunization
SRVGE	Severe Rotavirus Gastroenteritis
ULN	Upper Limit of Normal
VE	Vaccine Efficacy
WHO	World Health Organization
WHO- DD	World Health Organization Drug Dictionary

1. Introduction

1.1 Statistical Analysis Plan

A statistical analysis plan is a comprehensive and detailed description of the methods for data analyses to be used in a clinical trial. A clear detailed SAP will avoid post hoc decisions that may affect the interpretation of the data. This SAP includes details on the procedures for creating Tables, Listings, and Graphs (TLGs) from the results of a phase 3 study, which was carried out to evaluate safety, immunogenicity, and the efficacy of a 3-dose series of BRV-PV administered orally to prevent severe rotavirus gastroenteritis in healthy infants.

This statistical analysis plan will provide full details of the analysis to be presented in the Tables, Listings, and Graphs. This is a separate document apart from statistical section of the protocol. Any changes in the statistical analysis / methods planned in the protocol and even if any additional statistical analysis is planned which is not part of the protocol will be explained in this detailed statistical analysis plan of the study. The SAP was developed before any data was unblinded to the study team.

This SAP:

- Includes a statement of the objectives of the trial, as stated in the protocol.
- Identifies all primary and secondary end-points.
- Specifies the hypotheses to be tested and any parameters that are to be estimated, in order to meet the trial objectives.
- Defines the analysis populations to be used
- Provides a full and detailed description of the methods of analysis including details of handling of missing data, dropouts, derived variables, etc.

By the time this SAP was competed and approval by all parties received, the trial data was still under blind.

1.2 Rationale

Sub-Saharan Africa carries the largest burden of rotavirus-related mortality, but immunization against rotavirus presents unique challenges. Current supply of the 2 WHO prequalified vaccines is constrained, and in many African settings, national immunization programs are challenged by lack of trained health workers. Unreliable transportation systems and storage facilities also make it difficult to preserve vaccines that require refrigeration. If rotavirus vaccine is to be brought to the infants that need it most through national immunization programs in the region, new vaccines that address these challenges are urgently needed. The BRV-PV vaccine is a relatively low-cost and heat-stable formulation whose introduction into

national immunization programs may help minimize the burden on already-strained national programs throughout sub-Saharan Africa.

The WHO Expert Committee on Biological Standardization has recommended that the efficacy of new rotavirus vaccines be demonstrated in diverse geographical regions including developing countries before widespread implementation. The Ministry of Health of Niger, Médecins Sans Frontières (MSF) – Operational Center Geneva and Epicentre along with other partners have formed a research consortium to bring additional evidence to inform public health decision making on the potential value of the BRV-PV vaccine in an African setting. The goal of the present study is to collect additional data on the efficacy profile of BRV-PV vaccine in a randomized controlled setting, while gaining further experience with vaccine-related adverse events. This will be conducted through the performance of a phase III trial in Niger conducted in compliance with the version of the protocol agreed to by the applicable regulatory authorities and Good Clinical Practice (GCP).

Evidence supporting the efficacy and safety of this formulation in an African setting would support the pre-qualification and increased global access to the BRV-PV vaccine. If shown to be efficacious and pre-qualified, the government of Niger would benefit from a low cost vaccine adapted to the logistical and supply demands of the national immunization program.

2. Study Design and Objectives

2.1 Study Objectives

2.1.1 Primary Objective

The Primary objective of the study is:

- To estimate the efficacy of three doses of SIIL BRV-PV vaccine vs. placebo against severe rotavirus gastroenteritis from 28 days post-Dose 3 up to 2 years of age in healthy infants in Niger.

2.1.2 Secondary Objectives

Secondary objectives are:

- To estimate the efficacy of the SIIL BRV-PV vaccine vs. placebo against severe rotavirus gastroenteritis from 28 days post-Dose 3 to 1 year of age and from 1 to 2 years of age.
- To evaluate vaccine efficacy against rotavirus gastroenteritis of any severity.
- To estimate vaccine efficacy against rotavirus gastroenteritis with a Vesikari score ≥ 17.
- To estimate vaccine efficacy against gastroenteritis and severe gastroenteritis of any cause.

- To estimate vaccine efficacy against severe rotavirus gastroenteritis caused by G serotypes included in the vaccine (G1, G2, G3, G4 and G9).
- To estimate longitudinal prevalence of rotavirus gastroenteritis.
- To estimate vaccine efficacy to reduce hospitalizations due to rotavirus gastroenteritis and rotavirus gastroenteritis.
- To estimate vaccine efficacy to reduce hospitalization for any reason.
- To estimate safety of the SIIL BRV-PV vaccine vs. placebo (adverse events [AEs] and serious adverse events [SAEs]).
- To estimate the immunogenicity of BRV PV in a sub-sample of participants.
- To estimate effect of prenatal nutritional supplementation on infant immune response to the vaccine in a sub-sample of participants.
- To demonstrate the immunological non-inferiority of EPI vaccines when co-administered with the BRV-PV as compared to their co-administration with placebo.

2.2 Assessment of Objectives

2.2.1 Efficacy Assessments

To identify and study all cases of gastroenteritis, cases of gastroenteritis episodes will be captured through facility- and home-based surveillance from the moment the first dose of vaccine or placebo is administered until 2 years of age. All Gastroenteritis episodes will be captured in the Gastroenteritis Surveillance CRF. Given the time interval between the first and 28 days post the third dose, a period over which many subjects may develop rotavirus gastroenteritis, without having received the benefit of full vaccination, gastroenteritis episodes between the first and 28 days post the third dose will not be counted in the primary analysis. All efficacy analyses will use all follow-up through 117 cases (primary analysis) and up to 2 years of age (end of follow up), as well as up to 1 year of age and 1-2 years.

Thus efficacy endpoints assessments will be performed based on following applicable definitions mentioned in protocol:

Gastroenteritis (GE): GE is defined as the passing of three or more watery or looser-than-normal stools within 24-hour period, with or without forceful vomiting. Every episode of GE will be evaluated clinically and the combined symptoms and signs summarized using the Vesikari Severity Score system. An episode with a Vesikari score of ≥11 will be considered as a severe GE case (see Section 4.4.6.3 for details on Vesikari score). Gastroenteritis episodes will be classified as two separate episodes if there is an interval of 5 or more consecutive, symptom-free days between the episodes.

Rotavirus Gastroenteritis (RVGE): A case of RVGE will be defined as the production of three or more watery or looser-than-normal stools within 24-hour period, with or without forceful vomiting, along with

the detection of rotavirus in a stool specimens obtained within seven days after the end of symptoms. The RVGE cases will be ascertained by Laboratory and include all serotypes unless specified otherwise.

Severe Rotavirus Gastroenteritis (SRVGE): If Laboratory confirmed RVGE case is qualified as severe as per Vesikari score. If Vesikari score ≥ 11 as per data captured on the Gastroenteritis Surveillance CRF then RVGE is considered as SRVGE case.

Vaccine Efficacy is expressed as proportionate reduction in disease attack rate (AR) between placebo (ARP) group i.e. placebo group and vaccinated (ARV) groups i.e. rotavirus vaccine group will be calculated from hazard ratio (HR) of disease among the rotavirus vaccine (vaccinated) group using the following formula:

Vaccine Efficacy = (1- HR)*100, HR = ARV / ARP and is based on each subject follow-up time in years / Time to Event.

There are potentially two analysis time points for this study. The first analysis time point when 117 per protocol subjects with at least one SRVGE occurring from 28 days after the third vaccine/placebo dose. This will be the primary analysis time point for the primary efficacy endpoint for the study and will be referred to as the primary analysis period with the first 117 SRVGE cases. The secondary analysis time point is when all participating subjects reach two years of age and will be referred to as the entire study period up to 2 years of age. If the accrual of 117 per protocol subjects doesn’t happen prior to all subjects reaching two years of age, there will be only one analysis time point.

2.2.1.1 Primary Efficacy Endpoints

Section	Planned Endpoint	Analysis Time Point Planned in the Protocol	Assessment Details about Primary Endpoint
2.2.1.1.1	SRVGE cases	Primary Analysis Time Point:<br>117 cases of SRVGE accrued or until all participating subjects reach two years of age	• Will be assessed in subjects who received complete correct vaccination regimen of three doses of vaccine or placebo included in the PP population - specified vaccination windows) as defined in section 4.1.6. Subjects that receive incorrect product, or receiving product out of the acceptable vaccination windows will not be included.<br>• First episode of SRVGE occurring from 28 days after the 3rd dose of the study vaccine/placebo in a subject will count towards the primary endpoint for PP populations defined above. Second/third episodes of SRVGE will not count for the primary analysis.<br>• This will be repeated for ITT as Secondary analysis which will include all the randomized infants who received at least one vaccine dose, independent of the vaccination windows and whether or not they received the correct IP.

2.2.1.2 Secondary Efficacy Endpoints

Section	Planned End Points as per Protocol	Analysis Time Point Planned in the Protocol	Assessment Details about Secondary Endpoint
2.2.1.2.1	SRVGE cases matched with Serotypes included in vaccine	Primary Analysis Time Point :<br>117 cases of SRVGE accrued or until all participating subjects reach two years of age	• By individual serotype, vaccine-specific serotype, G1, 2, 3, 4 vs. others<br>• Will be assessed in PP population and in ITT population.<br>• All SRVGE episodes available in the analysis populations will be taken into consideration and first case of each G serotype will be counted for each subject to find efficacy with respect to each G serotype strain.
2.2.1.2.2	SRVGE cases matched with Serotypes included in vaccine	Up to 2-years of age (End of Study)	• Will be assessed in PP population and in ITT population.<br>• All SRVGE episodes available in the analysis populations will be taken into consideration and first case of each G serotype will be counted for each subject to find efficacy with respect to each G serotype strain.
2.2.1.2.3	RVGE cases	Primary Analysis Time Point :<br>117 cases of SRVGE accrued or until all participating subjects reach two years of age	• Will be assessed in PP population and in ITT population.<br>• First episode of RVGE available in the analysis populations will count towards this secondary endpoint.

Section	Planned End Points as per Protocol	Analysis Time Point Planned in the Protocol	Assessment Details about Secondary Endpoint
2.2.1.2.4	SRVGE cases	Up to 1-year of age	• Will be assessed in PP population and in ITT population.<br>• First episode of SRVGE available in the analysis populations will count towards this secondary endpoint.
2.2.1.2.5	SRVGE cases	From 1-year of age up to 2-years of age	• Will be assessed in PP population and in ITT population.<br>• First episode of SRVGE available in the analysis populations will count towards this secondary endpoint.
2.2.1.2.6	SRVGE cases	Up to 2-years of age (End of Study)	• Will be assessed in PP population and in ITT population.<br>• First episode of SRVGE available in the analysis populations will count towards this secondary endpoint.<br>.
	RVGE cases with Vesikari ≥ 17		
	RVGE cases by 1 and 2 doses		

Section	Planned End Points as per Protocol	Analysis Time Point Planned in the Protocol	Assessment Details about Secondary Endpoint
	RVGE cases by GMT		

Section	Planned End Points as per Protocol	Analysis Time Point Planned in the Protocol	Assessment Details about Secondary Endpoint
2.2.1.2.9	GE cases / 100 children-year	Up to 2-years of age (End of Study)	• Will be assessed in PP population and in ITT population.<br>• First episode of SRVGE available in the analysis population will count towards this secondary endpoint.<br>• To be done for SRGE, RVGE and GE.<br>• To be estimated as number of diarrhea days / child years.<br>• Vaccine Impact for 2-year follow-up of subject age.
2.2.1.2.10	All Severe GE episodes (may or may not be RVGE episode)	Primary Analysis Time Point : 117 cases of SRVGE accrued or until all participating subjects reach two years of age	• Will be assessed in PP population and in ITT population.<br>• All episode of severe GE available in the analysis population will count towards this secondary endpoint

Section	Planned End Points as per Protocol	Analysis Time Point Planned in the Protocol	Assessment Details about Secondary Endpoint
2.2.1.2.11	All Severe GE episodes (may or may not be RVGE episode)	Primary Analysis Time Point and Up to 2-years of age (End of Study)	• Will be assessed in PP population and in ITT population.<br>• All episode of severe GE, irrespective of rotavirus status, occurring post study vaccine/placebo administration will count towards this secondary endpoint<br>The occurrence of all severe GE episodes will be considered as defined in ITT population.
2.2.1.2.12	All GE episodes (may or may not be RVGE episode)	Primary Analysis Time Point : 117 cases of SRVGE accrued or until all participating subjects reach two years of age	• Will be assessed in PP population and in ITT population.<br>• All episode of GE irrespective of severity and rotavirus status, occurring available in the analysis population will count towards this secondary endpoint<br>The occurrence of all severe GE episodes will be considered as defined in ITT population.
2.2.1.2.13	All GE episodes (may or may not be RVGE episode)	Up to 2-years of age (End of Study)	• Will be assessed in PP population and in ITT population.<br>• All episodes of GE irrespective of severity available in the analysis population will count towards this secondary endpoint<br>The occurrence of all severe GE episodes will be considered as defined in ITT population.

Section	Planned End Points as per Protocol	Analysis Time Point Planned in the Protocol	Assessment Details about Secondary Endpoint

2.2.1.3 Immunogenicity Endpoints

A subset of up to 1320 subjects will be enrolled for the immunogenicity endpoints.

To determine and quantitative vaccine seroresponses, 3 mL of blood will be collected before administration of 1st dose and 28 days (±7 days) after 3rd dose administration. The sera will be tested in a validated anti-rotavirus IgA ELISA assay at Cincinnati Children's Hospital? The rate of seroresponse, i.e. Percentage (%) of subjects with ≥3-fold rise and ≥4-fold rise from baseline to 28 days post Dose 3, and Geometric Mean Titer (GMT) will be compared among vaccine and placebo recipients.

Section	Planned Endpoints as per protocol	Analysis Time Point Planned in the Protocol	Assessment Details about Secondary Endpoint
2.2.1.3.1	Anti-rotavirus IgA ELISA assay: Seroresponse Rates in a subset of up to 1320 Infants: Percentage (%) of subjects with ≥3-fold increase from baseline at 28 days after 3rd dose in	To be performed at Primary Analysis Time Point: 117 cases of SRVGE accrued OR at Final	For subjects in immunogenicity cohorts sera will be obtained immediately before the first dose of vaccine (Baseline) and 28 ± 7

Section	Planned Endpoints as per protocol	Analysis Time Point Planned in the Protocol	Assessment Details about Secondary Endpoint
2.2.1.3.2	Anti-rotavirus IgA ELISA assay: Seroresponse Rates in a subset of 200 Infants: Percentage (%) of subjects with ≥ 4-fold increase from baseline at 28 days after 3rd dose in rotavirus IgA titers	analysis i.e. at the end of 2-years age follow-up period – Based on completed testing of all samples collected from the immunogenicity cohort	Days post 3rd dose of vaccination, to test for rotavirus antibody in an ELISA IgG assay.
2.2.1.3.3	Geometric mean Titers (GMTs) in all subjects regardless of baseline value and by baseline sero-status (i.e. seropositive defined as value ≥20 IU/ml and seronegative defined as value <20 IU/ml)		
2.2.1.3.4	Percentage (%) of subjects with rotavirus IgA titers ≥ 20 IU/ml at 28 days after 3rd dose (among those with baseline status < 20 IU / ml)		

2.2.2 Safety Assessments

The safety will be monitored by recording AEs and SAEs experienced by subjects.

All AEs occurring through 28 days after 3rd vaccination and all SAEs will be reported and captured in CRF during study period.

2.2.2.1 Secondary Safety Endpoints

2.2.2.1.1 Immediate Post Vaccination Reactions (within 30 min Post Vaccination)

After each dosing, all participants will be kept at the clinic site for 30 minutes to check for any immediate AEs. At 30 minutes post-vaccination, vital signs will be measured and a targeted physical examination will be performed.

2.2.2.1.2 All Adverse Events (AEs)

An AE is any untoward medical occurrence in a clinical investigation subject administered a pharmaceutical product and that does not necessarily have a causal relationship with this treatment. An AE can therefore be any unfavorable and unintended sign (including an abnormal laboratory finding), symptom, or disease temporally associated with the use of a medicinal (investigational) product, whether or not related to the medicinal (investigational) product. AEs include all events, including but not limited to fever, diarrhea, vomiting, decreased appetite, decreased activity level, otitis media, and nasopharyngitis. All adverse events will be assessed using facility- and home-based surveillance in all participants and graded for severity from the time of first dose until 28 days post-Dose 3. Caregivers will be informed about the signs and symptoms of adverse events and will be asked to seek care at a local facility or inform a study member in the village in the event any adverse event is suspected and of concern. Study staff will record history of symptoms of the current illness through caregiver interview and conduct a physical examination to document physical signs and clinical condition.

The analysis of adverse events will be done using the ITT population, i.e. all infants who received at least one dose of the study vaccine or placebo, and include follow up from the time of enrollment until 28 days post-dose 3. The incidence of adverse events will be compared between groups with the two-sided asymptotic score test for the null hypothesis of identical incidence by group.

2.2.2.1.3 All Serious Adverse Events (SAEs)

All SAEs and follow-up information occurring between the time of randomization through second year of age will be analyzed. Serious adverse events are defined as follows:

- Results in death
- Is life threatening
- Requires inpatient hospitalization* or prolongation of existing hospitalization
- Results in persistent or significant disability**/incapacity, or
- is medically important event / reaction that may jeopardize the subject and may require medical or surgical intervention to prevent one of the outcomes listed above

The term "life-threatening" in the definition of "serious" refers to an event in which the patient was at risk of death at the time of the event; it does not refer to an event which hypothetically might have caused death if it were more severe.

Hospitalization is an official admission to a hospital with overnight stay. Hospitalization or prolongation of a hospitalization constitutes a criterion for an AE to be serious**Disability is defined as a substantial disruption in a person’s ability to conduct normal life functions. If there is any doubt about whether the information constitutes an SAE, the information is treated as an SAE. All SAEs and follow-up information occurring between the time of randomization and second year of age will be reported in expedited fashion independent of the relationship to study product.

SAEs will be coded using the MedDRA dictionary, Version 16.0. The exact version of the dictionary will be mentioned in the footnote of the respective Listing and/or Table.

Intussusception will qualify as an SAE for this study. Any SAE for which if MedDRA Preferred Term is coded as “Intussusception” will be considered to analyze this endpoint.

SAEs for all Infants will be analyzed at Primary Analysis Time Point i.e. when 117 cases of SRVGE will be accrued and at the end of the study.

2.2.2.1.4 Death

Any SAE for which “Outcome” is marked as “Fatal” will be considered to analyze this endpoint.

Deaths will be analyzed at Primary Analysis Time Point i.e. when 117 cases of SRVGE will be accrued and at the End of the study.

2.2.2.1.5 Hospitalization

Any SAE that is accompanied by hospitalization will be considered to analyze this endpoint. Hospitalizations will be analyzed at Primary Analysis Time Point i.e. when 117 cases of SRVGE will be accrued and at the End of the study.

2.2.2.1.6 Intussusception

Any SAE for which AE Preferred term as per MedDRA dictionary is reported as “Intussusception” will be considered, to analyze this endpoint.

Medical personnel at the sites will be trained on screening tools to promptly identify and assess any suspected cases of intussusception. They include bloody stools, continuous vomiting, abdominal distension and/or abdominal “lumps”. Children presenting any suspected symptoms at the time of home

visits or presentation to a study facility will be referred for evaluation. Parents will be instructed by staff to keep close watch of the symptoms noted above and contact study staff if they are detected.

An Intussusception Adjudication Committee constituted by experts (pediatricians, pediatric surgeons) will be formed to review all suspected cases of intussusception and make a final determination on the diagnosis in each case.

The Intussusception cases that are confirmed by Intussusception Adjudication Committee will be reported as SAE with Preferred term as “Intussusception” in the CRF.

Intussusception will be analyzed at Primary Analysis Time Point i.e. when 117 cases of SRVGE will be accrued and at the end of the study.

2.2.3 Other Assessments

2.2.3.1 Vital Signs

Vital signs will be performed at every clinic visit from screening Vital Signs will be assessed twice at visits where study product is administered, with the first assessment before administration of Vaccine and the second assessment done 30 minutes post Vaccine administration. Vital Signs measurement will consist of following parameters:

- Axillary Temperature (^{0}Celsius)
- Heart Rate (Beats/min)
- Respiratory Rate (Breaths/min)

2.2.3.2 Physical Examination

Physical Examination will be will be assessed twice at every vaccination visits, first assessment will be done before administration of Vaccine and the second assessment will be done 30 minutes post Vaccine administration.

.

General Physical Examination of the following parameters and body systems:

- Head and Neck
- Eye
- Ears, Nose and Throat
- Skin
- Musculoskeletal
- Central Nervous System

- Respiratory
- Cardiovascular
- Gastrointestinal
- Genitourinary

2.2.3.3 Concomitant Medications

Concomitant medications will be recorded throughout the study period. Concomitant medications will be coded using WHO- DD Version March 2013.

2.3 Study Design

The study is designed as a multisite, double-blinded, randomised, end-point driven placebo controlled trial with two parallel groups of subjects receiving either vaccine or placebo at a 1:1 allocation to evaluate efficacy, safety and immunogenicity of BRV-PV given to healthy subjects aged 6-8 weeks (42-56 days) at the time of first dose of vaccination. Participants will be enrolled in multiple sites across Maradi, Niger. Three doses of BRV-PV/Placebo to healthy subjects. BRV-PV will contain ≥ Log105.6 FFU/dose of each rotavirus serotype G1, G2, G3, G4 and G9.

A total of 7,770 participants will be enrolled in the study. Allocation of treatment to individual subjects will be based on a randomization schedule. The participants will receive three oral administrations as follows:

BRV-PV<br>n = 3,885	Will receive lyophilized BRV-PV reconstituted with 2.5 ml of buffered diluent
Placebo<br>n = 3,885	Will receive lyophilized placebo, reconstituted with 2.5 ml of buffered diluent

Infants will be screened at 6-8 weeks (42-56 days) of age if parent consent is available. Of those screened, eligible subjects will be enrolled and given the first administration of the test vaccine/placebo at 6-8 weeks (42-56 days) of age followed by two more doses at one month (and up to four additional weeks) intervals.

2.4 Sample Size and Power Considerations

The primary aim of the study is to assess the efficacy of the study vaccine against severe rotavirus gastroenteritis. The point estimate of the vaccine efficacy (VE), i.e., [1 – Hazzard Ratio of the vaccine group relative to the placebo group] x 100%], and the associated 95% CI will be calculated using the Cox proportional hazards model.

Assuming a 2% attack rate of severe rotavirus gastroenteritis, a 50% true vaccine efficacy and a 20% participant non-assessibility (including withdrawal and loss to follow up), the study will enroll 3885 children per group (total n = 7770) to have at least 90% power to detect a vaccine efficacy with a lower 95% confidence interval bound greater than 0%. Under these assumptions, a sample size of 7,770 participants will result in 117 cases of severe rotavirus gastroenteritis (78 unvaccinated and 39 vaccinated) required to fulfill the primary study objective and trigger the primary analysis.

The trial is event driven, with the final analysis triggered by the occurrence of the 117nd case of primary endpoint (78 unvaccinated and 39 vaccinated)). If not all of the 117 needed cases are accrued by the time all subjects reach their second year of age, the analysis of efficacy will be conducted based on all available data.

2.5 Randomization

Eligible participant will be randomised to one of the two groups (1:1) to receive either SII BRVPV or Placebo. Randomization is defined as the process of assigning a participant to a study arm. Randomization will be carried out by assigning each participant a code used for blind allocation to vaccine or placebo.

2.6 Blinding

The study will be conducted in double-blind manner. The clinical site staff and Sponsor will be blinded to treatment assignments. Only DSMB, designated statistician, IP packaging, labeling, Storage & distribution personnel and CRO/third party vendor packaging personnel who are not involved in the trial, will be unblinded to treatment assignments.

The code for an individual participant should be broken only in case of medical emergency where the identity of the IP must be known in order to properly treat the study subject. The investigator must contact the sponsor for concurrence. If agreed, the IP assignment information will be provided through the CRO. All such cases will be fully documented by the investigator and written notification should be provided to sponsor

Since this is endpoint driven study the primary analysis will be performed once 117 SRVGE cases are accrued and if not then primary analysis will be done at the end of the study. If 117 SRVGE cases accrued prior to all subjects reach 2 years of age then the unblinded analysis will be performed. All study site personnel involved in the study conduct will remain blinded at subject level until the completion of the study. Selected members of sponsor, SIIL, will be unblinded at group or subject level to prepare for the clinical study report and the regulatory submission dossiers.

3 General Analysis Requirements

3.1 Study Duration

Projected duration of accrual is about 18 months. Active surveillance for SRVGE will take place by weekly contacts with the participating subjects starting from the time of the first vaccination until subjects reach two years of age. The primary endpoint will be achieved when 117 cases of severe rotavirus gastroenteritis (SRVGE) are accrued, or until all participating subjects reach two years of age.

The conduct of the study trial from Screening Visit to End of Study Visit is as follows:

- Screening and Dose 1:
    - Written Informed Consent, Subject Demography, Vital Signs, Physical examination, evaluation of Inclusion/Exclusion Criteria, Medical History
    - Randomization
    - Study Vaccination
    - EPI Vaccination
    - Blood collection for immunogenicity in sub cohort
    - Safety assessments (Vital Signs, Physical examination, AEs, SAEs, and Concomitant Medications)
- Dose 2:
    - Study Vaccination
    - EPI Vaccination
    - Safety assessments (Vital Signs, Physical examination, AEs, SAEs, and Concomitant Medications)
- Dose 3:
    - Study Vaccination
    - EPI Vaccination
    - Safety assessments (Vital Signs, Physical examination, AEs, SAEs, and Concomitant Medications)
- Dose 3: Day 28day post Dose 3
    - Blood collection for immunogenicity
    - Safety assessments (Vital Signs, Physical examination, AEs, SAEs, and Concomitant Medications)
- 6month (visit 5) ,9 month (Visit 6), 12 month (Visit 7), 18 Month (Visit 9) and 24 month (Visit 10)
    - Medical History

- Safety assessments (Vital Signs, SAEs, and Concomitant Medications)
- Blood collection for immunogenicity (12 and 24 months)

3.2 Schedule of Study Visits, Visit windows and Procedures

Table 2: Time and Events Table

WEEK OF AGE		W0	W6	W10	W14	W18	W24	W36	W52	W64	W76	W88	W104
**Study Visit**	Pregnancy	Pre - random-ization	Random-ization and Dose 1	Dose 2	Dose 3	Dose 3 + 28 days	6 months	9 months	12 months	15 months	18 months	21 months	24 months
**ALL PARTICIPANTS**													
Home Visit*			→										
Scheduled Facility Visit			X	X	X	X	X	X	X		X		X
Surveillance													
Gastroenteritis and SAE			X	X	X	X	X	X	X	X	X	X	X
AE**			X	X	X	X							
Laboratory Assessment													
Child stool***			X	X	X	X	X	X	X	X	X	X	X
**IMMUNOGENCITY SUB-COHORT**													
Home Visit*	→												
Laboratory Assessment													
Child stool***			X	X	X	X	X	X	X	X	X	X	X
Child blood			X			X			X				X
Child urine			X	X	X	X	X	X	X	X	X	X	X
Maternal blood	X	X	X				X						
Maternal stool	X	X	X				X						
Maternal urine	X	X											
Breast milk		X	X				X						

* Home Visits scheduled for Pre-randomization and on a weekly basis until 2 years of age. In the immunogenicity sub-cohort, home visits will be conducted among all consenting women of reproductive age and continue until the child is 2 years of age.

** Surveillance for adverse events from the time of Dose 1 until 28 days post-Dose 3.

*** Stool collected from all participants for any case of gastroenteritis identified at facility or home within a recall period of 7 days until 2 years of age. In the immunogenicity sub-cohort, stool samples will be collected independent of gastroenteritis status at the time of each Dose, 28 days post-Dose 3, 6 months of age and every 3 months thereafter until 2 years of age.

3.3 Study Cohorts

Participants will be 6-8 weeks old healthy subjects. Potential volunteer families may be contacted from before the babies are born through the noted targeted age. Final eligibility determination will depend on the results of the medical history, clinical examination, fulfillment of the inclusion and absence of any of the exclusion criteria, appropriate understanding of the study and completion of the consent process. All subjects targeted for enrollment will need to have parents that can comprehend the purpose of the study and provide written informed consent. In addition, the families should be resident in the area without plans to leave the study site during the course of the study. Sufficient number of healthy subjects will be screened with parental consent to enroll seven thousand seven hundred seventy participants in the study.

3.3.1 Inclusion Criteria

Fulfillment of all of the following criteria is required to accept the subject in the study:

(1) aged 6-8 weeks at the time of inclusion

(2) able to swallow and no history of vomiting within 24 hours

(3) resident in Madarounfa Health District and within the catchment area of the health facilities

(4) intending to remain in the study area for 2 years

(5) parent/guardian providing written informed consent

3.3.2 Exclusion Criteria

Any of the following will exclude the subject from the study:

(1) known history of congenital abdominal disorders, intussusception, or abdominal surgery

(2) receipt of intramuscular, oral, or intravenous corticosteroid treatment within 2 weeks

(3) receipt or planned administration of a blood transfusion or blood products, including immunoglobulins

(4) any known immunodeficiency condition

(5) any serious medical condition

(6) any other condition in which, in the judgment of the Site Principal Investigator, would interfere with or serves as a contraindication to protocol adherence or the parent/guardian’s ability to give informed consent.

Presence of vomiting in the previous 24 hours or on the day of enrollment, immediate hospitalization, and inability to swallow IP are temporary exclusions.

After informed consent has been obtained and the child is identified as meeting inclusion and exclusion criteria for enrollment, the child will be enrolled in the study and assigned a randomization number.

3.3.3 Withdrawal / Discontinuation Criteria

The participants may be withdrawn from the study for any of the following situations:

- If parent of subject wishes to withdraw consent
- If families move away from the study site permanently. However the data collected up to the last contact will be part of the analysis.
- If it is felt in the Principal Investigator's (PI's) opinion that further participation in the study may be detrimental to the interests of the participant
- Subject lost to follow-up

In all such cases, the participant will be withdrawn from the study and the reason for withdrawal will be documented in an appropriate case report form. Subjects who move away from the study site or are lost to follow-up and later present to the site and are willing to continue participation, will continue to be followed through age two.

All subjects, who withdraw early from the study for any reason, will be encouraged to complete the end of study assessments as well as their scheduled EPI vaccines. Study subjects whose participation in the study is terminated by the investigator will remain eligible for care at the site until two years of age.

Vaccinations may be discontinued, but follow-up continued, for any of the following situations:

- If subject suffers from immediate hypersensitivity reaction following vaccination
- If subject suffers from significant inter-current illness If there is protocol violation
- If the participant receives a licensed rotavirus vaccine
- If it is felt in the Principal Investigator's (PI's) opinion that it is not in the subject's best interest to continue vaccinations

3.4 Treatment Assignment and Study arms

Based on a central computer-generated randomization schedule, subjects will be randomised to one of the two study arms:

- BRV-PV
    - Live Attenuated Pentavalent (G1-G2-G3-G4-G9) Human X Bovine Reassortant Rotavirus Vaccine (BRV-PV), at a dosage of ≥ Log105.6 fluorescent focus units (FFU)/Serotype/Dose in 2.5 ml of buffered diluent
- Placebo

- Lyophilized minimal essential medium (MEM) + excipients reconstituted in 2.5 ml of buffered diluents

4 Statistical Methods

4.1 Analysis Populations

4.1.1 Intent-To-Treat (ITT) Analysis Population

The Intent-To-Treat (ITT) analysis population will consist of all randomised subjects who have taken at least one dose of study vaccine/placebo, independently of whether they receive the full appropriate regimen of vaccine/placebo or receiving the incorrect IP.

In ITT population subjects will be analyzed as per the randomised vaccine group and not as per the actual vaccine received at Dose 1.

4.1.2 Per Protocol (PP) Analysis Population

Per-Protocol analysis population is the subset of the ITT subjects with no major (important) protocol deviations which will impact primary / secondary efficacy analysis and subjects who have received all three doses of study vaccine/placebo and all three doses received are as per the randomised vaccine group.

The PP analysis population will be the primary cohort for the primary and secondary efficacy analyses.

4.1.3 Immunogenicity Analysis Cohort

Immunogenicity analysis population is a subset of approximately 1320 participants in the PP population for whom permission is obtained to assess immune response to the vaccine. To meet the desired target of 1320 evaluable paired pre- and post-vaccination blood samples, sufficient number of subjects will be enrolled in this subset. The CRO will assure equal numbers of vaccine and placebo recipients are enrolled in the immunogenicity cohort. This analysis population includes the subjects with valid measurement(s) required for the corresponding endpoint.

4.1.4 Safety Population

Safety population will consist of all randomised subjects who have received at least one dose of study vaccine/placebo, with or without EPI vaccines, and have some safety data available.

This population will be used for AEs, SAEs, hospitalizations, deaths, and Intussusceptions through Two Years of Age. Once a subject is determined to be in the safety population, the subject will be included in all safety analyses regardless of the availability of individual endpoint data.

In this analysis population subjects will be analysed as per the actual vaccine received at Dose 1. If subject receives mixed dosing then the safety data for dose wise analysis will be analysed on the basis of actual vaccine received for particular dose and for overall (all doses combined) the subject will be analysed as

per the actual vaccine received at Dose 1. Listing of mixed dosed subjects will be provided and any significant findings (e.g. IAE, SAE, Death or Intussusception and Related AE) then that will be discussed individually in the clinical study report.

4.1.5 Protocol Deviations

Protocol deviations will be tracked by the study team throughout the conduct of the study.

    - Blind review of Protocol Deviation Listing will be performed by Sponsor personnel and statistician prior to freezing the database and will be finalized and approved to ensure all important deviations, and deviations which may lead to exclusion from the analysis, are captured and categorized on the protocol deviations dataset.
    - Protocol Deviation dataset (Final Protocol Deviation Listing) will be the basis for the summaries and listings of protocol deviations.

- All Important protocol deviations (including deviations related to study inclusion/exclusion criteria, conduct of the trial, patient management or patient assessment) will be summarised and listed.
- Important deviations which result in exclusion from the analysis population will also be summarised and listed. (Please refer Table 3 below in the section "Protocol Deviation Management and Definitions for Per Protocol and Immunogenicity population".
- Based on vaccine/placebo administration window period the PP population is as follows:
    - **PP (based on the protocol defined vaccination window) Population:** This population follows window period defined in the protocol for each dose administration of vaccine / placebo as follows:

      Dose 1: 6-8 weeks of age i.e. 42-56 Days (both inclusive) of age at the time of first dose vaccination

      Dose 2: 4 weeks from Dose 1 (with a window of –1 to +4 weeks) implies 28 days from Dose 1 (with a window of 21 days to 56 days from Dose 1) implies 21 ≤ Dose 2 date – Dose 1 Date ≤ 56 days at the time of second dose vaccination.

      Dose 3: 4 weeks from Dose 2 (with a window of –1 to +4 weeks) implies 28 days from Dose 2 (with a window of 21 days to 56 days from Dose 2) implies 21 ≤ Dose 3 date – Dose 2 Date ≤ 56 days at the time of Third dose vaccination.

      Vaccine Efficacy evaluated using this population will be considered as primary analysis.

4.1.5.1.1 Protocol Deviation Management and Definitions for Per Protocol and Immunogenicity Population

Protocol deviations which impact upon the Per Protocol (PP) and Immunogenicity analyses can be full, partial, or time point-specific. Subjects will be excluded from the Per Protocol analysis Population if they have a protocol deviation defined as a Full exclusion in the relevant column in Table 3. Similarly, subjects will be excluded from the Immunogenicity cohort if they have a protocol deviation defined as a Full exclusion in the relevant column in Table 3.

Subjects identified as partial protocol deviation will be included in the PP population but will have their data excluded from PP analyses from the time of deviation onwards. Subjects with time-point specific protocol deviations will be included in the PP Population but only those data affected by the deviation will be excluded from PP analyses. Similar rules apply to the Immunogenicity analysis.

4.2 Timing of Analysis

No interim analysis is planned.

There are potentially two analysis time points for this study.

- The first analysis time point when at least 117 per protocol subjects with at least one SRVGE occurring from 28 days after the third vaccine/placebo dose. This will be the primary analysis time point for the study and will be referred to as the "Primary analysis period with 117 SRVGE cases" (at least 117 cases).
- The second analysis time point is when all participating subjects reach two years of age and will be referred to as the entire study period up to 2 years of age.

If the accrual of 117 per protocol subjects doesn't happen prior to or very close to all subjects reaching two years of age, there will be only one analysis time point. The exact timing of the primary analysis including the data cutoff will be defined as the date of resolution of the 117th case. Methods for handling missing data

- In general, missing data for efficacy and immunogenicity were considered missing at random and no imputation will be done.

Handling of Screen Failures / Ineligible subjects

Subjects failing to meet inclusion criteria and those who meet exclusion criteria at screening visit will not be randomised to either of the two study arms. A listing of screen-failure subjects will be provided with the following reasons of screen failure:

- Subject not fulfilling inclusion criterion / criteria only
- Subject fulfilling the exclusion criterion / criteria only

- Subject not fulfilling inclusion criterion / criteria and fulfilling the exclusion criterion / criteria

4.3 Statistical Analysis

All statistical analysis relating to the study will be performed on statistical software, SAS version 9.2 or later.The quantitative variables will be summarised as Number of subjects, Missing data [N (Missing)], Mean, Standard Deviation [Mean (SD)], Median, Quartiles (Q1, Q3), Range, Minimum and Maximum) [Range (Min, Max)], by study arms. The qualitative variables will be summarised as Number of subjects (n) and percentage (%) by study arms. All data used in summaries will be listed.

4.3.1 Derived Data

Baseline Values:

Baseline value of any parameter is defined as the latest non-missing value of the parameter on or before the date of administration of dose 1 of study vaccine/placebo

Date of birth:

For Date of Birth (DOB) if day and/or month are missing then the day/month will not be imputed and Date of Birth will be treated as missing for age calculation. If exact DOB is missing but the age is provided by parents then the provided age will be used.

Assessment window:

In general, visit-specific evaluations will be taken as nominal visit value without any consideration of window days around the visit day.

4.3.2 Multiplicity Consideration

For the primary endpoint, no adjustment for multiplicity is required since only a single confidence interval will be constructed.

Multiple confidence intervals will be constructed to assess the secondary efficacy, safety, and immunogenicity endpoints. No adjustment for multiple comparisons is planned.

4.3.3 Subject Disposition

The number of screened subjects will be provided without reference to study arms. The Number and percentage of subjects who were screen failures, along with the reason of screen failure will be provided. The number of subjects randomised to the study, along with number and percentage of subjects randomised to each study arms, will be provided.

Randomised subjects are those subjects who have been randomised to one of the study arms but may or may not have taken at least one dose of vaccine/placebo. Thus randomised subjects will include dropouts before first dose administration of study vaccine /placebo.

The analysis cohorts will be summarised by study arms for all randomised subjects who have taken at least one dose of vaccine/placebo.

A subject is called a completer for primary analysis period if the subject did not prematurely discontinue the study up to the time of primary analysis period.

A subject is called a completer for final analysis if he/she has completed the final study visits, i.e. did not prematurely discontinue up to 2 years of age. Premature discontinuation from the study will be summarised by study arms along with reasons for discontinuation, using all randomised subjects. In this study, the subjects for whom further doses of study vaccine/placebo are not given due to safety / other reason but if subject's parents are ready to continue in the study then that subject will be followed till end of the study to collect safety data and will be categorized as "Discontinued further vaccination but followed-up for safety". This data will be filtered from "End of The Study" CRF.

The summary of important protocol deviations (PD) provided in Table 3 will be provided by study arms using the ITT population. All protocol deviations captured during the study conduct will be provided as listing.

The number and percent of subjects in each analysis population will be provided by study arms.

4.3.4 Baseline Characteristics

Demography (age, sex, and weight at birth), Health status, Physical examination, Vital signs,Medical history, as well as household and parental characteristics, will be assessed at the screening visit. Length, Weight, Physical examination and Vital sign will be done at all visits when the clinical visit is scheduled i.e. Visit 1, Visit 2 (Week 14 visit), Visit 3 (Week 18 visit), Visit 4 (Week 18 visit, after 28 days post-Dose3), Visit 5 (6 months age visit),Visit 6 (9 months age visit), Visit 7 (12 months age visit), Visit 8 (18 months age visit), Visit 9 (24 months age visit i.e. End of the study visit).

Baseline characteristics like age, sex, and weight at birth, physical examination, vital signs, and medical history will be summarised by study arms.

4.3.4.1 Demographics

This analysis will be presented for ITT, PP, and Immunogenicity cohort.

Demographic data will include Date of Birth, Gender and Weight at birth. The type of variable, and the corresponding analysis, which will be provided by study arms, is presented in the table given below:

Variable	Type of Variable	Type of Analysis	Unit of Measurements	Formulae for Derived Variable
Age (Derived variable)	Quantitative	Summary Statistics by study arms	days	Age (days) = [Date of Screening Visit – Date of Birth] +1<br>Age (days) = Date of first SRVGE episode – Date of birth.<br>If Date of Birth is missing but age (days) is provided in CRF then that age will be imputed.
Gender	Qualitative (Male/ Female)	Frequency and Percentage by study arms	Not applicable	--
Length at Baseline	Quantitative	Summary Statistics by study arms	Cms.	--
Weight at birth	Quantitative	Summary Statistics by study arms	Kgs.	--
Weight at Baseline	Quantitative	Summary Statistics by study arms	Kgs.	--

4.3.4.2 Medical History

This will include immunization history, any ongoing diarrhea or other illness. Also it will include past medical history, surgical history, previous hospitalizations, history of any allergy to food, drugs, and current medication history,.

Subjects will be assessed at screening visit for any conditions under medical history and pre-existing conditions.

Medical History will be those medical events which would have occurred during past and stopped before administration of dose 1 of study vaccine/placebo. Pre-existing conditions are those medical events which are ongoing at the time of administration of dose 1 of study vaccine/placebo. If the stop date is missing or partial such that the stop date of the event could not be determined unambiguously with respect to date

of administration of dose 1 of study vaccine/placebo, the event will be considered as Pre-existing condition.

Medical history and pre-existing conditions will be coded using the MedDRA dictionary, Version 16.0.The exact version of the dictionary will be mentioned in the footnote of the respective Listing and/or Table. Medical history and pre-existing conditions of all the subjects will be summarised by SOC (System Organ Class) and preferred term and study arms. For this analysis both the PP and ITT analysis population will be used.

4.3.4.3 Medications/Therapies pre- treatment

Medications will be coded as per the WHO Drug Dictionary. The WHO Drug Name (Preferred Term) will be taken as the 5th Level Term Chemical Substance and the ATC Class is the 1st Level ATC. The version of the WHO DD used will be 'Version March - 2013'. The exact version will be mentioned in the footnote of the respective Listing and/or Table.

Prior medications/therapies pre-treatment will be classified as follows:

- Prior medications/therapies stopped before administration of first dose of study vaccine / placebo.

Medications/Therapies that were stopped before the start of the vaccination are referred to as Prior medications/therapies stopped before vaccination.

- Prior medications/therapies ongoing at the time of administration of first dose of study vaccine / placebo

Medications/Therapies that were started before the administration of first dose of study vaccine / placebo and were continuing at the time of administration of first dose of study vaccine / placebo are referred to as Prior medications/therapies ongoing at the time of administration of dose 1 of study vaccine/placebo. If the stop date is missing or partial such that the stop date of the medication could not be determined unambiguously with respect to date of administration of dose 1 of study vaccine/placebo, the medication will be considered as Prior medications/therapies ongoing at the time of administration of first dose of study vaccine / placebo.

Prior medications/therapies for each of the above classifications will be summarized by ATC class and WHO Drug Name (Preferred Term) by study arms. For this analysis, PP and ITT analysis population will be used.

4.3.5 Efficacy Analysis

For efficacy analysis

- For Per Protocol analysis population SRVGE / RVGE / Serotype SRVGE cases will be considered if SRVGE / RVGE / Serotype SRVGE first case occurs from 28 days after the 3rd dose [4 Weeks post

Dose 3] of study vaccine/placebo [Start date of GE – Date of 3rd dose of study vaccine/placebo + 28] among subjects receiving the full vaccine/placebo regimen

- For ITT analysis population the first SRVGE / RVGE / Serotype SRVGE case occurring from 28 days after the 3rd dose of study vaccine/placebo [Start date of GE – Date of 3rd dose of study vaccine/placebo + 28)] will be considered irrespective of number of doses administered.
- For Dose – response population the first SRVGE / RVGE / Serotype SRVGE case occurring from 28 days after last dose of study vaccine/placebo received [Start date of GE – Date of last dose study vaccine/placebo + 28)] will be considered irrespective of number of doses administered.

4.3.5.1 Primary Efficacy Analysis

**Statistical Methods used for Primary and Secondary Analysis**
Statistical Method used for Binomial Proportion for each treatment group: XXX
Statistic Represented for following Analysis:<br>n = Total Number of subjects with at least one case<br>N = Total number of subjects in each study groups in respective cohort<br>F = Total length of follow-up across all subjects in person years<br><br>Incidence Rate per Person Year Follow-up = n/F<br>Incidence Rate per 100 Person Year Follow-up = (n/F)*100<br>n/N (Proportion) along with two-sided 95% CI XXX<br><br>Follow up for each subject (days) (ITT analysis population) = Minimum [Date of first case of respective endpoint from 1st Dose, Date of premature study discontinuation, Date of study completion depending on subject status] – [Date of Dose 1 vaccine administration]<br><br>Follow up for each subject (days) (PP analysis population) = Minimum [Date of first case of respective endpoint from 28 days post 3rd Dose, Date of premature study discontinuation, Date of study completion depending on subject status] – [Date of Dose 3 vaccine administration +28 (i.e. Week 18 Date)]
Statistical Method used for Vaccine Efficacy: Cox Proportional Hazard Model

Table 4: Efficacy Analysis Endpoints

Efficacy Analysis	Population	No. of Doses	Correct Dosing	Start of F/U for Endpoint*	GE Episode	Episode Considered for Analysis	Timing of Analysis
Primary Efficacy	PP (based on protocol specified vaccination window)	3	Required	28 days post/3	SRVGE	1st only	n=117**
Secondary	PP	3	Required	28 days post/3	RVGE (any severity)	1st only	n=117
Secondary	PP	3	Required	28 days post/3	RVGE (Vesikari ≥ 17)	1st only	n=117
Secondary	PP	3	Required	28 days post/3	RVGE (Vesikari ≥ 17)	1st only	through 24 months
Secondary	PP	3	Required	28 days post/3	SRVGE	1st only	through 12 months #
Secondary	PP	3	Required	28 days post/3	SRVGE	1st only	through 24 months ##
Secondary	PP	3	Required	28 days post/3	SRVGE	1st only	12-24 months
Serotype Specific	PP	3	Required	28 days post/3	SRVGE	1st only	n=117
Serotype Specific (at 23 months)	PP	3	Required	28 days post/3	SRVGE	1st only	through 24 months
Rotavirus Hospitalization (at n=117)	PP	3	Required	28 days post/3		1st only	n=117
All cause Hospitalization (at n=117)	PP	3	Required	28 days post/3		1st only	n=117
Hospitalization (through 23 months)	PP	3	Required	28 days post/3		1st only	through 24 months
Incidence (episode per 100 child years)	PP	3	Required	28 days post/3	SRVGE		through 24 months
Incidence (GE days per 100 child years)	PP	3	Required	28 days post/3			through 24 months

Efficacy Analysis	Population	No. of Doses	Correct Dosing	Start of F/U for Endpoint*	GE Episode	Episode Considered for Analysis	Timing of Analysis
All Severe GE episodes (regardless of rotavirus status)	PP	3	Required	28 days post/3	SGE	any	n=117
All Severe GE episodes (regardless of rotavirus status)	PP	3	Required	28 days post/3	SGE	any	through 24 months
All GE episodes (regardless of rotavirus status)	PP	3	Required	28 days post/3	GE	any	n=117
All GE episodes (regardless of rotavirus status)	PP	3	Required	28 days post/3	GE	any	through 24 months

Per Protocol analysis populations will be considered as primary datasets for testing primary endpoint.

4.3.5.2 Secondary Efficacy Analysis

The secondary endpoints related to Gastroenteritis (GE) are defined in table above Table 4. The same statistical method as the primary efficacy endpoint will be used for all secondary endpoints that are based on the first episode per subject. All SGE and GE episodes endpoints regardless of rotavirus status based on counting multiple episodes per subject were analyzed using Andersen-Gill method.

4.4.4.2.1 Immunogenicity

The Rate of Rotavirus IgA Seroresponses will be compared between vaccine and Placebo Recipients in a subset of Subjects.

A subset of subjects will be selected across the study sites to examine the immunogenicity of the vaccine being tested in the study. Sufficient subjects will be enrolled in this subset to ensure the accrual of 1320 with evaluable paired sera.

Sera will be obtained before vaccination and four weeks after the third dose and tested for rotavirus antibody in an enzyme-linked immunosorbent assay (ELISA) IgA assay. The sera will also be used to test for poliovirus antibodies in neutralization assays.

The following immunogenicity endpoints for the immune responses to rotavirus and polio antibodies will be analyzed:

Rotavirus antibody in an enzyme-linked immunosorbent assay (ELISA) IgA assay:

- The number and percentage (%) of subjects with ≥3-fold increase in anti-rotavirus IgA titers from baseline to post-vaccination
- The number and percentage (%) of subjects with ≥4-fold increase in anti-rotavirus IgA titers from baseline to post-vaccination
- The number and percentage (%) of subjects whose rotavirus IgA titers are ≥ 20 IU/ml post-vaccination.
- Anti-rotavirus IgA GMT at pre and post-vaccination in all subjects regardless of baseline value and by baseline sero-status (i.e. seropositive defined as value ≥20 and seronegative defined as value <20)

The geometric mean titer and the corresponding two-sided 95% CI for rotavirus IgA and polio Neutralization assays pre and post vaccination will be summarized by study arm and compared between the study arms. Two-sided 95% CIs for GMTs and the GMT ratios between the study arms will be constructed based on t-distribution. Two-sided 95% CIs for the GMTs and the ratios of GMTs between study arms will beconstructed using log normal distribution. The log10 titers will be used to construct a CI using t-distribution for the mean and the mean difference. The mean and mean difference; and the corresponding CI limits were then exponentiated to obtain the GMT and the GMT ratio, respectively; and the corresponding CI

4.3.6 Safety Analysis

The analysis population for all safety analysis will be Safety analysis population.

All safety analysis will be summarised by study arm over time.

Any AE will be summarized as n (%) E, by study groups.

where n is the number of subjects who experienced that particular AE post each vaccination and overall

% Percentages w.r.t. number of subjects who are at risk post that particular vaccination and overall study

E Total number of episodes allowing for multiple episodes per subject for particular AE experienced post each vaccination and overall study.

In case of more than one AE with same preferred term (PT), if Stop date of the AE that occurred first is same as start date of AE when it appears second time (i.e. AE with Same PT term) then it will be treated

as same AE and will be considered only once with maximum severity grading and final outcome and worst relatedness. If AEs are not contiguous then the second AE will be considered as “New” AE.

Severity grading of adverse events

Reactions (Protocol specified terms)	Severity grade	Observation
Diarrhea (>3 loose stools/day)	1 (Mild)	At least 3 looser-than normal stools without dehydration
	2 (Moderate)	diarrhea with some dehydration (per IMNCI definition)
	3 (Severe)	diarrhea with severe dehydration (per IMNCI definition)
	4 (Life-Threatening)	diarrhea with hypovolemic shock
Vomiting	1 (Mild)	Transient or intermittent vomiting with no or minimal interference with oral intake
	2 (Moderate)	Frequent episodes of vomiting with no or mild dehydration
	3 (Severe)	Persistent vomiting resulting in orthostatic hypotension OR Aggressive rehydration indicated (e.g., IV fluids)
	4 (Life-Threatening)	Life-threatening consequences (e.g., hypotensive shock)
Fever (Axillary temperature of ≥ 37.5°C)	1 (Mild)	≥ 37.5°C to < 38.5°C
	2 (Moderate)	≥ 38.5°C to < 39.5 °C
	3 (Severe)	≥ 39.5°C to < 40.5 °C
	4 (Life-Threatening)	≥ 40.5°C
Decreased appetite (Anorexia)	1 (Mild)	Loss of appetite without decreased oral intake
	2 (Moderate)	Loss of appetite associated with decreased oral intake without significant weight loss
	3 (Severe)	Loss of appetite associated with significant weight loss
	4 (Life-Threatening)	Life-threatening consequences OR Aggressive intervention indicated [e.g., tube feeding or total parenteral nutrition (TPN)]

Reactions (Protocol specified terms)	Severity grade	Observation
Decreased activity level	1 (Mild)	Slightly subdued, but responds normally to stimuli
	2 (Moderate)	Subdued and does not respond as readily as normal to stimuli
	3 (Severe)	Lethargic
	4 (Life-Threatening)	Obtunded
Irritability	1 (Mild)	Crying more than usual but easily consoled
	2 (Moderate)	Crying more than usual and somewhat difficult to console
	3 (Severe)	Continuous crying that is inconsolable
	4 (Life-Threatening)	

- Duration(Days): Resolution Date of AE – Start Date of AE + 1
- Onset (Days) from Dose j: Start Date of AE – Vaccine administration Date of dose j + 1

4.3.6.1.1 Immediate Post-vaccination Reactions (within 30 min post vaccination)

Immediate Adverse Events (IAEs) at each dose are defined as adverse events occurring within 30 minutes post each Rotavirus (BRV-PV) / Placebo Vaccination.

IAEs will be coded using the MedDRA dictionary, Version 16.0 or later (except for Immediate Solicited Reactions). This exact version of the dictionary will be mentioned in the footnote of the respective Listing and/or Table.

The IAEs will be coded using the categories of Preferred Term (PT) and System Order Class (SOC) from MedDRA. In case the preferred term for any Event is not available then Preferred Term will be replaced by Lower Level Term (LLT), and if the LLT is also not available in such cases the CRF Reported Term for the IAEs will be considered as Preferred Term in the tables.

The IAEs will be summarized as n (%), by BRV-PV and Placebo group for each vaccine dose and for overall study (Only for all 3 doses combined), as follows:

- IAEs by System Organ Class (SOC) and Preferred term (PT).

- IAEs by Severity and Relatedness
- All IAEs by outcome.
- IAEs of Grade 3 and Grade 4 severity by SOC and Preferred Term. (Only for all 3 doses combined)
- Related IAES by SOC and PT (Only for all 3 doses combined)
- Fatal IAEs by SOC and PT. In this case "Outcome" field in the CRF marked as "Fatal" will be filtered. (Only for all 3 doses combined)
- IAEs leading to discontinuations of further vaccine doses by SOC and PT. In this case "Action taken for further dose" field in the CRF marked as "withdrawn" will be filtered. (Only for all 3 doses combined)

4.3.6.1.2 Adverses Events (AEs)

The AEs - diarrhea, vomiting, fever, decreased appetite, ear pain, skin irritation, acute respiratory infection, decreased activity level, irritability will be observed from the time of the first dose to 28 days post-Dose 3. Analysis of AE will include Immediate AEs and SAEs. GE data will be analyzed separately.

The AEs will be summarized as n(%)E, by BRV-PV and Placebo group for each vaccine dose and for safety population , as follows:

- AEs as per Protocol Specified Term in descending order of % w.r.t Rotavirus (BRV-PV) vaccine group.
- AEs by Protocol Specified Term and by severity. For this analysis the AEs with maximum intensity will be considered for each subject. AEs are considered to be related.
- AEs by outcome.
- AEs of Grade 3 and Grade 4 severity by SOC and Preferred Term. (Only for all 3 doses combined)
- Fatal AEs In this case "Outcome" field in the CRF marked as "Fatal" will be filtered. (Only for all 3 doses combined)
- AEs leading to discontinuations of further vaccine Doses. In this case "Action taken for further dose" field in the CRF marked as "withdrawn" will be filtered (Only for all 3 doses combined).

Severity for AE (excluding GE data)

Events	Severity grade	Observation
Events	1 (Mild)	Transient or mild discomfort (< 48 hours); no medical intervention/therapy required
	2 (Moderate)	Mild to moderate limitation in activity - some assistance may be needed; no or minimal medical intervention/therapy required
	3 (Severe)	Marked limitation in activity, some assistance usually required; medical intervention/therapy required, hospitalizations possible
	4 (Life Threatening)	Any adverse experience that places the child, in the view of the investigator, at immediate risk of death from the reaction as it occurred. (The investigator should not grade a reaction as life-threatening if had it occurred in a more severe form then it might have caused death.)

- Relationship to Investigational Product

  Related

  Not related

- Duration(Days): Resolution Date of AE – Start Date of AE + 1
- Onset (Days) from Dose j: Start Date of AE – Vaccine administration Date of dose j + 1
- Outcome
  - Recovered
  - Recovered with sequelae
  - Ongoing till the end of study
  - Lost to follow-up/Unknown
  - Fatal

The adverse event term will be coded using the categories of preferred term (PT) and System Order Class (SOC) from MedDRA. In case the preferred term for any Event is not available then Preferred Term will be replaced by Lower Level Term (LLT), and if the LLT is also not available in such cases the CRF Reported Term for the adverse event will be considered as Preferred Term in the tables.

4.3.6.2 GE Episodes

GE analysis will be presented separately. The analysis will be same as AE, only severity will be based on adapted Vesikari scoring and not the severity judged by investigator.

GE is defined as the passing of three or more watery or looser-than-normal stools within 24-hour period with or without vomiting and the GE Resolution Date will be the date of last ≥ 3 looser than normal stools before five consecutive diarrhea free days (≤ 2 looser than normal stools). **Diarrhea, Vomiting, Temperature**

- **Diarrhea:** Episodes with maximum of between 1 and 3 stools per day are given a score of 1, 4 or 5 stools per day a score of 2, 6 or more stools per day a score of 3.
- **Vomiting**: Episodes with a maximum of 1 vomit per day are given a score of 1, 2 to 4 vomiting episodes per day a score of 2, 5 or more vomiting episodes per day a score of 3.
- **Temperature:** Axillary Temperatures are collected in the database which will be converted to the rectal equivalent*. Episodes with maximum temperature below 37.1°C rectal equivalent are given 0 points; 37.1-38.4°C 1 point; 38.5-38.9°C 2 points; ≥39.0°C 3 points.

Rectal Equivalent Conversion:

1) Convert the temperature to Fahrenheit

• $T_F = (9/5*T_c)+32$

2. Add 2 degrees for Axillary (1 degree for oral or otic)

3. Covert back to Celsius

• $T_c = (5/9)*(T_F -32)$

Duration of Diarrhea and Vomiting Episodes

- **Duration of Diarrhea**: Number of days the subject had ≥3 Loser than Normal stool for the particular Episode between the start and Stop Date of GE Event
- **Duration of Vomiting:** Number of days the subject had Vomiting for the particular episode between the start and Stop Date of GE Event

Dehydration:

This measure is a composite score and includes four different parameters; sunken eyes, general condition, level of thirst, and skin turgor.

The assessment is based on WHO evaluation criteria to categorize dehydration in order to direct treatment. The criteria for assessing dehydration are as follows.

Severe dehydration (combination of two of the following signs):

- Lethargic or unconscious
- Sunken eyes
- Not able to drink or drinking poorly
- Skin pinch goes back very slowly

Some dehydration (combination of two of the following signs):

- Restless, irritable
- Sunken eyes
- Drinks eagerly, thirsty
- Skin pinch goes back slowly

No dehydration:

- Not enough signs to classify as some or severe dehydration

The process for determining the category of dehydration is as follows:

1. If a subject meets the criteria for severe dehydration, then the participant is classified as “severe” i.e. Score 3 (≥ 6 %) for the purposes of Vesikari scoring.

2. If the subject does not meet the severe dehydration classification, then subject is assessed for some dehydration and classified as “moderate” i.e. Score 2 (1 – 5%) if he/she meets the criteria defined.
3. If the subject does not meet the severe or some dehydration classification, then subject is considered to have no dehydration i.e. Score 0 (N/A).

**Number of Plan B events** (Moderate)	**Number of Plan C events** (Severe)	**Dehydration Category**	**Dehydration Category Score**
Any	≥ 2	>=6%	3
≥ 1	1 (not sunken eyes)	1-5%	2
≥ 2	1 (Sunken eyes)	1-5%	2
≥ 2	0	1-5%	2
1 (Sunken eyes)	1(Sunken eyes)	N/A	0
1	0	N/A	0
0	1	N/A	0
0	0	N/A	0

Treatment

If the subject is hospitalized for at least 24 hours then the score of 2 will be considered, else in case subject either receives Intravenous therapy or Oral Rehydration therapy without hospitalization then Score 1 will be considered.

The following endpoints will be assessed for GE.

- Severity and Relationship
  - Severity for GE data:

**Vesikari Score**	**Severity grade**
<7	Mild
7 -10	Moderate
≥11	Severe

  - Relationship to Investigational Product

Related

Not related

- Onset (Days) from Dose j: Start Date of GE – Vaccine administration Date of dose j + 1
- If Serious: Outcome
    - Recovered
    - Recovered with sequelae
    - Lost to follow-up/Unknown
    - Fatal

4.3.6.2.1 Occurrence of GE Post 28 Days after Third Vaccination

All GEs for which onset day is post 28 days after 3rd vaccination will be filtered from GE module of CRF. For Dose wise analysis of GE, (Onset (Days) of GE from Dose j should be > 28). For this analysis PP and ITT will be used.

For GE analysis, "Infections and infestations" will be considered as SOC and "Gastroenteritis" as Preferred Term.

The GEs will be summarized as n(%)E unless specified, by BRV-PV and Placebo group for each vaccine dose and overall study for Safety population, as follows:

- GEs by severity and relatedness. If for any GE severity by Vesikari score is missing will be treated as missing.
- Fatal GEs. In this case "Outcome" field in the CRF marked as "Fatal" will be filtered.

4.3.6.3 Deaths, Serious Adverse Events and Significant Adverse Events

4.3.6.3.1 Deaths, Serious Adverse Events

For this analysis Safety population will be used.

SAEs will be captured throughout the study.

The endpoints assessed for SAE are

- Duration(Days): Resolution Date of SAE – Start Date of SAE + 1
- Onset of SAE (Days) from Dose j: Start Date of SAE – Vaccine administration Date of dose j + 1
- Classification of SAE by SAE Code:
    - 1 - Results in death
    - 2 - Is life threatening
    - 3 - Results in persistent or significant disability/incapacity

- 4 - Requires in-patient hospitalization or prolongation of existing hospitalization
- 5 - Is a congenital anomaly/birth defect in the offspring of a study subject
- 6 - Is an important medical event that may jeopardize the subject or may require intervention to prevent one of the other outcomes listed above should be considered serious.

- Relationship to Investigational Product (Responses as mentioned above for AE)
- Death as Outcome

SAEs will be coded using MedDRA dictionary. All SAEs will be classified on the basis of Preferred Term and System Organ Class (SOC). In case the preferred term for any Event is not available then Preferred Term will be replaced by Lower Level Term (LLT), and if the LLT is also not available in such cases the CRF reported term (if available) for the serious adverse event will be considered as Preferred Term in the tables. For GE "Infections and infestations" will be considered as SOC and "Gastroenteritis" as Preferred Term.

The SAEs will be summarized as n(%)E unless specified, by BRV-PV and Placebo group for each vaccine dose and for overall study, as follows:

- SAEs by SOC and by preferred term.
- SAEs by Preferred term in descending order of % w.r.t Rotavirus (BRV-PV) vaccine group.
- SAEs by severity Related SAEs. For this analysis out of all SAEs experienced by subjects, SAEs with Relatedness marked as "Related" will be considered for each subject. In case if relatedness is missing then will be considered as "Related".
- SAEs leading to discontinuations of further vaccine Doses.
- SAEs by outcome.
- Number and percentage of subjects who died (fatal SAE) will be tabulated for each study group.
- Separate listings of fatal SAEs (with outcome=Death), and non-fatal SAEs will be provided.

For Death (Fatal SAEs) tables will be presented as follows:

- These will be presented as overall that is up to 28 days post last vaccination for all doses combined (this will consider subjects who received at least one dose of vaccination) and for each dose.

- o These will be presented as overall that is > 28 days post last vaccination up to age 2-year of the age of subject for all doses combined (this will consider subjects who received at least one dose of vaccination).

- o These will be presented as overall that is up to age 2-year of the age of subject for all doses combined (this will consider subjects who received at least one dose of vaccination).

4.3.6.3.2 Intussusception

The intussusception cases, "Confirmed by Intussusception Adjudication Committee" will be presented. For this analysis Safety population will be used.

- Number and percentage of subjects experiencing SAEs - Intussusception, will be tabulated for each study group.
- Intussusception by Brighton Collaboration criteria (Diagnostic criteria 1,2,3) as determined by the Intussusception Adjudication Committee.
- Intussusception by severity and Relatedness. For this analysis the SAEs with maximum intensity will be considered for each subject.
- Related Intussusceptions cases.
  For this analysis out of all Intussusceptions events, the events with Relatedness marked as "Related" will be considered for each subject. Intussusceptions leading to discontinuations of further vaccine Doses.
- Intussusceptions by outcome
- Duration of Intussusceptions event from last dose

For intussusception tables will be presented as follows:

These will be presented as overall Listing of intussusceptions will also be provided separately.

The safety analysis (planned / additional) related to all types of AEs for which tables will be prepared and the filtration that will be done for analysis is explained in the table below; The occurrence of at least one AE (any category) will be compared between BRV-PV and Placebo group using Fisher's Exact test.

**Type of AEs**	**Cohort / Population**	**SAEs included in Analysis**	**Time Point for Data to be included in Analysis**
IAEs	Safety Population	Included (planned as per Protocol)	Within 30 minutes post each vaccination.

Type of AEs	Cohort / Population	SAEs included in Analysis	Time Point for Data to be included in Analysis
			IAE data will be taken from IAE CRF (Form 8) and confirmed with CRF for administration of doses (Forms 7AB).<br><br>Date of onset of IAE is same as Date of Vaccination (Dose 1 / Dose 2 / Dose 3)
AEs (any severity) up to 28 days post last vaccination	Safety population	Included (planned as per Protocol)	Captured from Dose 1 vaccination (post) up to 28 days post Dose 3 Vaccination.<br><br>Data captured on AE CRF.<br><br>AE Start Date and Time ≥ Dose 1 Date and Time<br>and<br>AE Start Date and Time – Date and Time of Last dose of vaccination +1 should be ≤ 28

Type of AEs	Cohort / Population	SAEs included in Analysis	Time Point for Data to be included in Analysis
GE up to 28 Days after Third Vaccination	Safety population	Included (planned as per Protocol)	Captured from Dose 1 vaccination (post) up to 28 days post Dose 3 Vaccination.<br>Data captured on GE module of CRF.GE Start Date ≥ Dose 1 Date<br>and<br>GE Start Date and Time – Date and Time of Last dose of vaccination +1 should be ≤ 28
Death and Intussusception within 28 days post last vaccination	Safety population	Included (planned as per Protocol)	Captured from Dose 1 vaccination (post) up to 28 days post Dose 3 Vaccination.<br><br>Death (Fatal SAEs) and intussusception cases, “Confirmed by Intussusception Adjudication Committee” will be used<br><br>AE Start Date and Time ≥ Dose 1 Date and Time<br>and<br>AE Start Date and Time – Date and Time of Last dose

Type of AEs	Cohort / Population	SAEs included in Analysis	Time Point for Data to be included in Analysis
			of vaccination +1 should be ≤ 28
Death and Intussusception > 28 Days post last vaccination up to 2 yrs. of age i.e. Up to End of the Study	Safety population	Included (planned as per Protocol)	Captured from > 28 Days post last Vaccination up to end of the Study<br><br>Death (Fatal SAEs) and intussusception cases, "Confirmed by Intussusception Adjudication Committee" will be used<br><br>AE Start Date and Time – Date and Time of Last dose of vaccination +1 should be > 28<br>and<br>Severity ≥ 3
Serious Adverse Event (any severity) From Post Dose 1 up to 2 yrs. of age i.e. Up to End of the Study<br>This will also be presented separately for Death and Intussusception	Safety population	Included (planned as per Protocol)	Captured post Dose 1 up to end of the Study<br><br>Data captured on Reactogenicity, GE and Unsolicited AE module of CRF.<br><br>AE Start Date and Time ≥ Dose 1 Date and Time

4.3.6.4 Vital Signs

The vital signs captured in CRF are Weight, Length, Axillary Temperature, Heart Rate and Respiratory Rate. Listing of vital signs will be provided.

All the Vital signs will be tabulated and analyzed by study arms. All quantitative variables will be summarised as N, Mean, SD, Q1, Median, Q3 and Range (Max - Min) by study arm and visit wise.

Qualitative data i.e. Axillary Temperature, Heart Rate and Respiratory Rate: Vital signs will be categorized as “Normal (N)”, “Abnormal- Not Clinically Significant (NCS)”, “Abnormal- Clinically Significant (CS)”, “Not Done” or “Missing” and summarised as n (%) by study arm and visit wise.

Change in vital signs at each vaccine administration visit will be calculated w.r.t vital signs prior to administration of Rotavirus / Placebo vaccine for that respective dose.

4.3.6.5 Physical Examinations

Physical examination includes assessment of Head and Neck, Eye, Ears Nose and Throat, Skin, Musculoskeletal, Central Nervous System, Respiratory, Cardiovascular, Gastrointestinal and Genitourinary is captured in CRF for each clinical visit.

Listing of physical examinations will be provided.

All these examinations are qualitative results and will be classified as “Normal (N)”, “Abnormal- Not Clinically Significant (NCS)”, “Abnormal- Clinically Significant (CS)”, “Not Done” or “Missing” and will be summarized as N (%) by study groups and visit wise.

4.3.6.6 Clinical Laboratory Evaluation

Not Applicable

4.3.6.7 Concomitant Medications

Medications/therapies will be coded as per the WHO DD. The WHO Drug Name (Preferred Term) will be taken as the 5th Level Chemical Substance Term and the ATC Class is the 1st Level ATC term. The Version of the WHO DD used will be Version March 2013 or later. The exact version will be mentioned in the footnote of the respective Listing.

Medications/Therapies that were started on or after the start of the study treatment are referred to as Concomitant Medications. If the start date of the medication/therapy is missing or ambiguous such that the status of concomitancy cannot be determined, but the stop date is after the start of study treatment, the medication/therapy will be considered as Concomitant Medication. If above conditions cannot be determined unambiguously, the medication/therapy will be considered Concomitant Medication.

Details of cconcomitant medications including each medication such as, dose, frequency, route, etc. will be contained in the data listing and will not be summarized / tabulated.

4.3.7 Quality of Life Analysis

Not Applicable

4.3.8 Pharmacokinetics and Pharmacodynamic Analysis

Not applicable

5 Evaluation of Treatment Compliance and Exposure

5.1 Exposure to Study Drug

5.1.1 Extent of Exposure

The number of subjects who received number of doses of BRV-PV/Placebo and childhood vaccines will be summarized as frequency and percentage.

Even tables will be presented for PP (Protocol) to summarize number of doses received by the per protocol analysis population as per defined windows.

5.2 Subgroup / Exploratory Analysis

5.3 Effect modification of all endpoints will be explored by child sex, maternal IgA, birth weight, gestational age, breastfeeding status, concurrent diarrhea, and malnutrition status. Statistical Analytical Issues

Since Confidence Interval for Difference in Proportion using Agresti and Caffo method is not available in SAS 9.2 and also in NCSS software, Hence Wilson score method available in SAS will be used for calculating the Confidence Interval for Difference in proportion.

6 Interim Analysis and Safety Monitoring Analysis

6.1 Interim Analysis

No formal Interim Analyses are planned. However, at the time of the primary analysis based on the accrual of 117 per protocol SRVGE cases, follow-up of some safety and secondary efficacy endpoints will be completed. These preliminary interim analyses will be updated once the study is completed. The final conclusion of these analyses will be based on the complete data.

6.2 Safety Monitoring Analysis

DSMB will be appointed for review of safety data.

The DSMB will be authorized to unblind the treatment allocation if required during the trial.

The DSMB may unblind under the following conditions:

- An unanticipated serious adverse event that has been judged to be related to the BRV-PV by either the principal investigator/designee.

- A serious adverse event that has been judged as related to the BRV-PV and is of a severity or frequency higher than anticipated.
- An anticipated or other SAE where the management of the adverse event may depend on the intervention received.
- Other conditions where the DSMB may feel the need to unblind.

For each DSMB review meeting blinded data analysis will be provided as per approved mock displays approved for DSMB report.

The additional analysis for safety monitoring may be provided to DSMB members if required by DSMB members.

7 Statistical Tables to be generated

Following are the minimum tables presented in Statistical analysis

14.1 DEMOGRAPHIC AND BASELINE DATA SUMMARY

Table 14.1.1 Summary of Subject Disposition - Screened Subjects

Table 14.1.2 Summary of Subject Disposition by Study Groups - Randomized Subjects

Table 14.1.3 Summary of Premature Discontinuation by Study Groups - ITT Population

Table 14.1.4 Summary of Major Protocol Deviations by Study Groups - ITT Population

Table 14.1.5 Summary of Demographics and Baseline Characteristics by Study Groups - ITT Population

Table 14.1.7 Summary of Demographics and Baseline Characteristics by Study Groups - Immunogenicity Cohort

Table 14.1.8 Summary of Baseline Vital Signs by Study Groups – ITT Population

Table 14.1.9 Summary of Baseline Physical Examinations by Study Groups – ITT Population

Table 14.1.10 Summary of Baseline Characteristics - Medical History by Study groups – ITT Population

Table 14.1.11 Summary of Baseline Characteristics - Pre-Existing Conditions by Study Groups – ITT Population

14.2 EFFICACY TABLES

Table. Distribution of SRVGE / RVGE / Serotype SRVGE cases over calendar time, overall and by strain and severity. Table 14.2.1 Overview of Gastroenteritis (GE) Reported by Subjects and Episodes at the Time of Primary Analysis when First 117 SRVGE Cases Accrued

Table 14.2.2 Overview of Gastroenteritis (GE) Reported by Subjects and Episodes up to Two Years of Age (≤ 24 Months – End of Study)

Table 14.2.3 Efficacy of BRV-PV Vaccine against Severe RVGE (Primary Endpoint - SRVGE) at the Time of Primary Analysis when First 117 SRVGE Cases Accrued – PP Populations

Table 14.2.4 Efficacy of BRV-PV Vaccine against Severe RVGE (Primary Endpoint - SRVGE) at the Time of Primary Analysis when First 117 SRVGE Cases Accrued – ITT Population

Table 14.2.5 Efficacy of BRV-PV Vaccine against Severe RVGE (Primary Endpoint - SRVGE) up to One Year (≤ 12 Months) of Age

Table 14.2.6 Efficacy of BRV-PV Vaccine against Severe RVGE (Primary Endpoint - SRVGE) from 1-year (> 12 Months) of age up to 2-years
(≤ 24 Months) of age

Table 14.2.7 Efficacy of BRV-PV Vaccine against Severe RVGE (Primary Endpoint - SRVGE) up to Two Years of Age (≤ 24 Months – End of Study)

Table 14.2.8 Efficacy of BRV-PV Vaccine against RVGE of any Severity (Secondary Endpoint) at the Time when First 117 SRVGE Cases Accrued

Table 14.2.9 Efficacy of BRV-PV Vaccine against Severe RVGE Cases matched with G Serotypes (Secondary Endpoint) at the Time when First 117 SRVGE Cases Accrued

Table 14.2.10 Efficacy of BRV-PV Vaccine against Severe RVGE Cases matched with G Serotypes (Secondary Endpoint) up to Two Years of Age (≤ 24 Months – End of Study)

Table 14.2.11 Summary of Efficacy of BRV-PV vaccine Against SRVGE by Genotype (Secondary Endpoint) at the Time when First 117 SRVGE Cases Accrued

Table 14.2.12 Summary of Efficacy of BRV-PV vaccine Against SRVGE by Genotype (Secondary Endpoint) up to Two Years of Age (≤ 24 Months – End of Study)

Table 14.2.13 Efficacy of BRV-PV Vaccine against Hospitalization Cases due to SRVGE (Secondary Endpoint) at the Time when First 117 SRVGE Cases Accrued

Table 14.2.14 Efficacy of BRV-PV Vaccine against Hospitalization Cases due to SRVGE (Secondary Endpoint) up to Two Years of Age (≤ 24 months – End of Study)

Table 14.2.15 Vaccine Efficacy of BRV Vaccine on incidence of RVGE (Impact & Benefit - Rate per 100 Person Years, Secondary Endpoint) up to Two One Years of Age (≤ 24 Months – End of Study)

Table 14.2.16 Efficacy of BRV-PV Vaccine against Severe GE Episodes Irrespective of the presence of Rotavirus (Secondary Endpoint) at the Time when First 117 SRVGE Cases Accrued

Table 14.2.17 Efficacy of BRV-PV Vaccine against Severe GE Episodes Irrespective of the presence of Rotavirus (Secondary Endpoint) up to Two Years of Age (≤ 24 months – End of Study)

Table 14.2.18 Efficacy of BRV-PV Vaccine against Any GE Episodes (Secondary Endpoint) at the Time when First 117 SRVGE Cases Accrued
Table 14.2.19 Efficacy of BRV-PV Vaccine against Any GE Episodes (Secondary Endpoint) up to Two Years of Age (≤ 24 months – End of Study)
Table 14.2.20 Summary of Percentage (%) of Subjects with ≥ 3-fold Increase with respect to Baseline in Rotavirus IgA Titers at Day 28 (+ 7 Days) Post Dose 3, by Study Groups – Immunogenicity Cohort
Table 14.2.21 Summary of Percentage (%) of Subjects with ≥ 4-fold Increase with respect to Baseline in Rotavirus IgA Titers at Day 28 (+ 7 Days) Post Dose 3, by Study Groups – Immunogenicity Cohort
Table 14.2.22 Summary of Percentage (%) of Seropositive and Seronegative Subjects at Baseline and at Day 28 (+ 7 Days) Post Dose 3, by Study Groups and Visits – Immunogenicity Cohort
Table 14.2.23 Summary of GMTs of rotavirus IgA antibodies and 95% CI by Study groups – Immunogenicity Cohort
Table 14.2.24 Summary of GMTs of Polio Antibodies for Type 1, Type 2 and Type 3 Polio Virus Titers by Study Groups – Immunogenicity Cohort
Table 14.2.25 Percentage of Subjects with titer levels ≥ 1:8 dilution (Seroprotective Level) of Polio Antibodies for Type 1, Type 2 and Type 3 Polio Virus Titers by Study Groups and Visits – Immunogenicity Cohort
Table 14.2.26 Seroconversion Rate of Polio Antibodies for Type 1, Type 2 and Type 3 Polio Virus Titers by Study Groups and Visits – Immunogenicity Cohort

14.3.1 Immediate Adverse Events (Immediate Vaccine Reactions)

Table 14.3.1.1 Overview of Immediate Adverse Events: All Three Doses Combined – Safety Population
Table 14.3.1.2 Overview of Immediate Adverse Events: After Dose 1 – Safety Population
Table 14.3.1.3 Overview of Immediate Adverse Events: After Dose 2 – Safety Population
Table 14.3.1.4 Overview of Immediate Adverse Events: After Dose 3 – Safety Population
Table 14.3.1.5 Immediate Adverse Event by MedDRA Coding (SOC and PT): All Three Doses Combined – Safety Population
Table 14.3.1.6 Immediate Adverse Event by MedDRA Coding (SOC and PT): After Dose 1 – Safety Population
Table 14.3.1.7 Immediate Adverse Event by MedDRA Coding (SOC and PT): After Dose 2 – Safety Population
Table 14.3.1.8 Immediate Adverse Event by MedDRA Coding (SOC and PT): After Dose 3 – Safety Population
Table 14.3.1.9 Incidence of Immediate Adverse Events by Severity, Relatedness and Study Groups: Dose Wise- Safety Population

Table 14.3.1.10 Summary of Severity Grade 3 and 4 - Immediate Adverse Events by MedDRA Coding (SOC and PT): All Three Doses Combined - Safety Population

Table 14.3.1.11 Summary of Related Immediate Adverse Events by MedDRA Coding (SOC and PT): All Three Doses Combined - Safety Population

Table 14.3.1.12 Incidence of Immediate Adverse Events by Outcome and Study Groups: Dose Wise - Safety Population

Table 14.3.1.13 Summary of Fatal Immediate Adverse Events, by MedDRA Coding (SOC and PT): All Three Doses Combined - Safety Population

Table 14.3.1.14 Summary of Immediate Adverse Events Leading to Discontinuations, by MedDRA Coding (SOC and PT): All Three Doses Combined - Safety Population

14.3.2 Adverse Events– Reactogenicity Cohort

Table 14.3.2.1 Overview of Solicited Adverse Events: All Three Doses Combined

Table 14.3.2.2 Overview of Solicited Adverse Events: After Dose 1

Table 14.3.2.3 Overview of Solicited Adverse Events: After Dose 2

Table 14.3.2.4 Overview of Solicited Adverse Events: After Dose 3

Table 14.3.2.5 Incidence of Solicited Adverse Events (of the Symptoms Listed in the Protocol): All Three Doses Combined

Table 14.3.2.6 Incidence of Solicited Adverse Events (of the Symptoms Listed in the Protocol): After Dose 1

Table 14.3.2.7 Incidence of Solicited Adverse Events (of the Symptoms Listed in the Protocol): After Dose 2 –

Table 14.3.2.8 Incidence of Solicited Adverse Events (of the Symptoms Listed in the Protocol): After Dose 3 – Reactogenicity Cohort

Table 14.3.2.9 Incidence of Solicited Adverse Events by Severity and Study Groups: Dose Wise

Table 14.3.2.10 Summary of Severity Grade 3 and 4 - Solicited Adverse Events by Study Group: All Three Doses Combined

Table 14.3.2.11 Incidence of Solicited Adverse Events by Outcome and Study Groups: All Three Doses Combined Table 14.3.2.12 Summary of Fatal Solicited Adverse Events by Study Group: All Three Doses Combined Table 14.3.2.13 Solicited Adverse Events leading to Discontinuation by Study Groups

Table 14.3.2.14 Count of Solicited Adverse Events within 7 Days of Each Vaccination- Overall

14.3.3 Unsolicited Adverse Events

14.3.3.1 Unsolicited Adverse Events Up to 28 Days Post Last Dose - Reactogenicity Cohort

Table 14.3.3.1.1 Overview of Unsolicited Adverse Events Up to 28 Days Post Last Dose

Table 14.3.3.1.2 Overview of Unsolicited Adverse Events: After Dose 1 and Prior to Dose 2

Table 14.3.3.1.3 Overview of Unsolicited Adverse Events: After Dose 2 and Prior to Dose 3

Table 14.3.3.1.4 Overview of Unsolicited Adverse Events: up to 28 Days after Dose 3

Table 14.3.3.1.5 Unsolicited Adverse Event by MedDRA Coding (SOC and PT) up to 28 Days Post Last Dose

Table 14.3.3.1.6 Unsolicited Adverse Event by MedDRA Coding (SOC and PT) up to 28 Days Post Last Dose

Table 14.3.3.1.7 Unsolicited Adverse Event by MedDRA Coding (SOC and PT): After Dose 1 and Prior to Dose 2

Table 14.3.3.1.8 Unsolicited Adverse Event by MedDRA Coding (SOC and PT): after Dose 2 and Prior to Dose 3

Table 14.3.3.1.9 Unsolicited Adverse Event by MedDRA Coding (SOC and PT): up to 28 Days after Dose 3

Table 14.3.3.1.10 Unsolicited Adverse Event by MedDRA Coding (PT) up to 28 Days Post Last Dose

Table 14.3.3.1.11 Unsolicited Adverse Event by MedDRA Coding (PT): after Dose 1 and Prior to Dose 2

Table 14.3.3.1.12 Unsolicited Adverse Event by MedDRA Coding (PT): After Dose 2 and Prior to Dose 3

Table 14.3.3.1.13 Unsolicited Adverse Event by MedDRA Coding (PT): up to 28 Days after Dose 3

Table 14.3.3.1.14 Incidence of Unsolicited Adverse Events up to 28 Days Post Last Dose by Severity, Relatedness and Study Groups: Dose Wise

Table 14.3.3.1.15 Summary of Severity Grade 3 and 4 Unsolicited Adverse Events up to 28 Days Post Last Dose by MedDRA Coding (SOC and PT)

Table 14.3.3.1.16 Summary of Related Unsolicited Adverse Events up to 28 Days Post Last Dose by MedDRA Coding (SOC and PT)

Table 14.3.3.1.17 Incidence of Unsolicited Adverse Events up to 28 Days Post Last Dose by Outcome and Study Groups: Dose Wise

Table 14.3.3.1.18 Summary of Fatal Unsolicited Adverse Events up to 28 Days Post Last Dose by MedDRA Coding (SOC and PT)

Table 14.3.3.1.19 Unsolicited Adverse Events up to 28 Days Post Last Dose Leading to Discontinuation by MedDRA Coding (SOC and PT)

Table 14.3.3.1.20 Count of Unsolicited Adverse Events up to 28 Days Post Last Dose – Overall

14.3.3.2 Grade 3 or Higher or Serious Unsolicited Adverse Events Up to 28 Days Post Last Dose – Safety Population

Table 14.3.3.2.1 Overview of Grade 3 or Higher or Serious Unsolicited Adverse Events Up to 28 Days Post Last Dose – Safety Population

Table 14.3.3.2.2 Overview of Grade 3 or Higher or Serious Unsolicited Adverse Events: After Dose 1 and Prior to Dose 2 – Safety Population

Table 14.3.3.2.3 Overview of Grade 3 or Higher or Serious Unsolicited Adverse Events: After Dose 2 and Prior to Dose 3 – Safety Population

Table 14.3.3.2.4 Overview of Grade 3 or Higher or Serious Unsolicited Adverse Events: up to 28 Days after Dose 3 – Safety Population

Table 14.3.3.2.5 Grade 3 or Higher or Serious Unsolicited Adverse Event by MedDRA Coding (SOC and PT) up to 28 Days Post Last Dose – Safety Population

Table 14.3.3.2.6 Grade 3 or Higher or Serious Unsolicited Adverse Event by MedDRA Coding (SOC and PT) up to 28 Days Post Last Dose – Safety Population

Table 14.3.3.2.7 Grade 3 or Higher or Serious Unsolicited Adverse Event by MedDRA Coding (SOC and PT): After Dose 1 and Prior to Dose 2 – Safety Population

Table 14.3.3.2.8 Grade 3 or Higher or Serious Unsolicited Adverse Event by MedDRA Coding (SOC and PT): after Dose 2 and Prior to Dose 3 – Safety Population

Table 14.3.3.2.9 Grade 3 or Higher or Serious Unsolicited Adverse Event by MedDRA Coding (SOC and PT): up to 28 Days after Dose 3 – Safety Population

Table 14.3.3.2.10 Grade 3 or Higher or Serious Unsolicited Adverse Event by MedDRA Coding (PT) up to 28 Days Post Last Dose – Safety Population

Table 14.3.3.2.11 Grade 3 or Higher or Serious Unsolicited Adverse Event by MedDRA Coding (PT): after Dose 1 and Prior to Dose 2 – Safety Population

Table 14.3.3.2.12 Grade 3 or Higher or Serious Unsolicited Adverse Event by MedDRA Coding (PT): After Dose 2 and Prior to Dose 3 – Safety Population

Table 14.3.3.2.13 Grade 3 or Higher or Serious Unsolicited Adverse Event by MedDRA Coding (PT): up to 28 Days after Dose 3 – Safety Population

Table 14.3.3.2.14 Incidence of Grade 3 or Higher or Serious Unsolicited Adverse Events up to 28 Days Post Last Dose by Severity, Relatedness and Study Groups: Dose Wise - Safety Population

Table 14.3.3.2.15 Summary of Severity Grade 3 and 4 Unsolicited Adverse Events up to 28 Days Post Last Dose by MedDRA Coding (SOC and PT) – Safety Population

Table 14.3.3.2.16 Summary of Related Grade 3 or Higher or Serious Unsolicited Adverse Events up to 28 Days Post Last Dose by MedDRA Coding (SOC and PT) - Safety Population

Table 14.3.3.2.17 Incidence of Grade 3 or Higher or Serious Unsolicited Adverse Events up to 28 Days Post Last Dose by Outcome and Study Groups: Dose Wise - Safety Population

Table 14.3.3.2.18 Summary of Fatal Grade 3 or Higher or Serious Unsolicited Adverse Events up to 28 Days Post Last Dose by MedDRA Coding (SOC and PT) – Safety Population

Table 14.3.3.2.19 Grade 3 or Higher or Serious Unsolicited Adverse Events up to 28 Days Post Last Dose Leading to Discontinuation by MedDRA Coding (SOC and PT) - Safety Population

Table 14.3.3.2.20 Count of Unsolicited Adverse Events up to 28 Days Post Last Dose – Overall – Safety Population

14.3.3.3 Grade 3 or Higher or Serious Unsolicited Adverse Events after 28 Days Post Last Dose up to 2 Years of Age (End of study) – Safety Population

Table 14.3.3.3.1 Overview of Grade 3 or Higher or Serious Unsolicited Adverse Events after 28 Days Post Last Dose up to 2 Years of Age (End of study) – Safety Population

Table 14.3.3.3.2 Overview of Grade 3 or Higher or Serious Unsolicited Adverse Events after 28 Days Post Dose 3 up to 2 Years of Age (End of study) – Safety Population

Table 14.3.3.3.3 Grade 3 or Higher or Serious Unsolicited Adverse Event by MedDRA Coding (SOC and PT) after 28 Days Post Last Dose up to 2 Years of Age (End of study) – Safety Population

Table 14.3.3.3.4 Grade 3 or Higher or Serious Unsolicited Adverse Event by MedDRA Coding (SOC and PT) after 28 Days Post Dose 3 up to 2 Years of Age (End of study)– Safety Population

Table 14.3.3.3.5 Grade 3 or Higher or Serious Unsolicited Adverse Event by MedDRA Coding (PT) after 28 Days Post Last Dose up to 2 Years of Age (End of study) – Safety Population

Table 14.3.3.3.6 Grade 3 or Higher or Serious Unsolicited Adverse Event by MedDRA Coding (PT) after 28 Days Post Dose 3 up to 2 Years of Age (End of study) – Safety Population

Table 14.3.3.3.7 Incidence of Grade 3 or Higher or Serious Unsolicited Adverse Events after 28 Days Post Last Dose by Severity, Relatedness and Study Groups: Dose Wise - Safety Population

Table 14.3.3.2.8 Summary of Severity Grade 3 and 4 Unsolicited Adverse Events Post Last Dose by MedDRA Coding (SOC and PT) – Safety Population

Table 14.3.3.2.9 Summary of Related Grade 3 or Higher or Serious Unsolicited Adverse Events after 28 Days Post Last Dose by MedDRA Coding (SOC and PT) - Safety Population

Table 14.3.3.3.10 Incidence of Grade 3 or Higher or Serious Unsolicited Adverse Events after 28 Days Post Last Dose by Outcome and Study Groups: Dose Wise - Safety Population

Table 14.3.3.3.11 Summary of Fatal Grade 3 or Higher or Serious Unsolicited Adverse Events after 28 Days Post Last Dose by MedDRA Coding (SOC and PT) – Safety Population

Table 14.3.3.3.12 Grade 3 or Higher or Serious Unsolicited Adverse Events after 28 Days Post Last Dose Leading to Discontinuation by MedDRA Coding (SOC and PT) - Safety Population

Table 14.3.3.3.13 Count of Grade 3 or Higher or Serious Unsolicited Adverse Events after 28 Days Post Last Dose – Overall – Safety Population

14.3.3.4 Grade 3 or Higher or Serious Unsolicited Adverse Events Post Dose 1 – Safety Population

Table 14.3.3.4.1 Overview of Grade 3 or Higher or Serious Unsolicited Adverse Events Post Dose 1 up to 2 Years of Age (End of study) – Safety Population

Table 14.3.3.4.2 Grade 3 or Higher or Serious Unsolicited Adverse Event by MedDRA Coding (SOC and PT) Post Dose 1 up to 2 Years of Age (End of study) – Safety Population

Table 14.3.3.4.3 Grade 3 or Higher or Serious Unsolicited Adverse Event by MedDRA Coding (PT) Post Dose 1 up to 2 Years of Age (End of study) – Safety Population

Table 14.3.3.4.4 Incidence of Grade 3 or Higher or Serious Unsolicited Adverse Events Post Dose 1 up to 2 Years of Age (End of study) by Severity, Relatedness and Study Groups: Dose Wise - Safety Population

Table 14.3.3.4.5 Summary of Severity Grade 3 and 4 - Unsolicited Adverse Events Post Dose 1 up to 2 Years of Age (End of study) by MedDRA Coding (SOC and PT) – Safety Population

Table 14.3.3.4.6 Summary of Related Grade 3 or Higher or Serious Unsolicited Adverse Events Post Dose 1 up to 2 Years of Age (End of study) by MedDRA Coding (SOC and PT)) - Safety Population

Table 14.3.3.4.7 Incidence of Grade 3 or Higher or Serious Unsolicited Adverse Events Post Dose 1 up to 2 Years of Age (End of study) by Outcome and Study Groups: Dose Wise - Safety Population

Table 14.3.3.4.8 Summary of Fatal Grade 3 or Higher or Serious Unsolicited Adverse Events Post Dose 1 up to 2 Years of Age (End of study) by MedDRA Coding (SOC and PT) – Safety Population

Table 14.3.3.4.9 Grade 3 or Higher or Serious Unsolicited Adverse Events Post Dose 1 up to 2 Years of Age (End of study) Leading to Discontinuation by MedDRA Coding (SOC and PT) - Safety Population

Table 14.3.3.4.10 Count of Grade 3 or Higher or Serious Unsolicited Adverse Events Post Dose 1 up to 2 Years of Age (End of study) – Overall – Safety Population

14.3.4 Gastroenteritis Events (GE)

14.3.4.1 Gastroenteritis Events Up to 28 Days Post Last Dose - Safety Population

Table 14.3.4.1.1 Overview of Gastroenteritis Events Up to 28 Days Post Last Dose – Safety Population

Table 14.3.4.1.2 Overview of Gastroenteritis Events: After Dose 1 and Prior to Dose 2 – Safety Population

Table 14.3.4.1.3 Overview of Gastroenteritis Events: After Dose 2 and Prior to Dose 3 – Safety Population

Table 14.3.4.1.4 Overview of Gastroenteritis Events: up to 28 Days after Dose 3 – Safety Population

Table 14.3.4.1.5 Incidence of Gastroenteritis Events up to 28 Days Post Last Dose by Severity, Relatedness and Study Groups: Dose Wise - Safety Population

Table 14.3.4.1.6 Incidence of Gastroenteritis Events up to 28 Days Post Last Dose by Outcome and Study Groups: Dose Wise - Safety Population

Table 14.3.4.1.7 Fatal Gastroenteritis Events up to 28 Days Post Last Dose - Safety Population

Table 14.3.4.1.8 Gastroenteritis Events up to 28 Days Post Last Dose Leading to Discontinuation - Safety Population

Table 14.3.4.1.9 Count of Gastroenteritis Events up to 28 Days Post Last Dose – Overall –Safety Population

14.3.4.2 Gastroenteritis Events after 28 Days Post Last Dose - Safety Population

Table 14.3.4.2.1 Overview of Gastroenteritis Events after 28 Days Post Last Dose – Safety Population

Table 14.3.4.2.2 Overview of Gastroenteritis Events: After 28 Days Post Dose 1 and Prior to Dose 2 – Safety Population

Table 14.3.4.2.3 Overview of Gastroenteritis Events: After 28 Days Post Dose 2 and Prior to Dose 3 – Safety Population

Table 14.3.4.2.4 Overview of Gastroenteritis Events: 28 Days after Dose 3 – Safety Population

Table 14.3.4.2.5 Incidence of Gastroenteritis Events after 28 Days Post Last Dose by Severity, Relatedness and Study Groups: Dose Wise - Safety Population

Table 14.3.4.2.6 Incidence of Gastroenteritis Events after 28 Days Post Last Dose by Outcome and Study Groups: Dose Wise - Safety Population

Table 14.3.4.2.7 Fatal Gastroenteritis Events after 28 Days Post Last Dose - Safety Population

Table 14.3.4.2.8 Gastroenteritis Events after 28 Days Post Last Dose Leading to Discontinuation - Safety Population

Table 14.3.4.2.9 Count of Gastroenteritis Events after 28 Days Post Last Dose – Overall –Safety Population

14.3.5 Serious Adverse Events

Table 14.3.5.1 Overview of Serious Adverse Events Post Dose 1 up to 2 Years of Age (End of study) – Safety Population

Table 14.3.5.2 Overview of Serious Adverse Events: After Dose 1 and Prior to Dose 2 – Safety Population

Table 14.3.5.3 Overview of Serious Adverse Events: After Dose 2 and Prior to Dose 3 – Safety Population

Table 14.3.5.4 Overview of Serious Adverse Events: up to 28 Days after Dose 3 – Safety Population

Table 14.3.5.5 Overview of Serious Adverse Events: after 28 Days Post Dose 3 up to 2 Years of Age – Safety Population

Table 14.3.5.6 Serious Adverse Events Post Dose 1 up to 2 Years of Age (End of Study) by MedDRA Coding (SOC and PT) – Safety Population

Table 14.3.5.7 Serious Adverse Event by MedDRA Coding (SOC and PT): After Dose 1 and Prior to Dose 2 – Safety Population

Table 14.3.5.8 Serious Adverse Event by MedDRA Coding (SOC and PT): after Dose 2 and Prior to Dose 3 – Safety Population

Table 14.3.5.9 Serious Adverse Event by MedDRA Coding (SOC and PT): Up to 28 Days after Dose 3 – Safety Population

Table 14.3.5.10 Serious Adverse Event by MedDRA Coding (SOC and PT): After 28 Days Post Dose 3 up to 2 Years of Age – Safety Population

Table 14.3.5.11 Serious Adverse Events Post Dose 1 up to 2 Years of Age (End of Study) by MedDRA Coding (PT) – Safety Population

Table 14.3.5.12 Serious Adverse Event by MedDRA Coding (PT): after Dose 1 and Prior to Dose 2 – Safety Population

Table 14.3.5.13 Serious Adverse Event by MedDRA Coding (PT): after Dose 2 and Prior to Dose 3 – Safety Population

Table 14.3.5.14 Serious Adverse Event by MedDRA Coding (PT): Up to 28 Days after Dose 3 – Safety Population

Table 14.3.5.15 Serious Adverse Event by MedDRA Coding (SOC and PT): After 28 Days Post Dose 3 up to 2 Years of Age – Safety Population

Table 14.3.5.16 Incidence of Serious Adverse Events Post Dose 1 up to 2 Years of Age by Severity, Relatedness and Study Groups: Dose Wise - Safety Population

Table 14.3.5.17 Summary of Severity Grade 3 and 4 - Serious Adverse Events Post Dose 1 up to 2 Years of Age (End of study) by MedDRA Coding (SOC and PT) - Safety Population

Table 14.3.5.18 Summary of Related Serious Adverse Events Post Dose 1 up to 2 Years of Age (End of study)by MedDRA Coding (SOC and PT) - Safety Population

Table 14.3.5.19 Incidence of Serious Adverse Events Post Dose 1 up to 2 Years of Age (End of study) by Outcome and Study Groups: Dose Wise - Safety Population

Table 14.3.5.20 Incidence of Serious Adverse Events Post Dose 1 up to 2 Years of Age (End of study) by Seriousness Criteria and Study Groups - Safety Population

Table 14.3.5.21 Summary of Fatal Serious Adverse Events Post Dose 1 up to 2 Years of Age (End of study) by MedDRA Coding (SOC and PT) - Safety Population

Table 14.3.5.22 Summary of Serious Adverse Events Post Dose 1 up to 2 Years of Age (End of study) Leading to Discontinuations, by MedDRA Coding (SOC and PT) - Safety Population

Table 14.3.5.23Serious Adverse Events Counts Post Dose 1 up to 2 Years of Age (End of study) – Overall - Safety Population

Table 14.3.5.24 Incidence of Intussusception Post Dose 1 up to 2 Years of Age (End of study) - Safety Population

Table 14.3.5.25 Incidence of Intussusception up to 28 Days Post Last Dose - Safety Population

Table 14.3.5.26 Intussusception Details after 28 Days Post Last Dose Up to 2 Years – Safety Population

Table 14.3.5.27 Incidence of Intussusception up to 28 Days Post Last Dose by Intussusception Level and Study Groups: Dose Wise - Safety Population

Table 14.3.5.28 Incidence of Intussusception after 28 Days Post Last Dose by Intussusception Level and Study Groups: Dose Wise - Safety Population

Table 14.3.5.29 Incidence of Intussusception up to 28 Days Post Last Dose by Severity, Relatedness and Study Groups: Dose Wise - Safety Population

Table 14.3.5.30 Incidence of Intussusception after 28 Days Post Last Dose by Severity, Relatedness and Study Groups: Dose Wise - Safety Population

Table 14.3.5.31 Incidence of Intussusception up to 28 Days Post Last Dose by Outcome and Study Groups: Dose Wise - Safety Population

Table 14.3.5.32 Incidence of Intussusception after 28 Days Post Last Dose by Outcome and Study Groups: Dose Wise - Safety Population

Table 14.3.5.33 Fatal Intussusception Events up to 28 Days Post Last Dose - Safety Population

Table 14.3.5.34 Fatal Intussusception Events after 28 Days Post Last Dose - Safety Population

Table 14.3.5.35 Intussusception Events up to 28 Days Post Last Dose Leading to Discontinuation - Safety Population

Table 14.3.5.36 Intussusception Events after 28 Days Post Last Dose Leading to Discontinuation - Safety Population

Table 14.3.5.37 Onset and Duration (Days) of Serious Adverse Events – Safety Population

14.3.6 Other Safety Information

Table 14.3.6.1 Summary of Vital Signs by Visits and Study Groups – Safety Population

Table 14.3.6.2 Summary of Change in Vital Signs from Baseline by Visits and Study Groups – Safety Population

Table 14.3.6.3 Summary of Physical Examinations by Visits and Study Groups – Safety Population

Table 14.3.6.4 Summary of Shifts from Baseline to Respective Visits for Physical Examination by Study Group – Safety Population

Table 14.3.6.5 Summary of Extent of Exposure to BRV-PV/Placebo - ITT Population

Table 14.3.6.6 Summary of Observations after BRV-PV/Placebo administration - ITT Population

Table 14.3.6.7 Summary of Vaccine Schedule Duration in Days followed by BRV-PV/Placebo administration - ITT Population

8 Statistical Listings to be generated

14.3.2 Listing of Deaths, Other Serious and Significant Adverse Events

Listing 14.3.2.1 Listing of Fatal SAEs

Listing 14.3.2.2 Listing of SAE - Intussusceptions

Listing 14.3.2.3 Listing of Stool Sample Collected for GE Episode

Listing 14.3.2.4 Listing of Non-Fatal SAEs

16.2 Subject Data Listings

16.2.1 Subjects Disposition

Listing 16.2.1.1 Subjects who are Screen-Failure

Listing 16.2.1.2 Subjects who discontinued during the Study after Randomization

16.2.2 Protocol Deviations

Listing 16.2.2.1 Important Major Protocol Deviations During the Study

Listing 16.2.2.2 Minor Protocol Deviations During the Study

16.2.3 Subjects Excluded from ITT, Reactogenicity, PP, Immunogenicity or Safety Analysis Populations

Listing 16.2.3.1 Disposition of Subjects - All Randomized Subjects

Listing 16.2.3.2 Exclusion of Data from Per Protocol Population - All Randomized Subjects

Listing 16.2.3.3 Exclusion of Data from Immunogenicity Cohort - All Randomized Subjects

16.2.4 Demographics and Baseline Characteristics

Listing 16.2.4.1 Demographic Data

Listing 16.2.4.2 Baseline Characteristics – Health Status

Listing 16.2.4.3 Baseline Characteristics – Medical History and Pre-existing Conditions

Listing 16.2.4.4 Baseline Characteristics – Prior Medications - I of II

Listing 16.2.4.4 Baseline Characteristics – Prior Medications - II of II

16.2.5 Compliance and Study Drug Exposure Data

Listing 16.2.5.1 Study Drug Dose Exposure – Vaccine Administration Details - I of II

Listing 16.2.5.1 Study Drug Dose Exposure – Vaccine Administration Details - II of II

16.2.6 Individual Serology and Immunogenicity Response Data

Listing 16.2.6.1 Gastroenteritis (GE) cases - I of II

Listing 16.2.6.1 Gastroenteritis (GE) cases - II of II

Listing 16.2.6.2 Immunogenicity Endpoint - Poliovirus antibody Titers in Neutralization Assays (Polio Antibodies)

Listing 16.2.6.3 Immunogenicity Endpoint – Rotavirus Antibodies in ELISA IgA Assays

16.2.7 Adverse Events Listings

Listing 16.2.7.1 Immediate Adverse Events (IAEs) Listings – I of II

Listing 16.2.7.1 Immediate Adverse Events (IAEs) Listings – II of II

Listing 16.2.7.2 Solicited Adverse Events Listings for Reactogenicity Cohort – I of II

Listing 16.2.7.2 Solicited Adverse Events Listings for Reactogenicity Cohort – II of II

Listing 16.2.7.3 Unsolicited Adverse Events Listings– I of II

Listing 16.2.7.3 Unsolicited Adverse Events Listings– II of II

Listing 16.2.7.4 Listing of Gastro Enteritis Episodes

16.4 Individual Subject Data Listings

Listing 16.4.1 Subject Eligibility Criteria

Listing 16.4.2 Universal Immunization Program and Other Vaccines

Listing 16.4.3 Listing of Vital Signs

Listing 16.4.4 Listing of Physical examination

Listing 16.4.5 Concomitant Medications - I of II

Listing 16.4.5 Concomitant Medications - II of II

Listing 16.4.6 Study Completion Status

Listing 16.4.7 Final Randomization List

9 Statistical Graphs to be generated

Figure 17.1: Study Flowchart - <<For Primary Analysis / End of the Study>>

Figure 17.2 Study Flowchart: Analysis populations - <<For Primary Analysis / End of the Study>>

14.2.2 Primary and Secondary Efficacy Analysis Related Figures

Figure 14.2.2.1 Kaplan-Meier Survival Curves for SRVGE cases experienced by Subject Post 28 Days after Third Dose of BRV-PV / Placebo – PP Cohort

Figure 14.2.2.2 Kaplan-Meier Survival Curves for SRVGE cases experienced by Subject Post 28 Days after Third Dose of BRV-PV / Placebo – ITT Cohort

Figure 14.2.2.3 Kaplan-Meier Survival Curves for SRVGE cases experienced by Subject Post 28 Days after Third Dose of BRV-PV / Placebo –m ITT Cohort.

Figure 14.2.2.4 Kaplan-Meier Survival Curves for RVGE cases experienced by Subject Post 28 Days after Third Dose of BRV-PV / Placebo – PP Cohort

Figure 14.2.2.5 Kaplan-Meier Survival Curves for RVGE cases experienced by Subject Post 28 Days after Third Dose of BRV-PV / Placebo – ITT Cohort

Figure 14.2.2.6 Kaplan-Meier Survival Curves for RVGE cases experienced by Subject Post 28 Days after Third Dose of BRV-PV / Placebo – mITT Cohort

Figure 14.2.2.7 Kaplan-Meier Survival Curves for G- serotype SRVGE cases experienced by Subject Post 28 Days after Third Dose of BRV-PV / Placebo – PP Cohort

Figure 14.2.2.8 Kaplan-Meier Survival Curves for G- serotype SRVGE cases experienced by Subject Post 28 Days after Third Dose of BRV-PV / Placebo – PP Cohort

Figure 14.2.2.9 Kaplan-Meier Survival Curves for G- serotype SRVGE cases experienced by Subject Post 28 Days after Third Dose of BRV-PV / Placebo – PP Cohort

Figure 14.2.2.10 Forest Plot for Hazard Ratio on Log Scale for all Types of GE

14.2.3 Immunogenicity Analysis Related Figures

Figure 14.2.3.1 The Geometric Mean Titers (GMTs) for IgA antibody titers at Baseline and 28 days post 3rd dose of BRV-PV/ Placebo - Immunogenicity Cohort

Figure 14.2.3.2 Three-fold Responders for IgA antibody titers at 28 days post 3rd dose of BRV-PV / Placebo - Immunogenicity Cohort

Figure 14.2.3.3 Bar Chart showing Four-fold Responders for IgA antibody titers at 28 days post 3rd dose of BRV-PV / Placebo - Immunogenicity Cohort

Figure 14.2.3.4 Bar Chart showing percentage of subjects showing IgA antibody titers ≥ 20 IU/mL- Immunogenicity Cohort

14.2.3 Safety Figures

Figure 14.2.3.1 Unsolicited Severe AEs >3% by Preferred term and Study Groups - Percentage Bar Charts

Figure 14.2.3.2 SAEs >3% by Preferred term and Study Groups - Percentage Bar Charts

Figure 14.2.3.3 SAEs - Deaths by Study Groups - Percentage Bar Charts

Figure 14.2.3.4 SAEs - Intussusception by Study Groups - Percentage Bar Charts
