# Supplementary material for: Rotavirus vaccine efficacy up to 2 years of age and against diverse circulating rotavirus strains in Niger: Extended follow-up of a randomized controlled trial
Source: PLoS Med. 2021 Jul 2;18(7):e1003655. doi: 10.1371/journal.pmed.1003655 (PMC8253401; doi:10.1371/journal.pmed.1003655)
Supplement: S1 Table — (DOCX) [file pmed.1003655.s004.docx]

Table A. Characteristics of intent-to-treat participants

|  | Rotasiil | Placebo |
| --- | --- | --- |
| N | 2,044 | 2,047 |
| Age in weeks, Mean (SD) |  |  |
| At Dose 1 | 6.46 (0.65) | 6.45 (0.64) |
| At Dose 2 | 10.56 (0.96) | 10.56 (1.05) |
| At Dose 3 | 14.71 (1.28) | 14.75 (1.75) |
| At end of extended efficacy follow-up | 101.41 (14.22) | 100.96 (14.01) |
| Male, n (%) | 1026 (50.20) | 1007 (49.19) |
| Weight (kg), Mean (SD) | 4.51 (0.71) | 4.49 (0.67) |
| Length (cm), Mean (SD) | 54.30 (2.55) | 54.37 (2.42) |
| OPV co-administered, n (%) |  |  |
| At Dose 1 | 1167 (57.53) | 1203 (58.77) |
| At Dose 2 | 1103 (53.96) | 1107 (54.08) |
| At Dose 3 | 1111 (54.35) | 1146 (55.98) |

Table B. Vaccine efficacy against gastroenteritis in the intent-to-treat population, by age and severity

|  | BRV-PV (N = 2044) | | | Placebo (N = 2047) | | |  |  |
| --- | --- | --- | --- | --- | --- | --- | --- | --- |
|  | N with ≥ 1 episode | Person years | Incidence rate per 100 py | N with ≥ 1 episode | Person years | Incidence rate per 100 py | Incidence rate difference, per 100 person-years (95% CI) | Vaccine efficacy  (95% CI) |
| **Rotavirus gastroenteritis** | | | | | | | | |
| **< 1 year** |  |  |  |  |  |  |  |  |
| All | 231 | 1652.27 | 13.98 | 366 | 1587.49 | 23.06 | -9.07 (-12.05 - -6.10) | 39.4 (28.5 – 48.6) |
| Severe | 54 | 1729.48 | 3.12 | 148 | 1687.28 | 8.77 | -5.65 (-7.29 - -4.01) | 64.4 (51.4 – 73.9) |
| Very severe | 11 | 1744.99 | 0.63 | 39 | 1737.83 | 2.24 | -1.61 (-2.41 - -8.17) | 71.9 (45.2 – 85.6) |
| **1 year to < 2 years** |  |  |  |  |  |  |  |  |
| All | 59 | 1676.68 | 3.52 | 63 | 1527.51 | 4.12 | -0.61 (-1.96 – 0.75) | 14.7 (-21.7 – 40.2) |
| Severe | 15 | 1875.96 | 0.80 | 19 | 1765.82 | 1.08 | -0.28 (-0.91 – 0.35) | 25.7 (-46.2 – 62.2) |
| Very severe | 2 | 1927.87 | 0.10 | 1 | 1879.89 | 0.05 | 0.05 (-0.13 – 0.23) | -95.0 (-2050.8 – 82.3) |
| **Total follow up** |  |  |  |  |  |  |  |  |
| All | 290 | 3328.95 | 8.71 | 429 | 3115.00 | 13.77 | -5.06 (-6.71 - -3.42) | 36.8 (26.6 – 45.5) |
| Severe | 69 | 3605.43 | 1.91 | 167 | 3453.10 | 4.84 | -2.92 (-3.78 - -2.06) | 60.4 (47.6 – 70.1) |
| Very severe | 13 | 3672.85 | 0.35 | 40 | 3617.72 | 1.11 | -0.75 (-1.15 - -0.36) | 68.0 (40.1 – 82.9) |
| **Gastroenteritis from any cause** | | | | | | | | |
| **< 1 year** |  |  |  |  |  |  |  |  |
| All | 1062 | 1214.89 | 87.42 | 1138 | 1162.99 | 97.85 | -10.44 (-18.18 - -2.69) | 10.7 (2.9 – 17.8) |
| Severe | 312 | 1629.57 | 19.15 | 396 | 1574.11 | 25.16 | -6.01 (-9.28 - -2.75) | 23.9 (11.7 – 34.4) |
| Very severe | 39 | 1733.95 | 2.25 | 102 | 1711.01 | 5.96 | -3.71 (-5.07 - -2.36) | 62.3 (45.4 – 73.9) |
| **1 year to < 2 years** |  |  |  |  |  |  |  |  |
| All | 123 | 842.817 | 14.59 | 137 | 763.926 | 17.93 | -3.34 (-7.30 – 0.62) | 18.6 (-3.8 – 36.2) |
| Severe | 53 | 1604.38 | 3.30 | 63 | 1508.70 | 4.18 | -0.87 (-2.23 – 0.49) | 20.9 (-14.0 – 45.1) |
| Very severe | 13 | 1893.46 | 0.69 | 6 | 1820.55 | 0.33 | 0.36 (-0.10 – 0.81) | -108.3 (-448.1 – 20.8) |
| **Total follow up** |  |  |  |  |  |  |  |  |
| All | 1185 | 2057.71 | 57.59 | 1275 | 1926.92 | 66.17 | -8.58 (-13.47 - -3.69) | 13.0 (5.8 – 19.6) |
| Severe | 365 | 3233.95 | 11.29 | 459 | 3082.81 | 14.89 | -3.60 (-5.39 - -1.82) | 24.2 (13.0 – 33.9) |
| Very severe | 52 | 3627.41 | 1.43 | 108 | 3531.56 | 3.06 | -1.63 (-2.32 - -0.93) | 53.1 (34.7 – 66.3) |

Table C. Characterization of circulating rotavirus strains in Madarounfa, Niger by G and P Type (August 2015 to February 2018)

| **G Type [P Type]** | **N (%)** |
| --- | --- |
|  |  |
| G1[P10] | 1 (0,08) |
| G1[P4] | 1 (0,08) |
| G1[P4 P6 P8] | 1 (0,08) |
| G1[P6] | 73 (5,91) |
| G1[P6 P8] | 1 (0,08) |
| G1[P8] | 112 (9,07) |
| G1[PND] | 2 (0,16) |
| G1 G10[P6] | 1 (0,08) |
| G1 G12[P4] | 1 (0,08) |
| G1 G12[P6] | 4 (0,32) |
| G1 G12[P8] | 12 (0,97) |
| G1 G2[P4] | 2 (0,16) |
| G1 G2[P4 P6] | 1 (0,08) |
| G1 G2[P6] | 3 (0,24) |
| G1 G2[PND] | 1 (0,08) |
| G1 G2 G12[P4 P6] | 1 (0,08) |
| G1 G2 G3[P4] | 1 (0,08) |
| G1 G9[P6] | 1 (0,08) |
| G10[P8] | 1 (0,08) |
| G12[P4] | 6 (0,49) |
| G12[P4 P8] | 1 (0,08) |
| G12[P6] | 27 (2,19) |
| G12[P6 P8] | 1 (0,08) |
| G12[P8] | 183 (14,82) |
| G12[PND] | 2 (0,16) |
| G2[P4] | 469 (37,98) |
| G2[P4 P6] | 4 (0,32) |
| G2[P6] | 5 (0,4) |
| G2[P8] | 4 (0,32) |
| G2[PND] | 12 (0,97) |
| G2 G12[P4] | 8 (0,65) |
| G2 G12[P4 P8] | 2 (0,16) |
| G2 G12[P6] | 1 (0,08) |
| G2 G12[PND] | 3 (0,24) |
| G2 G3[P4] | 3 (0,24) |
| G2 G3[P6] | 2 (0,16) |
| G2 G3[P8] | 2 (0,16) |
| G2 G9[P8] | 1 (0,08) |
| G2 G9[PND] | 1 (0,08) |
| G3[P6] | 102 (8,26) |
| G3[P8] | 2 (0,16) |
| G3[PND] | 1 (0,08) |
| G3 G12[P4] | 1 (0,08) |
| G3 G12[P6] | 1 (0,08) |
| G3 G9[P4] | 1 (0,08) |
| G3 G9[P6] | 11 (0,89) |
| G3 G9[PND] | 1 (0,08) |
| G3 G9 G10[P6] | 1 (0,08) |
| G4[P6] | 4 (0,32) |
| G4[PND] | 1 (0,08) |
| G4 G9 G12[PND] | 2 (0,16) |
| G8[PND] | 1 (0,08) |
| G8 G12[PND] | 1 (0,08) |
| G9[P4] | 5 (0,4) |
| G9[P4 P6] | 2 (0,16) |
| G9[P4 P6 P8] | 1 (0,08) |
| G9[P4 P8] | 2 (0,16) |
| G9[P6] | 25 (2,02) |
| G9[P6 P8] | 5 (0,4) |
| G9[P8] | 92 (7,45) |
| G9[PND] | 1 (0,08) |
| G9 G12[P6] | 1 (0,08) |
| G9 G12[P8] | 3 (0,24) |
| G9 G12[PND] | 2 (0,16) |
| GND[P4] | 2 (0,16) |
| GND[P6] | 3 (0,24) |
| NT[NT] | 4 (0,32) |

Table E. Strain-specific vaccine efficacy against rotavirus gastroenteritis in the intent-to-treat population, by severity

|  | BRV-PV (N = 2042) | | | Placebo (N = 2044) | | |  |  |
| --- | --- | --- | --- | --- | --- | --- | --- | --- |
|  | N with ≥ 1 episode | Person years | Incidence rate per 100 py | N with ≥ 1 episode | Person years | Incidence rate per 100 py | Incidence rate difference, per 100 person-years (95% CI) | Vaccine efficacy  (95% CI) |
| **Vaccine type (VT; G1, G2, G3, G4, G9)** |  |  |  |  |  |  |  |  |
| All | 213 | 2985.91 | 7.13 | 352 | 2779.45 | 12.66 | -5.53 (-7.16 - -3.90) | 43.7 (33.2 – 52.5) |
| Severe | 50 | 3191.72 | 1.57 | 138 | 3053.55 | 4.52 | -2.95 (-3.82 - -2.08) | 65.3 (52.1 – 74.9) |
| Very severe | 10 | 3240.57 | 0.31 | 33 | 3187.77 | 1.04 | -0.73 (-1.13 - -0.33) | 70.2 (39.5 – 85.3) |
| **G1 alone** |  |  |  |  |  |  |  |  |
| All | 35 | 3220.31 | 1.09 | 67 | 3164.74 | 2.12 | -1.03 (-1.65 - -0.41) | 48.7 (22.7 – 65.9) |
| Severe | 7 | 3245.39 | 0.22 | 20 | 3210.47 | 0.62 | -0.41 (-0.72 - -0.09) | 65.4 (18.1 – 85.4) |
| Very severe | 0 | 3254.10 | 0 | 1 | 3231.86 | 0.03 | -0.03 (N/A – N/A) | 100.0 (N/A – N/A) |
| **G2 alone** |  |  |  |  |  |  |  |  |
| All | 134 | 3071.38 | 4.36 | 212 | 2942.51 | 7.20 | -2.84 (-4.06 – 01.62) | 39.4 (24.8 – 51.2) |
| Severe | 32 | 3210.38 | 1.00 | 85 | 3119.56 | 2.72 | -1.73 (-2.40 - -1.05) | 63.4 (45.1 – 75.6) |
| Very severe | 8 | 3242.38 | 0.25 | 26 | 3198.99 | 0.81 | -0.57 (-0.92 - -0.21) | 69.6 (33.0 – 86.3) |
| **G3 alone** |  |  |  |  |  |  |  |  |
| All | 16 | 3232.24 | 0.50 | 40 | 3175.47 | 1.26 | -0.76 (-1.22 - -0.31) | 60.7 (29.8 – 78.0) |
| Severe | 2 | 3251.23 | 0.06 | 20 | 3204.16 | 0.62 | -0.56 (-0.85 - -0.28) | 90.1 (57.8 – 97.7) |
| Very severe | 1 | 3252.77 | 0.03 | 4 | 3227.02 | 0.12 | -0.09 (-0.23 – 0.04) | 75.2 (-121.9 – 97.2) |
| **G4 alone** |  |  |  |  |  |  |  |  |
| All | 1 | 3252.65 | 0.03 | 0 | 3233.52 | 0 | 0.03 (N/A – N/A) | 0.00 (N/A – N/A) |
| Severe | 0 | 3254.10 | 0 | 0 | 3233.52 | 0 | n/a | n/a |
| Very severe | 0 | 3254.10 | 0 | 0 | 3233.52 | 0 | n/a | n/a |
| **G9 alone** |  |  |  |  |  |  |  |  |
| All | 37 | 3219.70 | 1.15 | 41 | 3189.41 | 1.29 | -0.14 (-0.68 – 0.40) | 10.6 (-39.4 – 42.7) |
| Severe | 9 | 3247.03 | 0.28 | 14 | 3218.92 | 0.43 | -0.16 (-0.45 – 0.13) | 36.3 (-47.2 – 72.4) |
| Very severe | 1 | 3253.62 | 0.03 | 2 | 3230.46 | 0.06 | -0.03 (-0.14 – 0.07) | 50.4 (-447.5 – 95.5) |
| **Mixed VT** |  |  |  |  |  |  |  |  |
| All | 9 | 3243.22 | 0.28 | 16 | 3213.83 | 0.50 | -0.22 (-0.52 – 0.08) | 44.3 (-26.1 – 75.4) |
| Severe | 3 | 3251.18 | 0.09 | 7 | 3224.42 | 0.22 | -0.12 (-0.32 – 0.07) | 57.5 (-64.4 – 89.0) |
| Very severe | 0 | 3254.10 | 0 | 2 | 3231.85 | 0.06 | -0.06 (N/A – N/A) | 100.0 (N/A – N/A) |
| **Non-vaccine type (NVT; G8 and G12)** |  |  |  |  |  |  |  |  |
| All | 46 | 3202.20 | 1.44 | 52 | 3170.97 | 1.64 | -0.20 (-0.81 – 0.41) | 12.4 (-30.3 – 41.1) |
| Severe | 12 | 3239.93 | 0.37 | 19 | 3209.25 | 0.59 | -0.22 (-0.56 – 0.12) | 37.4 (-28.9 – 69.6) |
| Very severe | 2 | 3252.14 | 0.06 | 4 | 3228.13 | 0.12 | -0.06 (-0.21 – 0.09) | 50.4 (-171.0 – 90.9) |
| **G8 alone** |  |  |  |  |  |  |  |  |
| All | 0 | 3254.10 | 0 | 1 | 3232.28 | 0.03 | -0.03 (N/A – N/A) | 100.0 (N/A- N/A) |
| Severe | 0 | 3254.10 | 0 | 1 | 3232.28 | 0.03 | -0.03 (N/A – N/A) | 100.0 (N/A – N/A) |
| Very severe | 0 | 3254.10 | 0 | 0 | 3233.52 | 0 | n/a | n/a |
| **G12 alone** |  |  |  |  |  |  |  |  |
| All | 45 | 3202.65 | 1.41 | 51 | 3172.21 | 1.61 | -0.20 (-0.81 – 0.40) | 12.6 (-30.5 – 41.5) |
| Severe | 12 | 3239.93 | 0.37 | 18 | 3210.49 | 0.56 | -0.19 (-0.52 – 0.14) | 33.9 (-37.1 – 68.2) |
| Very severe | 2 | 3252.14 | 0.06 | 4 | 3228.13 | 0.12 | -0.06 (-0.21 – 0.09) | 50.4 (-171.0 – 90.9) |
| **Mixed NVT** |  |  |  |  |  |  |  |  |
| All | 18 | 3232.82 | 0.56 | 16 | 3212.66 | 0.50 | 0.06 (-0.30 – 0.41) | -11.8 (-119.23 – 42.9) |
| Severe | 4 | 3249.07 | 0.12 | 2 | 3230.57 | 0.06 | 0.06 (-0.09 – 0.21) | -98.9 (-985.7 – 63.6) |
| Very severe | 1 | 3252.63 | 0.03 | 1 | 3231.91 | 0.03 | -0.0002 (-0.09 – 0.09) | 0.6 (-1488.5 – 93.8) |
|  |  |  |  |  |  |  |  |  |
| **P type** |  |  |  |  |  |  |  |  |
| **P4** |  |  |  |  |  |  |  |  |
| All | 134 | 3074.87 | 4.36 | 216 | 2940.38 | 7.35 | -2.99 (-4.22 - -1.76) | 40.7 (26.4 – 52.2) |
| Severe | 33 | 3208.99 | 1.03 | 85 | 3121.55 | 2.72 | -1.70 (-2.37 - -1.02) | 62.2 (43.6 – 74.7) |
| Very severe | 9 | 3240.91 | 0.28 | 26 | 3198.99 | 0.81 | -0.54 (-0.90 - -0.17) | 65.8 (27.1 – 84.0) |
| **P6** |  |  |  |  |  |  |  |  |
| All | 62 | 3175.67 | 1.95 | 114 | 3081.23 | 3.70 | -1.75 (-2.58 - -0.91) | 47.2 (28.1 – 61.3) |
| Severe | 10 | 3241.75 | 0.31 | 47 | 3170.16 | 1.48 | -1.17 (-1.64 - -0.71) | 79.2 (58.8 – 89.5) |
| Very severe | 1 | 3252.77 | 0.03 | 10 | 3219.08 | 0.31 | -0.28 (-0.48 - -0.08) | 90.1 (22.7 – 98.7) |
| **P8** |  |  |  |  |  |  |  |  |
| All | 76 | 3182.14 | 2.39 | 100 | 3129.60 | 3.20 | -0.81 (-1.63 – 0.02) | 25.3 (-0.7 – 44.5) |
| Severe | 20 | 3234.77 | 0.62 | 32 | 3196.45 | 1.00 | -0.38 (-0.82 – 0.06) | 38.2 (-8.0 – 64.7) |
| Very severe | 3 | 3251.66 | 0.09 | 4 | 3228.07 | 0.12 | -0.03 (-0.19 – 0.13) | 25.5 (-232.7 – 83.3) |

Figure A. Vaccine efficacy by follow-up period and country-level child mortality classification*


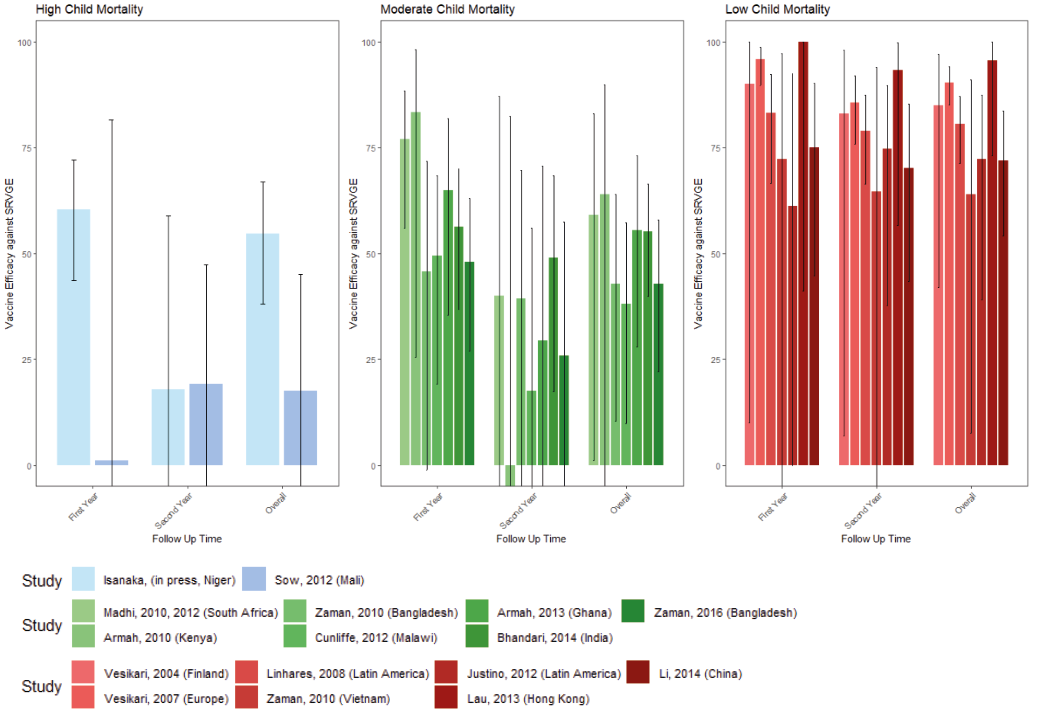


*Under-five mortality defined as per the United Nations Inter-agency Group for Child Mortality Estimation [64] as follows: low; <25 deaths per 1,000 live births; moderate: 25-50 deaths per 1,000 live births; and high: >50 deaths per 1,000 live births.

Figure includes placebo-controlled phase III efficacy studies presenting a breakdown of vaccine efficacy by follow-up period over two years.

Figure A References

1. Isanaka S, Garba S, Plikaytis BD, McNeal M, Guindo O, Langendorf C, et al. Immunogenicity of an oral rotavirus vaccine administered with prenatal nutritional support. (under review).

2. Sow SO, Tapia M, Haidara FC, Ciarlet M, Diallo F, Kodio M, et al. Efficacy of the oral pentavalent rotavirus vaccine in Mali. Vaccine. 2012;30 Suppl 1:A71-8. Epub 2012/05/02. doi: 10.1016/j.vaccine.2011.11.094. PubMed PMID: 22520140.

3. Madhi SA, Kirsten M, Louw C, Bos P, Aspinall S, Bouckenooghe A, et al. Efficacy and immunogenicity of two or three dose rotavirus-vaccine regimen in South African children over two consecutive rotavirus-seasons: a randomized, double-blind, placebo-controlled trial. Vaccine. 2012;30 Suppl 1:A44-51. doi: 10.1016/j.vaccine.2011.08.080. PubMed PMID: 22520136.

4. Armah GE, Sow SO, Breiman RF, Dallas MJ, Tapia MD, Feikin DR, et al. Efficacy of pentavalent rotavirus vaccine against severe rotavirus gastroenteritis in infants in developing countries in sub-Saharan Africa: a randomised, double-blind, placebo-controlled trial. Lancet. 2010;376(9741):606-14. Epub 2010/08/10. doi: 10.1016/s0140-6736(10)60889-6. PubMed PMID: 20692030.

5. Zaman K, Dang DA, Victor JC, Shin S, Yunus M, Dallas MJ, et al. Efficacy of pentavalent rotavirus vaccine against severe rotavirus gastroenteritis in infants in developing countries in Asia: a randomised, double-blind, placebo-controlled trial. Lancet. 2010;376(9741):615-23. Epub 2010/08/10. doi: 10.1016/s0140-6736(10)60755-6. PubMed PMID: 20692031.

6. Cunliffe NA, Witte D, Ngwira BM, Todd S, Bostock NJ, Turner AM, et al. Efficacy of human rotavirus vaccine against severe gastroenteritis in Malawian children in the first two years of life: a randomized, double- blind, placebo controlled trial. Vaccine. 2012;30 Suppl 1:A36-43. Epub 2012/05/02. doi: 10.1016/j.vaccine.2011.09.120. PubMed PMID: 22520135; PubMed Central PMCID: PMCPMC3982044.

7. Bhandari N, Rongsen-Chandola T, Bavdekar A, John J, Antony K, Taneja S, et al. Efficacy of a monovalent human-bovine (116E) rotavirus vaccine in Indian infants: a randomised, double-blind, placebo-controlled trial. Lancet. 2014;383(9935):2136-43. Epub 2014/03/19. doi: 10.1016/s0140-6736(13)62630-6. PubMed PMID: 24629994; PubMed Central PMCID: PMCPMC4532697.

8. Vesikari T, Karvonen A, Prymula R, Schuster V, Tejedor JC, Cohen R, et al. Efficacy of human rotavirus vaccine against rotavirus gastroenteritis during the first 2 years of life in European infants: randomised, double-blind controlled study. Lancet. 2007;370(9601):1757-63. doi: 10.1016/S0140-6736(07)61744-9. PubMed PMID: 18037080.

9. Vesikari T, Karvonen A, Puustinen L, Zeng SQ, Szakal ED, Delem A, et al. Efficacy of RIX4414 live attenuated human rotavirus vaccine in Finnish infants. Pediatr Infect Dis J. 2004;23(10):937-43. Epub 2004/12/17. doi: 10.1097/01.inf.0000141722.10130.50. PubMed PMID: 15602194.

10. Linhares AC, Velázquez FR, Pérez-Schael I, Sáez-Llorens X, Abate H, Espinoza F, et al. Efficacy and safety of an oral live attenuated human rotavirus vaccine against rotavirus gastroenteritis during the first 2 years of life in Latin American infants: a randomised, double-blind, placebo-controlled phase III study. Lancet. 2008;371(9619):1181-9. doi: 10.1016/S0140-6736(08)60524-3. PubMed PMID: 18395579.

11. Justino MC, Araujo EC, van Doorn LJ, Oliveira CS, Gabbay YB, Mascarenhas JD, et al. Oral live attenuated human rotavirus vaccine (Rotarix) offers sustained high protection against severe G9P[8] rotavirus gastroenteritis during the first two years of life in Brazilian children. Mem Inst Oswaldo Cruz. 2012;107(7):846-53. Epub 2012/11/14. doi: 10.1590/s0074-02762012000700002. PubMed PMID: 23147138.

12. Lau YL, Nelson EA, Poon KH, Chan PK, Chiu S, Sung R, et al. Efficacy, safety and immunogenicity of a human rotavirus vaccine (RIX4414) in Hong Kong children up to three years of age: a randomized, controlled trial. Vaccine. 2013;31(18):2253-9. Epub 2013/03/19. doi: 10.1016/j.vaccine.2013.03.001. PubMed PMID: 23499605.

13. Li RC, Huang T, Li Y, Luo D, Tao J, Fu B, et al. Human rotavirus vaccine (RIX4414) efficacy in the first two years of life: a randomized, placebo-controlled trial in China. Hum Vaccin Immunother. 2014;10(1):11-8. Epub 2013/09/10. doi: 10.4161/hv.26319. PubMed PMID: 24013441; PubMed Central PMCID: PMCPMC4181005.

14. Armah GE, Kapikian AZ, Vesikari T, Cunliffe N, Jacobson RM, Burlington DB, et al. Efficacy, immunogenicity, and safety of two doses of a tetravalent rotavirus vaccine RRV-TV in Ghana with the first dose administered during the neonatal period. J Infect Dis. 2013;208(3):423-31. Epub 2013/04/18. doi: 10.1093/infdis/jit174. PubMed PMID: 23599316; PubMed Central PMCID: PMCPMC3699001.

15. Zaman K, Sack DA, Neuzil KM, Yunus M, Moulton LH, Sugimoto JD, et al. Effectiveness of a live oral human rotavirus vaccine after programmatic introduction in Bangladesh: A cluster-randomized trial. PLoS Med. 2017;14(4):e1002282. Epub 2017/04/19. doi: 10.1371/journal.pmed.1002282. PubMed PMID: 28419095; PubMed Central PMCID: PMCPMC5395158.
